# Orthologous genes of the red flour beetle *Tribolium castaneum* and the vinegar fly *Drosophila melanogaster*

**DOI:** 10.1186/s12863-025-01397-0

**Published:** 2025-12-12

**Authors:** Noel Cabañas, Doga Cedden, Gregor Bucher

**Affiliations:** 1 Department of Evolutionary Developmental Genetics, GZMB, University of Göttingen, Göttingen, Germany; 2 International Max Planck Research School for Genome Science, Göttingen, Germany

**Keywords:** *Tribolium castaneum*, *Drosophila melanogaster*, Orthologs, Orthologous, Arthropods, Insects, Comparative genomics, iBeetleBase

## Abstract

**Objectives:**

*Tribolium castaneum and Drosophila melanogaster* are prominent insect model organisms for investigating developmental and evolutionary processes. Both have a significant kit of genetic and molecular tools and a substantial quantity of omic data at their disposal, which makes this species pair suitable for comparative genomic and gene function studies. However, for such comparisons, a rigorous assignment and compilation of the orthologs that these organisms share are essential. Here, we generated and provided a list of orthologous genes between *Drosophila* and *Tribolium*, which will be useful for future comparative genomic studies.

**Data description:**

We made use of the reference proteomes of *Tribolium castaneum* and *Drosophila melanogaster* to infer phylogenetic orthology using the OrthoFinder platform, and employed the eggNOG 6.0 database and manual phylogenetic tree analyses to assess our results. Our analysis identified more than 9,000 orthologous genes between *Drosophila* and *Tribolium.* We posit that this comprehensive list is a valuable resource for comparative studies among these insect species, such as single-cell sequencing or large-scale gene function comparisons. The results are open-access and freely available for download or interactive exploration in iBeetleBase.

**Supplementary Information:**

The online version contains supplementary material available at 10.1186/s12863-025-01397-0.

## Objective

Arthropods are a speciose group of organisms with diverse evolutionary histories [1]. To study the genomic and genetic basis of evolution, we use a small number of model systems where tools for gene function analysis have been established [2]. Among them, two prominent insect model organisms stand out: the vinegar fly *(Drosophila melanogaster)* and the red flour beetle *(Tribolium castaneum).* With respect to tools for studying gene function, *Drosophila* is the most advanced animal model system spearheading research. *Tribolium*, as a follow-up model system, possesses a wide range of genetic and molecular tools, such as genome-wide RNAi screening, transposon-mediated transgenesis, and genome editing [3–11]. Furthermore, a decent amount of genomic information has been gathered over many years of research [12–15], making *Tribolium* an excellent model for the comparison of diverse biological processes in genetics, molecular biology, evolution, and development [2, 10, 11]. Given the derived biology of *D*rosophila, *Tribolium* has also become a noteworthy point of comparison for resolving conserved processes in bilaterian organisms [16–18].

All comparative work on gene function requires a robust assignment of orthologous genes. Notwithstanding, a carefully established list of orthologs is lacking for these species, which hampers ongoing comparative approaches such as single-cell sequencing and the proper interconnection of data among FlyBase and iBeetle-Base. The lack of such a list constantly leads to unnecessary duplication of similar work and restricts the comparability of different studies. Therefore, we set out to identify the orthologous genes between *Drosophila* and *Tribolium* using the OrthoFinder platform, followed by the comparison of these results with data from the orthology database eggNOG (v6.0) and phylogenetic trees manually produced for selected genes. We expect that this list will reduce and unify the efforts required for future comparisons of the two most developed genetic model systems within insects. It will also serve as a resource to streamline molecular comparisons between these organisms in areas such as comparative genomics, single-cell genomics, embryonic development, appendage regeneration, RNAi screenings, and pest management, to mention some of them, but not limited to just these [10, 11, 16].

## Data description

We retrieved the genome assemblies of *Tribolium* (Tcas5.2) and *Drosophila* (dmel_r6.55) from iBeetleBase and FlyBase, coupled with their gene annotations and protein predictions (gene annotation OGS3 for *Tribolium* and r6.55 for *Drosophila*) [14, 17]. We then computed the orthologous and paralogous genes based on protein sequences using the automated software tool OrthoFinder (v2.5.5, using BLAST for sequence alignment and FastTree for phylogenetic tree inference with the default parameters). This program is the current gold standard tool for determining orthologous genes, and it has been shown to outperform other existing methods [19]. As a result, we identified 9,656 unique *Tribolium* genes matching 8,757 *Drosophila* genes. Among these, 6,086 are one-to-one orthologs, 465 are one-to-many orthologs from *Tribolium* to *Drosophila*, 778 are one-to-many orthologs from *Drosophila* to *Tribolium*, and 176 are many-to-many orthologs. In order to validate our results, we took advantage of the eggNOG 6.0 database (available at http://eggnog6.embl.de), which provides orthology information for comparative genomics, but it does not focus on insects, *Drosophila*, or *Tribolium* [20]. We fetched all available orthologs of *Drosophila* and *Tribolium* from eggNOG and compared these data with our OrthoFinder predictions. We found that 7,161 *Tribolium* and 7,033 *Drosophila* ortholog genes were present in both lists. We also noticed that OrthoFinder and eggNOG predicted approximately 8,000 orthologs for *Drosophila*, but for *Tribolium*, eggNOG contained only 7,654 orthologs while OrthoFinder predicted more than 9,000 orthologs. Upon further comparison, we found that 4,793 one-to-one orthology assignments were the same in both data sets, whereas 1,293 and 579 one-to-one orthologs were assigned only by OrthoFinder and eggNOG, respectively. Additionally, we manually determined the orthology of 67 genes by running the *Drosophila* gene against the BLAST non-redundant protein database. We gathered 500 sequences for each gene, removed the redundant ones using MMseqs2, aligned the sequences with MAFFT, and trimmed the alignment with trimAl. We used these alignments to generate HMMER profiles and search a database that contained proteomes for *Drosophila*, *Tribolium*, and 40 other genomes comprising the major order of insects and some outgroup proteomes such as mouse and human. We used the 500 most significant hits to generate maximum likelihood trees with IQ-TREE 2 that were visualized with iTOL [21–27]. Based on the phylogenetic trees, we assigned one-to-one orthology to 60 genes, while OrthoFinder assigned orthology to 56 genes and eggNOG to 53 genes. These analyses show examples of the validity of our genome-wide approach. The datasets containing all orthologs identified with OrthoFinder (Data set 1) [28], and the one containing the retrieved genes from eggNOG (Data set 2) are available as ready-to-explore lists [29]. Also, the manually checked genes (Data set 3) [30], and the comparison between the one-to-one orthologs computed by OrthoFinder and eggNOG (Data set 4) are available for download in the links provided in Table 1 [31]. We also embedded these data in iBeetleBase.

**Table 1:** Lists of orthologous genes of *Tribolium* and *Drosophila*

Label	Name of data file	File types (file extension)	Data repository and identifier (accession number)
*Data set 1*	*1.OrthoFinder_orthology*	*Tab-separated values (.tsv)*	***Figshare*** (10.6084/m9.figshare.27695070.v1) [28]
*Data set 2*	*2.Eggnog6_orthology*	*Tab-separated values (.tsv)*	***Figshare*** (10.6084/m9.figshare.27695190.v1) [29]
*Data set 3*	*3.manually_checked_genes*	*Compressed file (.zip)*	***Figshare*** (10.6084/m9.figshare.27695256.v1) [30]
*Data set 4*	*4.Comparison_one-to-one_orthologs*	*Excel Open XML Spreadsheet (.xlsx)*	***Figshare*** (10.6084/m9.figshare.27695316.v1) [31]

## Limitations

We provide lists of orthologous genes between *Tribolium* and *Drosophila* that are predicted according to our best knowledge of the state of the art using OrthoFinder, and compare our results with an up-to-date orthology database such as eggNOG 6.0. We resolved one-to-one orthologs for approximately 6,000 genes and one-to-many or many-to-many orthology for approximately 3,000 genes. However, 17,873 genes are predicted for *Drosophila*, and 16,593 are predicted for *Tribolium* [13, 17]. Therefore, our orthology assignment was conservative and was not able to detect orthology for numerous genes. Another drawback of our assignment is that it is focused on protein-coding genes; therefore, the orthology assignment of noncoding genes is out of the scope of this work. Moreover, future enhancements of gene annotations, e.g. based on improved genome sequencing, more transcriptomic data, or enhanced annotation algorithms, may lead to the prediction of additional orthologs. In the case that we generate better orthology assignments in the future, we will make them available on iBeetle-Base, at the side of the list published in this work. While this paper aims at detecting orthologous genes, the pool of non-orthologous genes provides an interesting set for studying the emergence and function of novel genes.

## Supplementary Information

Below is the link to the electronic supplementary material.

Supplementary Material 1

Supplementary Material 2

Supplementary Material 3

Supplementary Material 4

## Data Availability

The data described in this Data note can be freely and openly accessed on iBeetleBase under https://ibeetle-base.uni-goettingen.de/resources/, https://github.com/SalmonellaIIB/Orthologous_Tribolium_Drosophila, or 10.6084/m9.figshare.27695070.v1, 10.6084/m9.figshare.27695190.v1, 10.6084/m9.figshare.27695256.v1, 10.6084/m9.figshare.27695316.v1. Please see Table 1 for more details and links to the data.

## Abbreviations

*Tribolium*: *Tribolium castaneum*
*Drosophila*: *Drosophila melanogaster*
RNAi: RNA interference
