# Supplementary figures and images for: Orthologous genes of the red flour beetle Tribolium castaneum and the vinegar fly Drosophila melanogaster
Source: BMC Genom Data. 2025 Dec 12;27:17. doi: 10.1186/s12863-025-01397-0 (PMC12903232; doi:10.1186/s12863-025-01397-0)

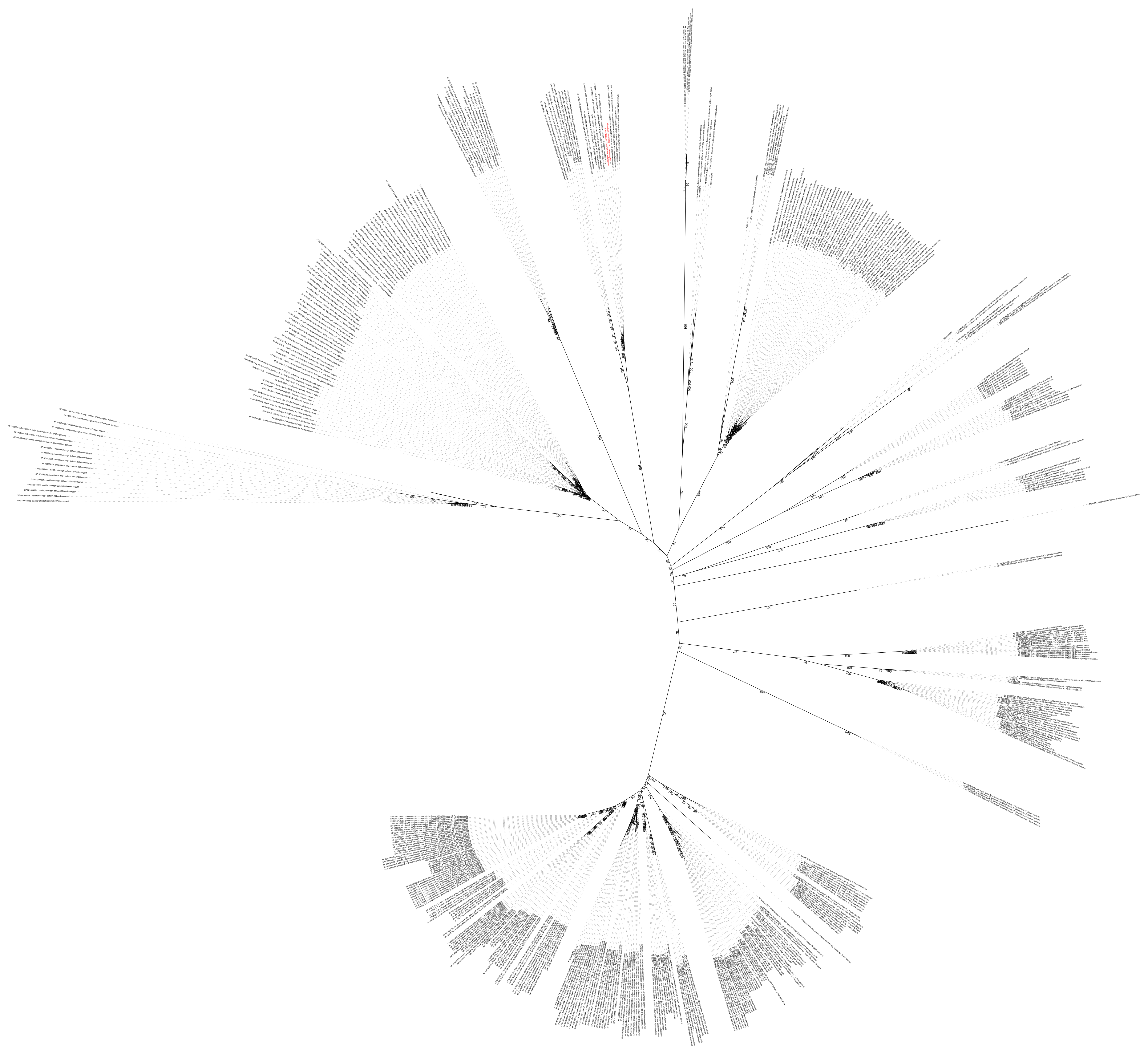

Supplement: Supplementary file 3 — Supplementary Material 3 [file 12863_2025_1397_MOESM3_ESM.zip › 3.Manually_checked_genes/4.Trees/CG34376.pdf]

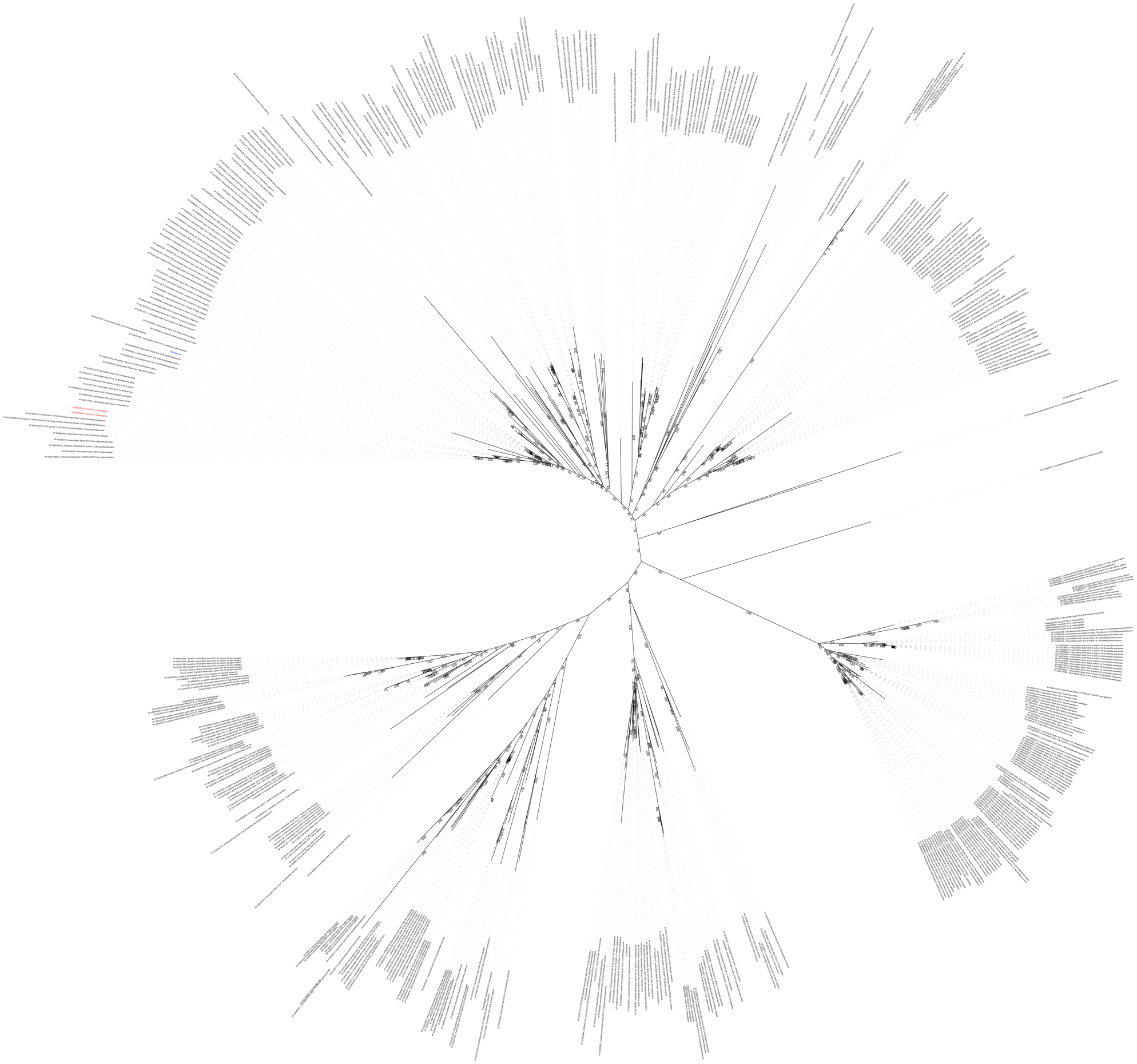

Supplement: Supplementary file 3 — Supplementary Material 3 [file 12863_2025_1397_MOESM3_ESM.zip › 3.Manually_checked_genes/4.Trees/SoxN.pdf]

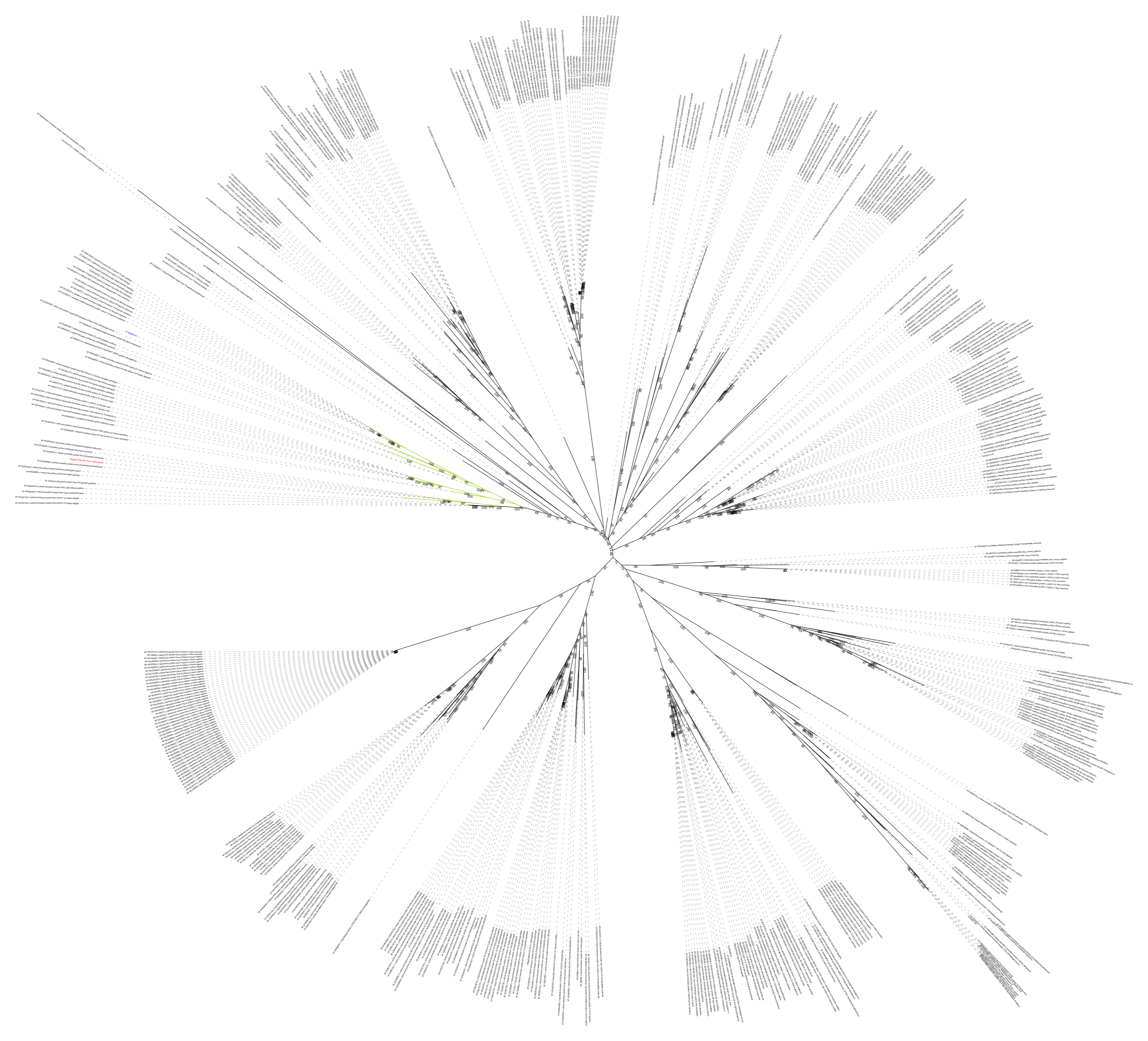

Supplement: Supplementary file 3 — Supplementary Material 3 [file 12863_2025_1397_MOESM3_ESM.zip › 3.Manually_checked_genes/4.Trees/hbn.pdf]

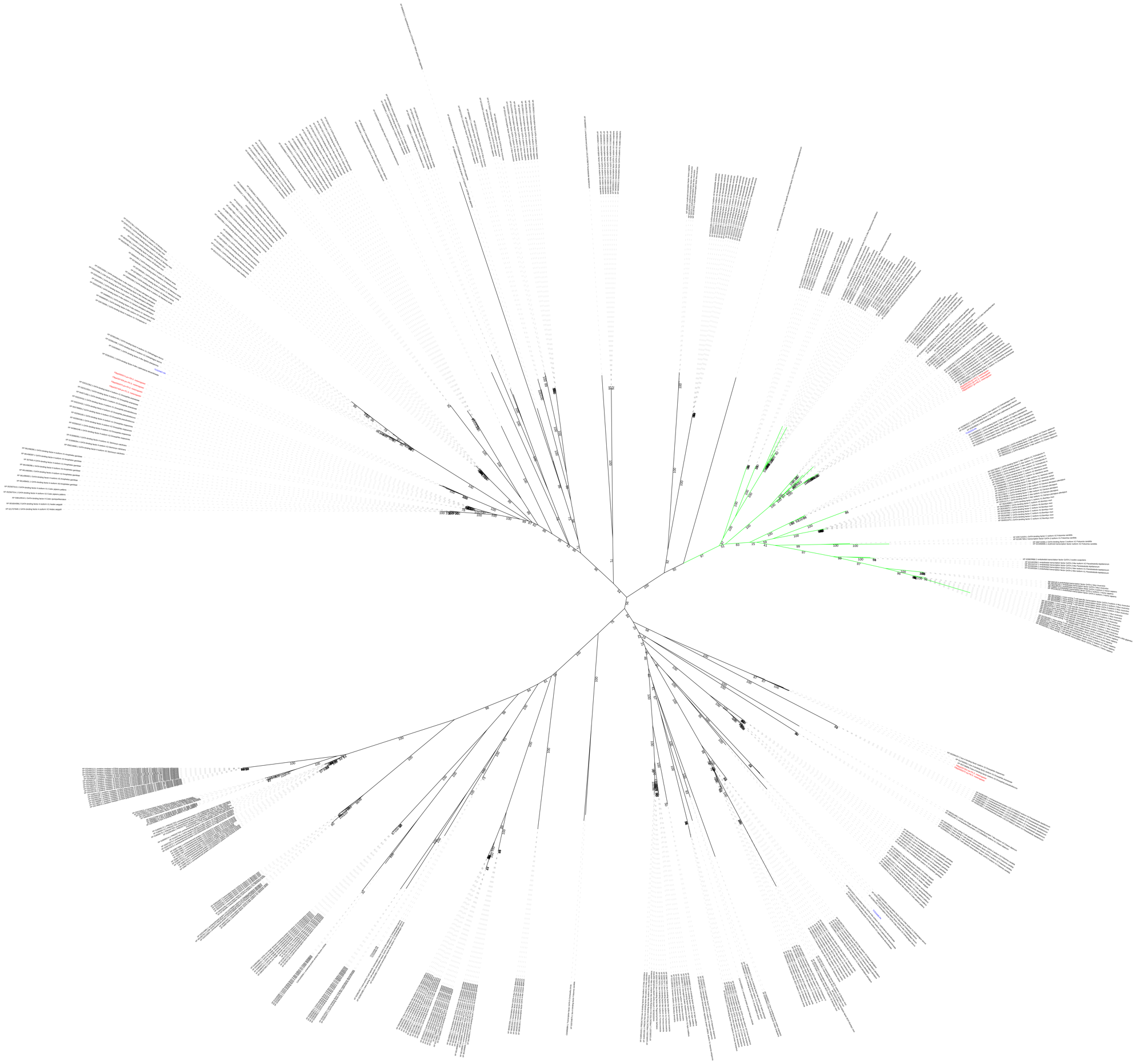

Supplement: Supplementary file 3 — Supplementary Material 3 [file 12863_2025_1397_MOESM3_ESM.zip › 3.Manually_checked_genes/4.Trees/grn.pdf]

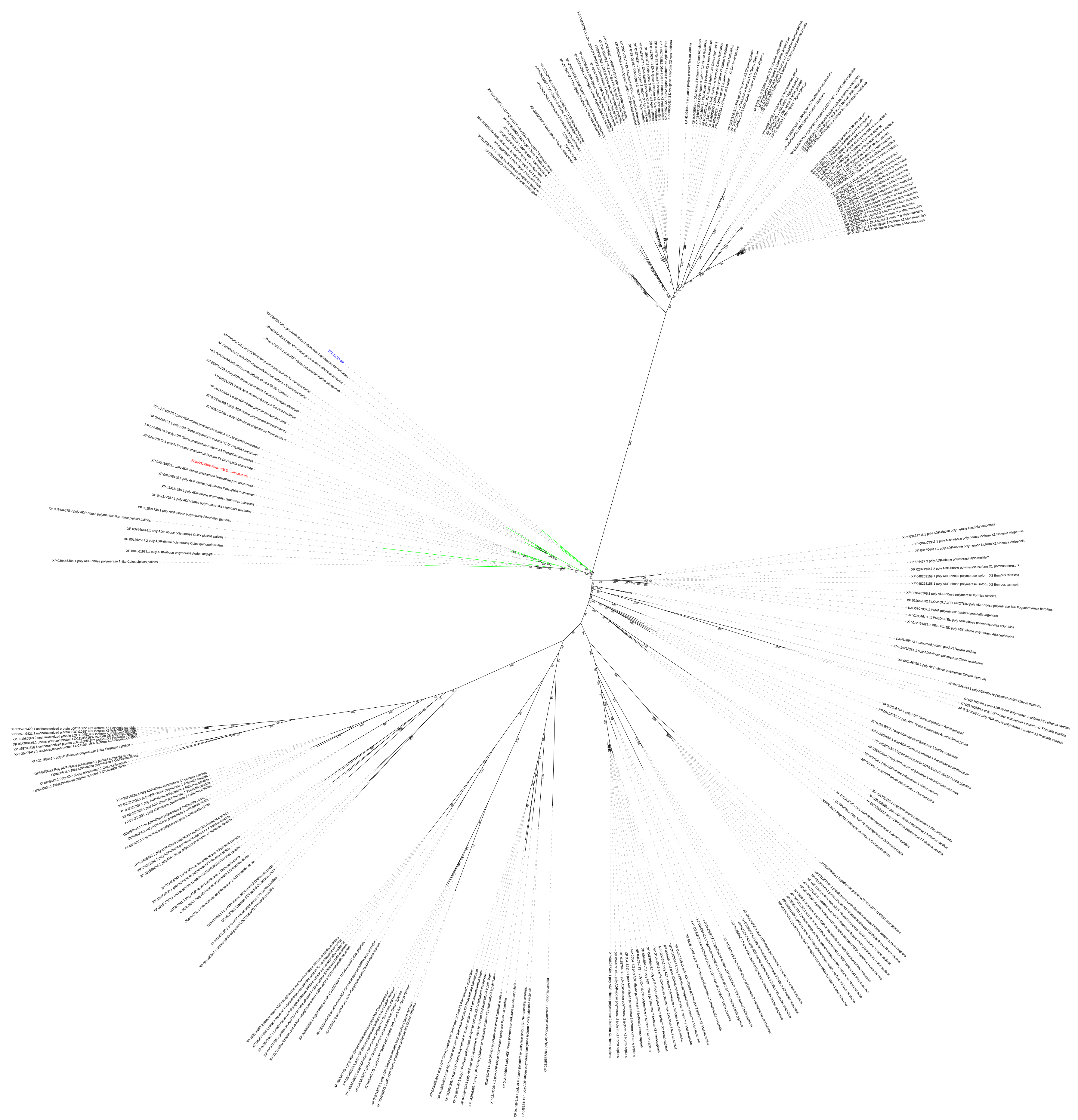

Supplement: Supplementary file 3 — Supplementary Material 3 [file 12863_2025_1397_MOESM3_ESM.zip › 3.Manually_checked_genes/4.Trees/Parp1.pdf]

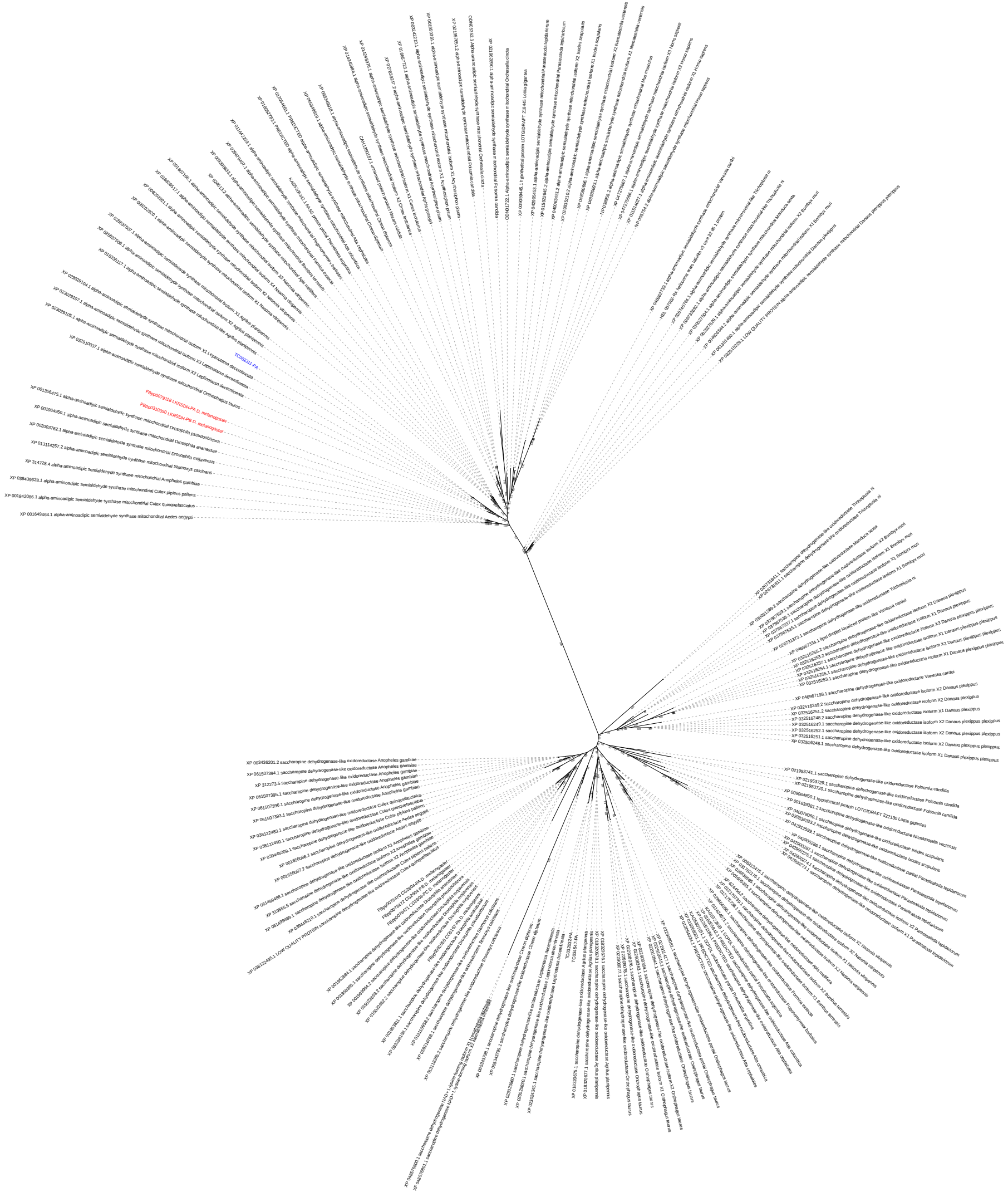

Supplement: Supplementary file 3 — Supplementary Material 3 [file 12863_2025_1397_MOESM3_ESM.zip › 3.Manually_checked_genes/4.Trees/LKRSDH.pdf]

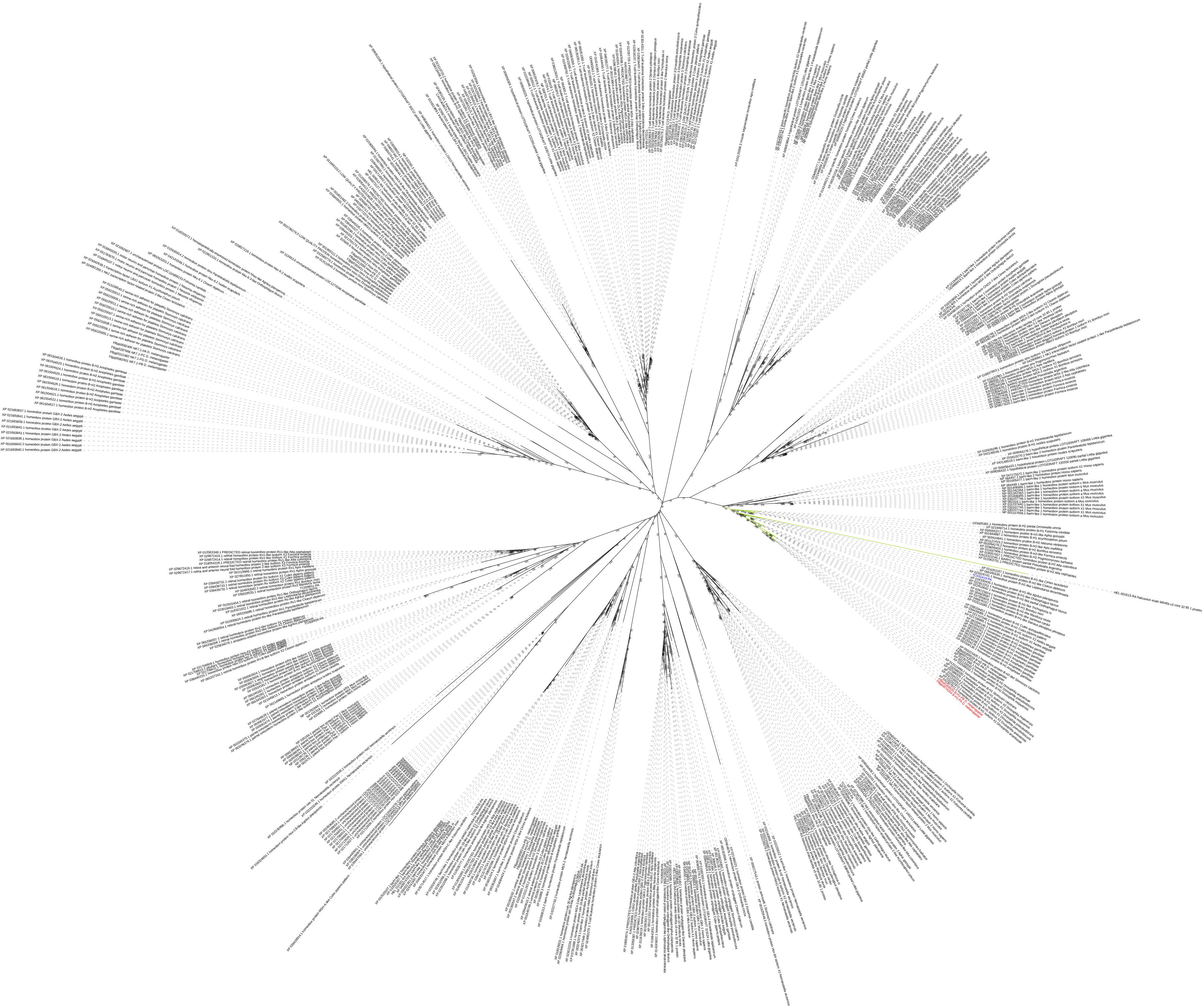

Supplement: Supplementary file 3 — Supplementary Material 3 [file 12863_2025_1397_MOESM3_ESM.zip › 3.Manually_checked_genes/4.Trees/B-H1-1.pdf]

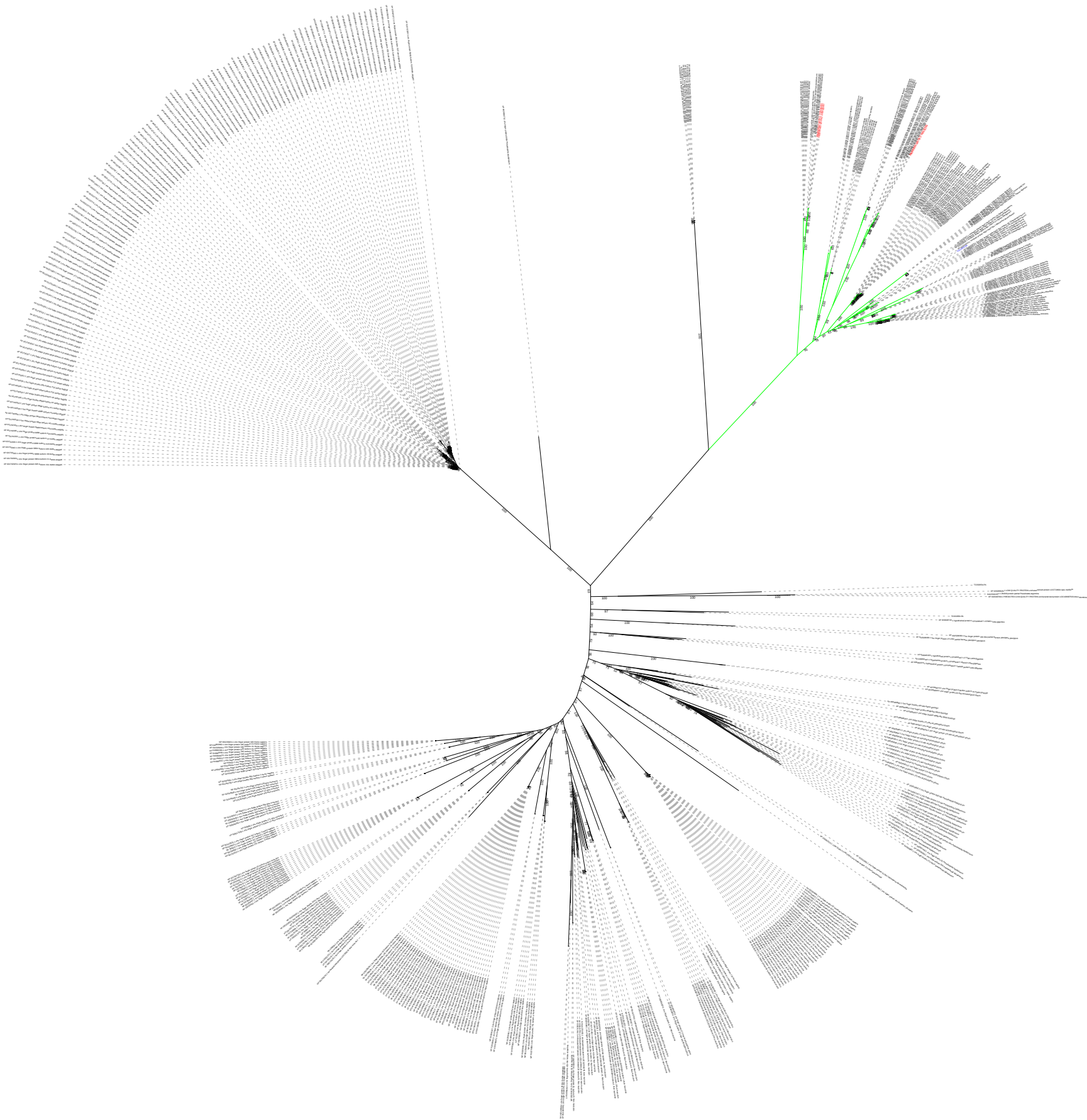

Supplement: Supplementary file 3 — Supplementary Material 3 [file 12863_2025_1397_MOESM3_ESM.zip › 3.Manually_checked_genes/4.Trees/salr_salm.pdf]

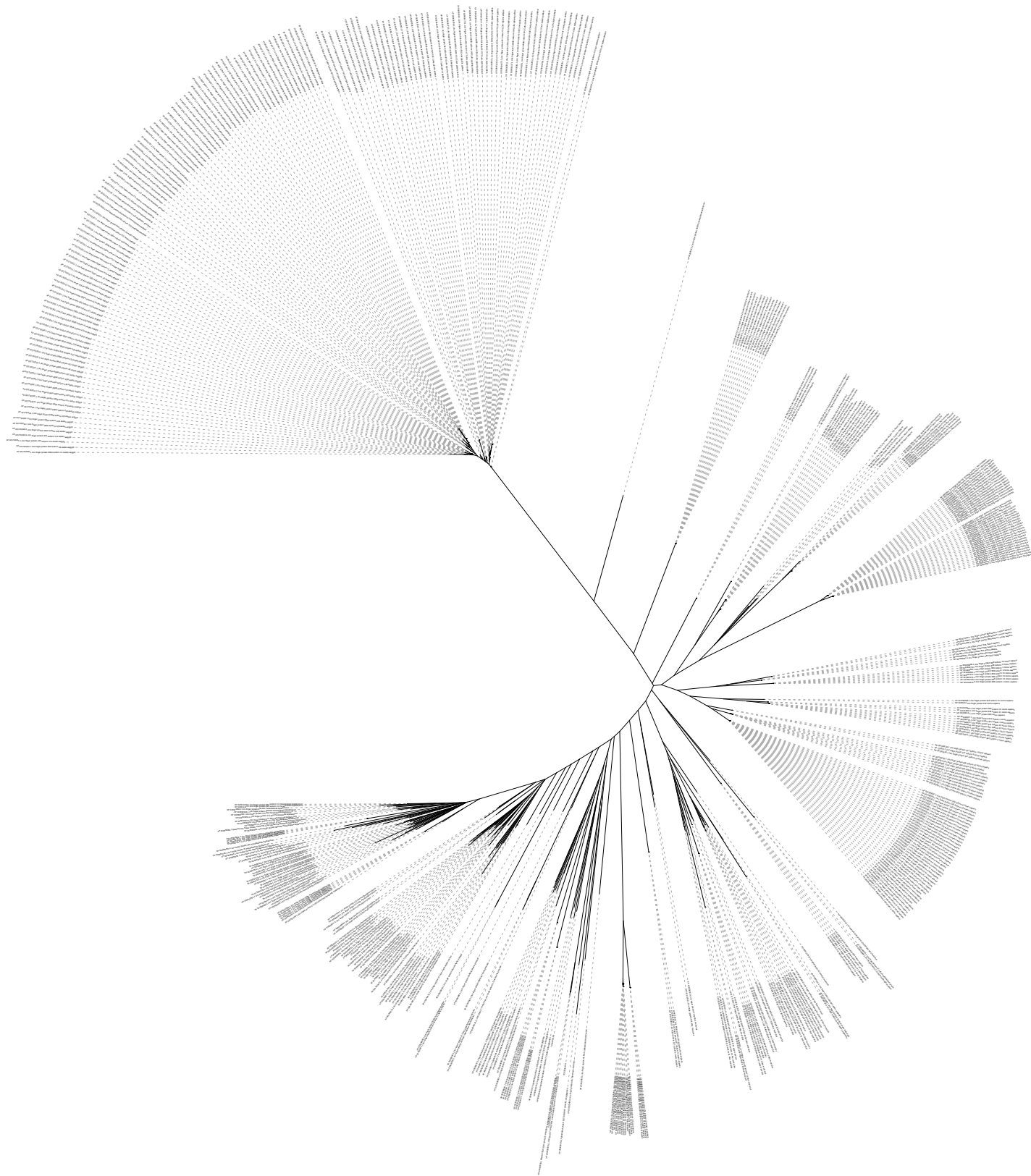

Supplement: Supplementary file 3 — Supplementary Material 3 [file 12863_2025_1397_MOESM3_ESM.zip › 3.Manually_checked_genes/4.Trees/Dll.pdf]

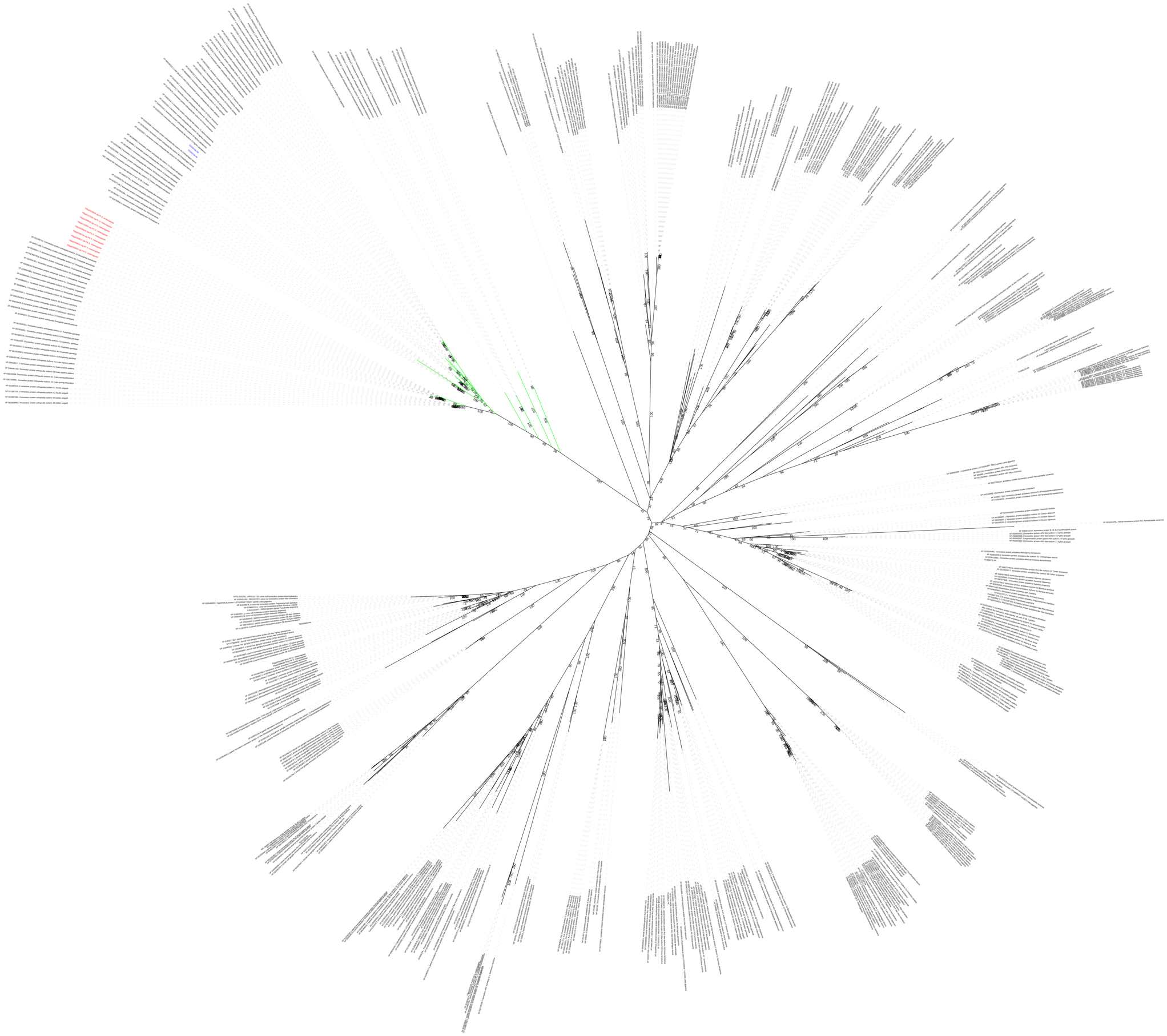

Supplement: Supplementary file 3 — Supplementary Material 3 [file 12863_2025_1397_MOESM3_ESM.zip › 3.Manually_checked_genes/4.Trees/otp.pdf]

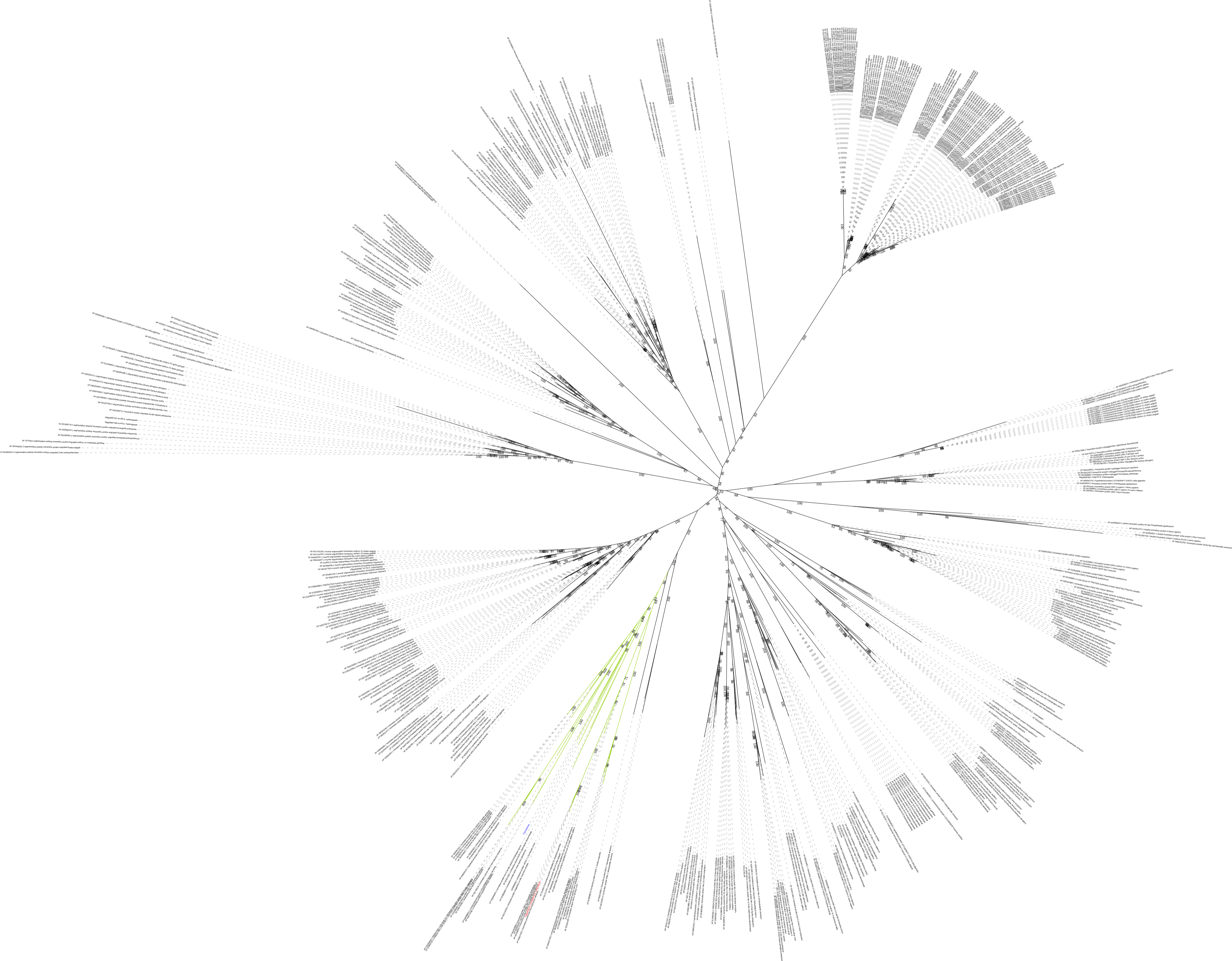

Supplement: Supplementary file 3 — Supplementary Material 3 [file 12863_2025_1397_MOESM3_ESM.zip › 3.Manually_checked_genes/4.Trees/CG15696.pdf]

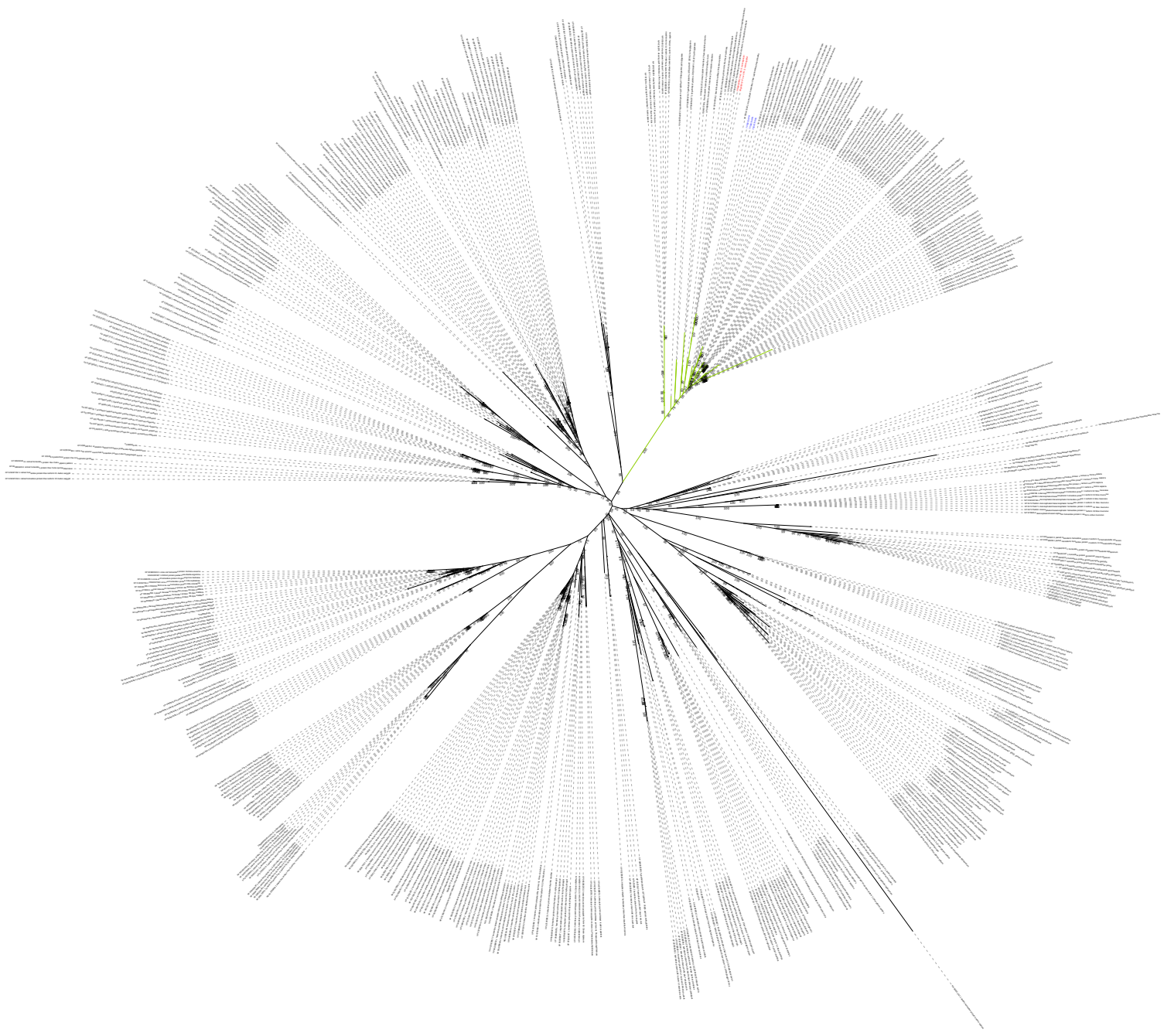

Supplement: Supplementary file 3 — Supplementary Material 3 [file 12863_2025_1397_MOESM3_ESM.zip › 3.Manually_checked_genes/4.Trees/Vsx1.pdf]

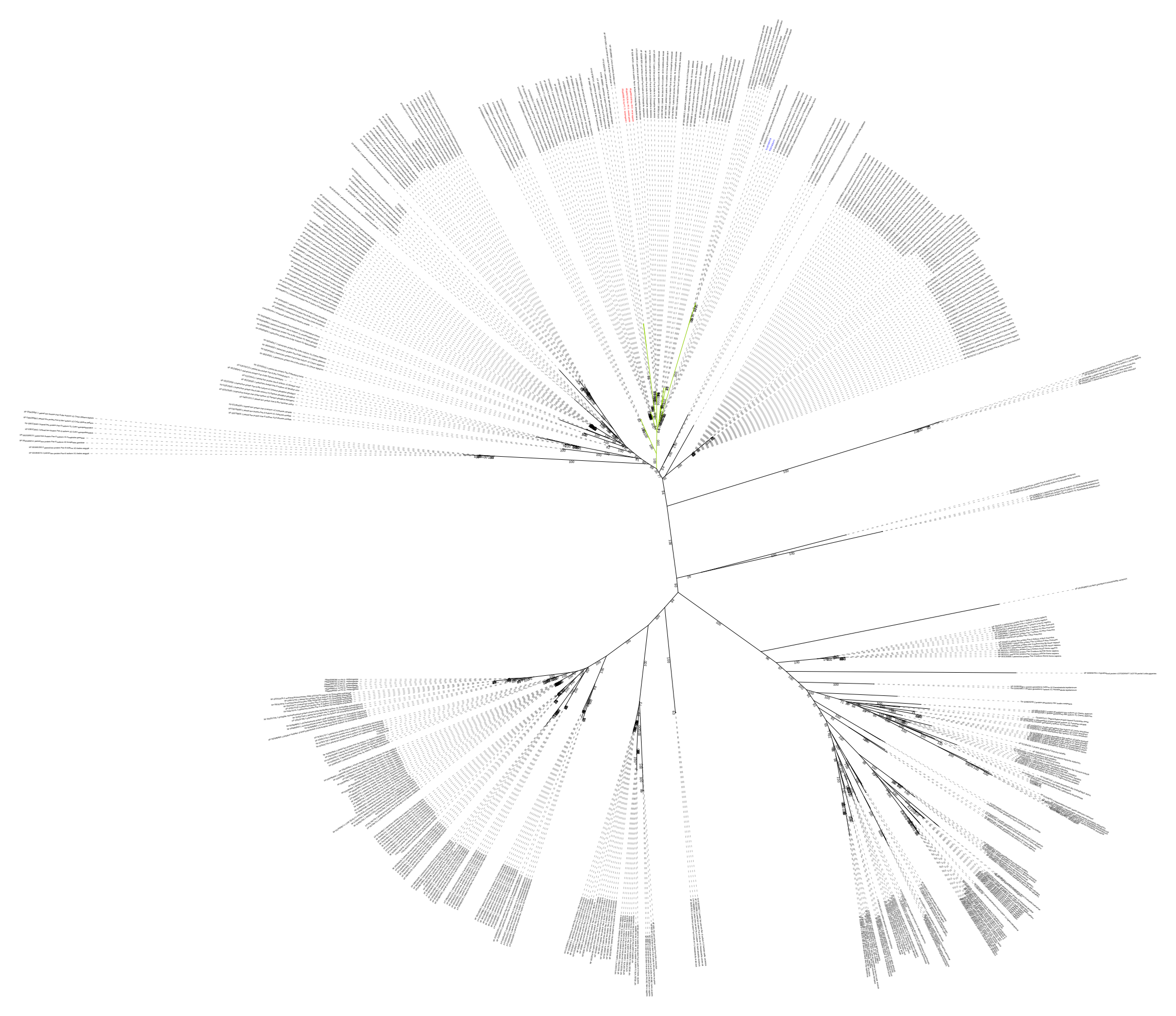

Supplement: Supplementary file 3 — Supplementary Material 3 [file 12863_2025_1397_MOESM3_ESM.zip › 3.Manually_checked_genes/4.Trees/toy.pdf]

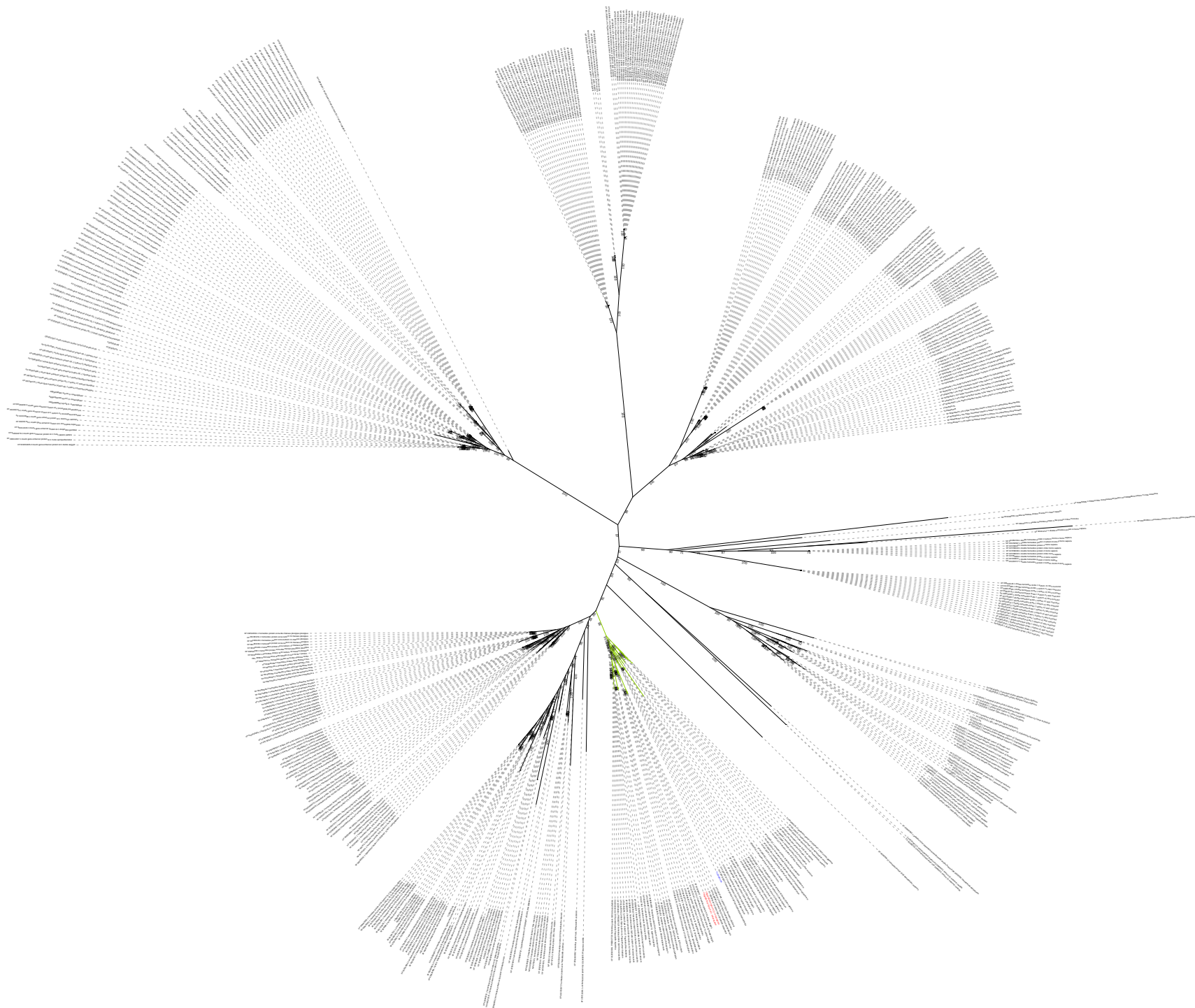

Supplement: Supplementary file 3 — Supplementary Material 3 [file 12863_2025_1397_MOESM3_ESM.zip › 3.Manually_checked_genes/4.Trees/Optix.pdf]

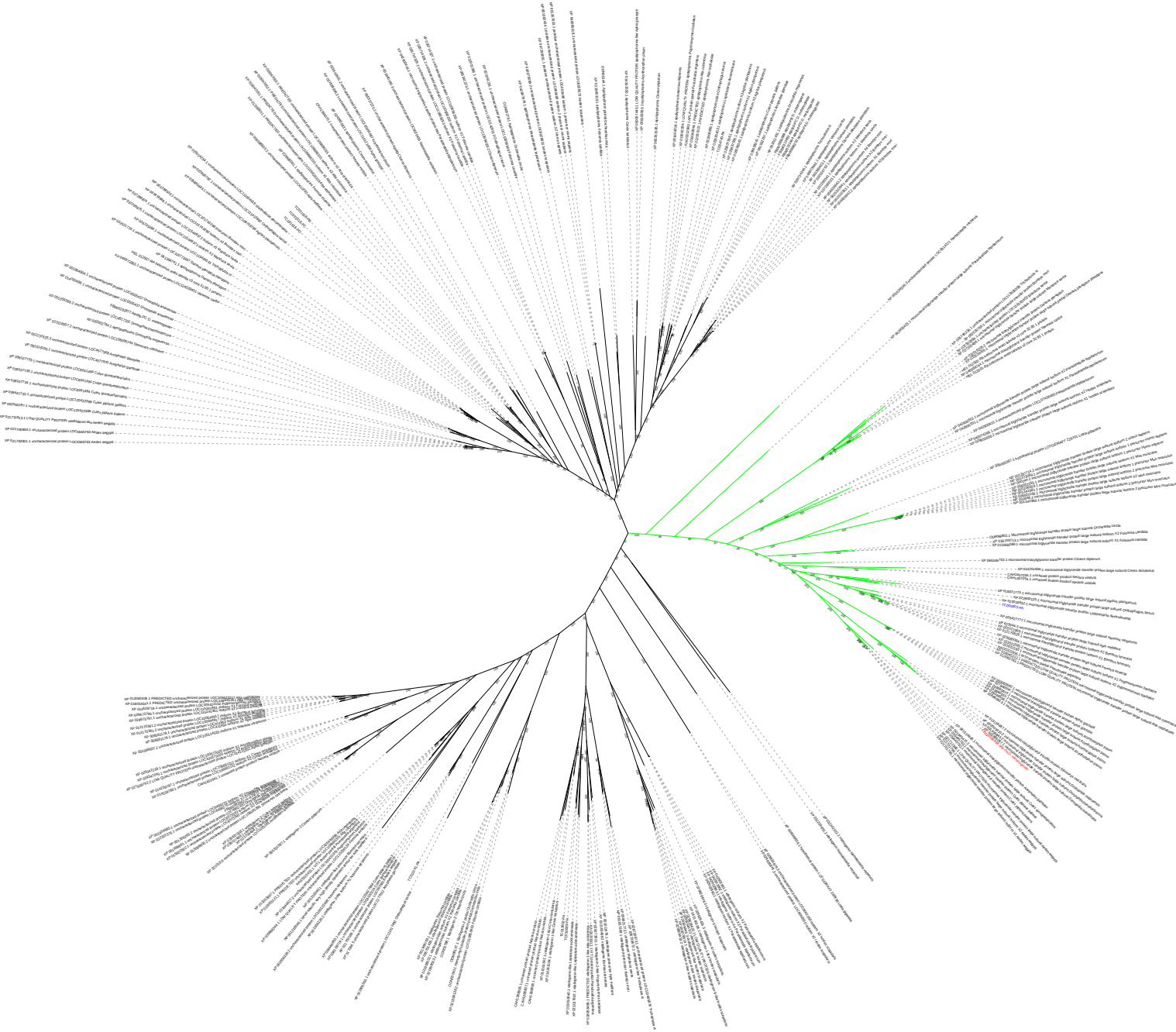

Supplement: Supplementary file 3 — Supplementary Material 3 [file 12863_2025_1397_MOESM3_ESM.zip › 3.Manually_checked_genes/4.Trees/Mtp.pdf]

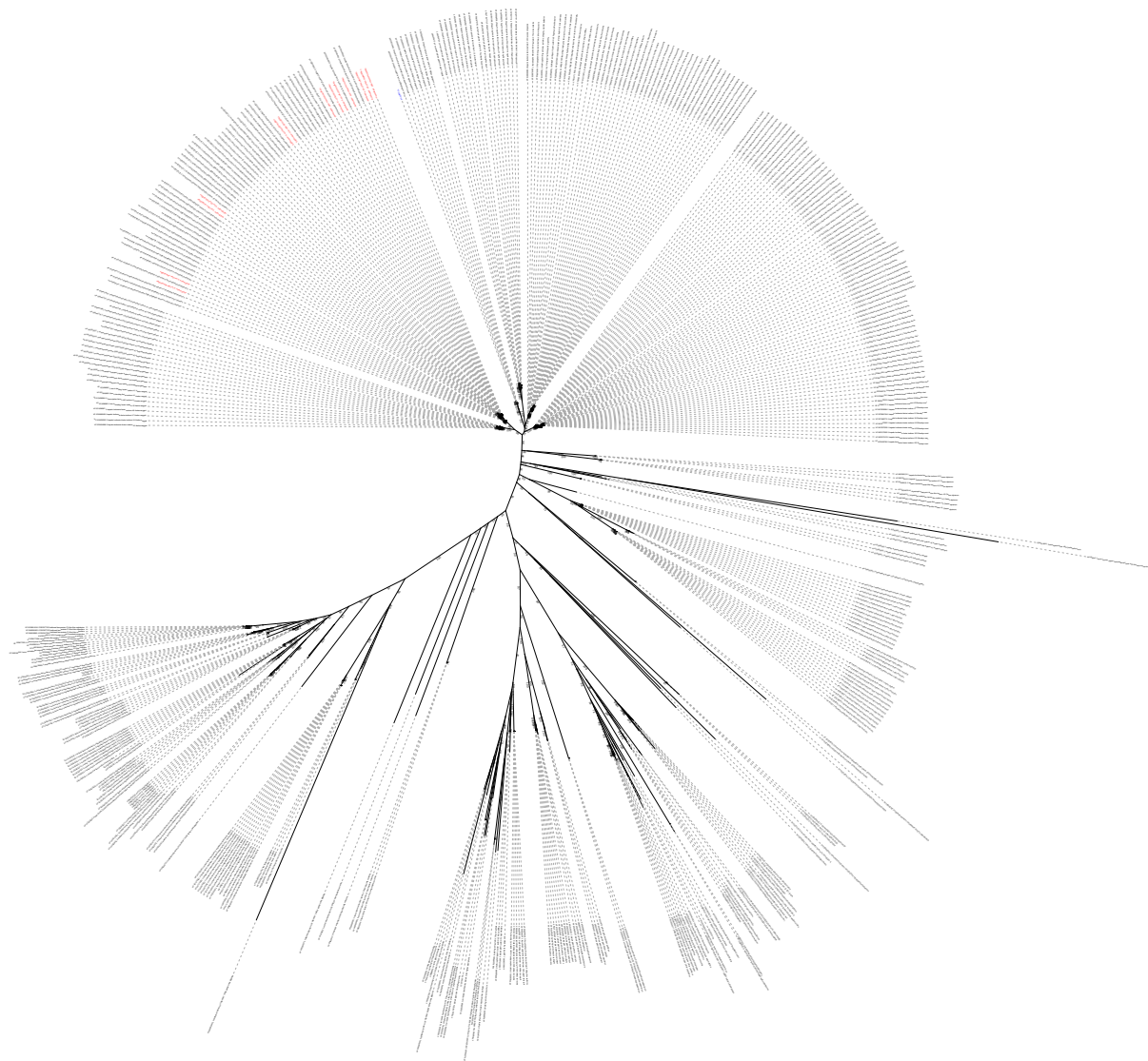

Supplement: Supplementary file 3 — Supplementary Material 3 [file 12863_2025_1397_MOESM3_ESM.zip › 3.Manually_checked_genes/4.Trees/Pdp1.pdf]

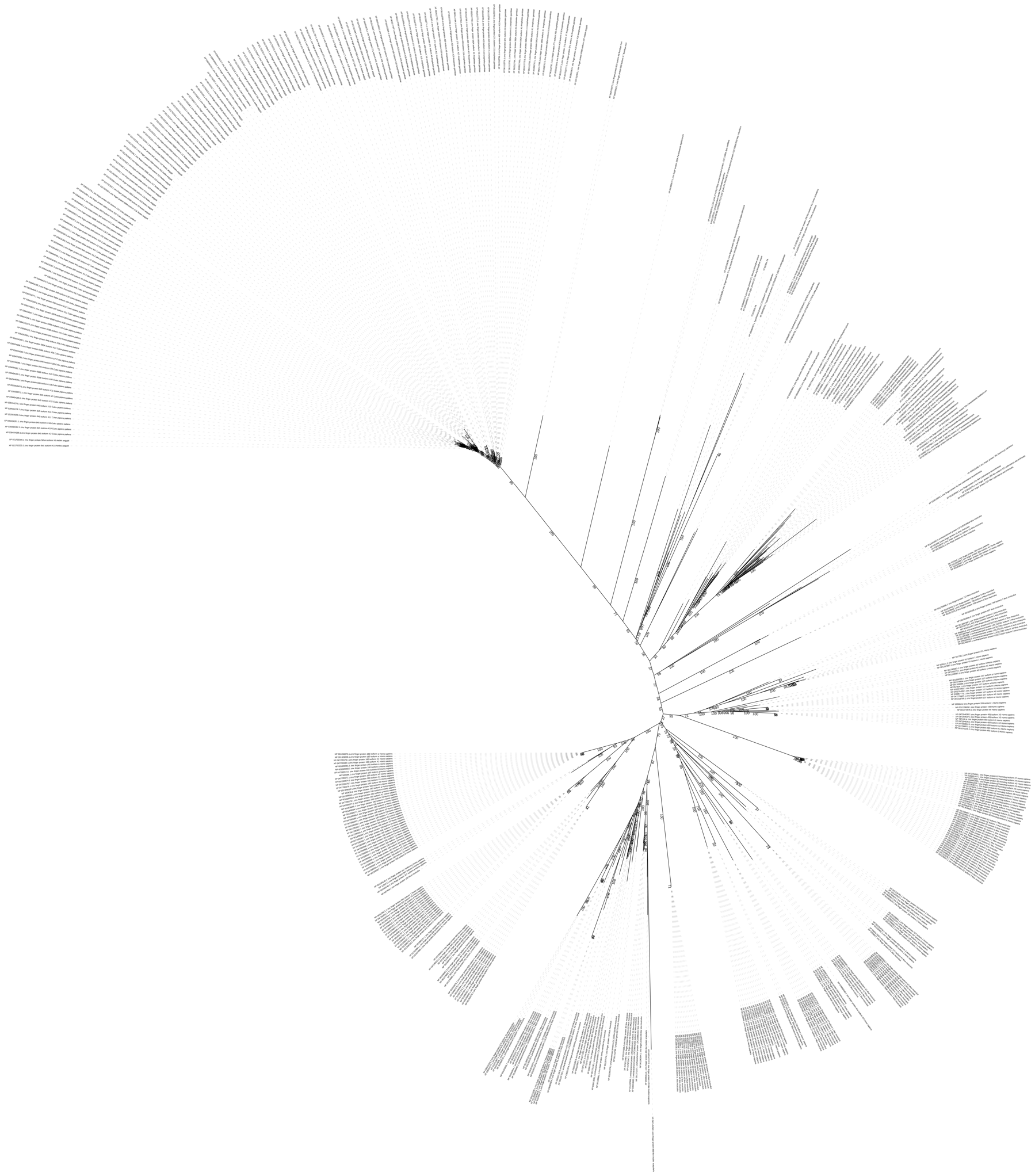

Supplement: Supplementary file 3 — Supplementary Material 3 [file 12863_2025_1397_MOESM3_ESM.zip › 3.Manually_checked_genes/4.Trees/mld.pdf]

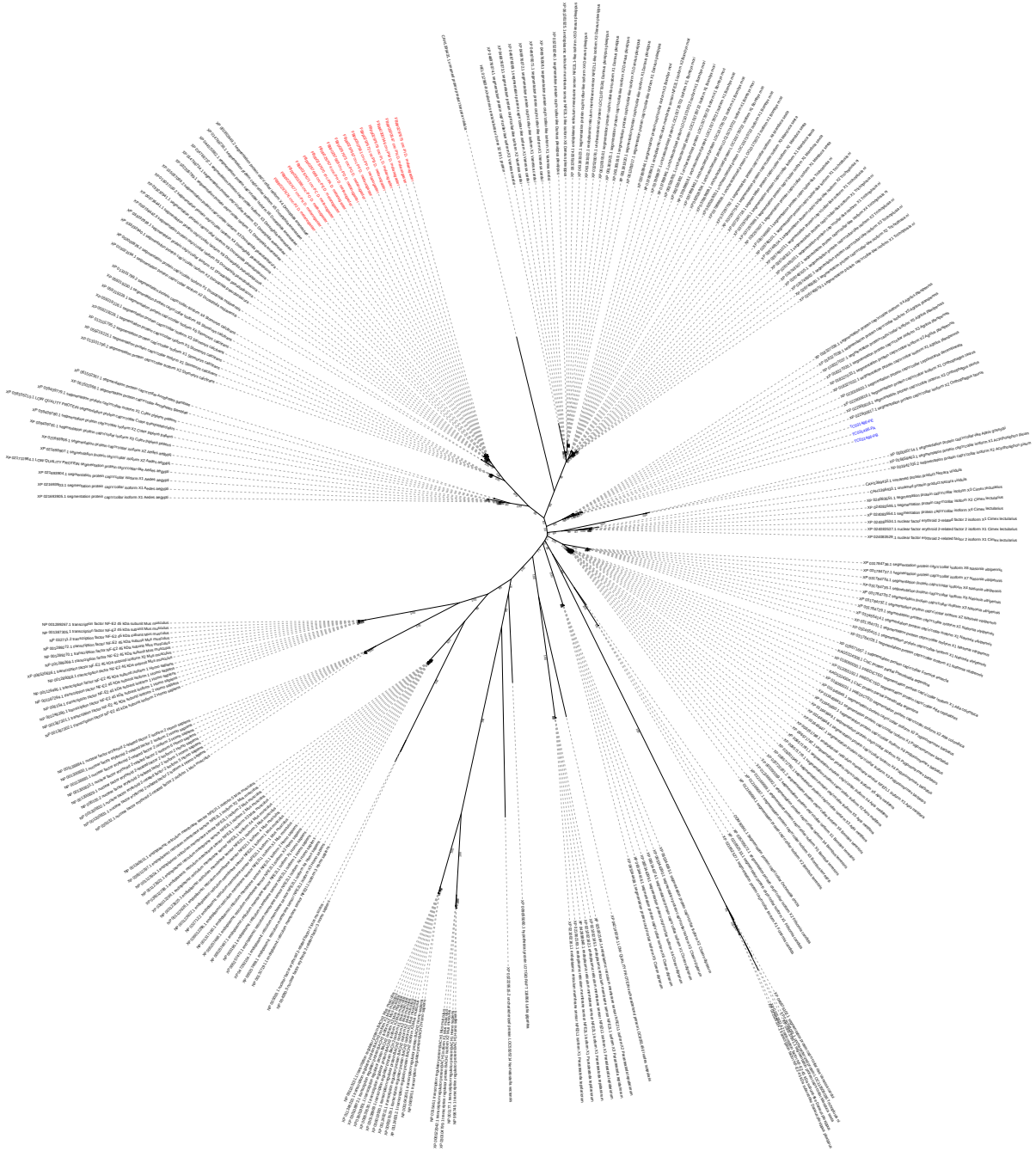

Supplement: Supplementary file 3 — Supplementary Material 3 [file 12863_2025_1397_MOESM3_ESM.zip › 3.Manually_checked_genes/4.Trees/cnc.pdf]

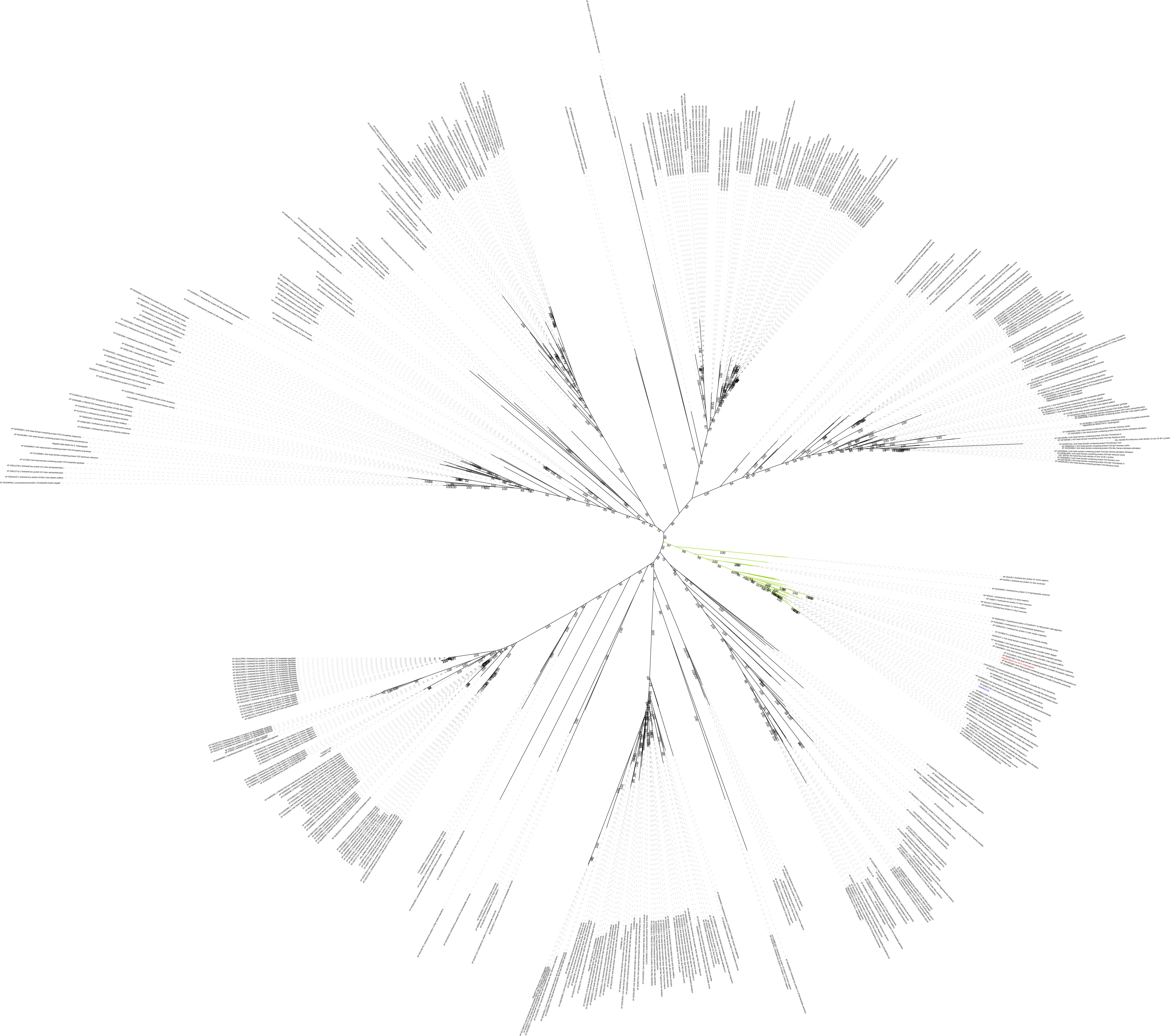

Supplement: Supplementary file 3 — Supplementary Material 3 [file 12863_2025_1397_MOESM3_ESM.zip › 3.Manually_checked_genes/4.Trees/croc.pdf]

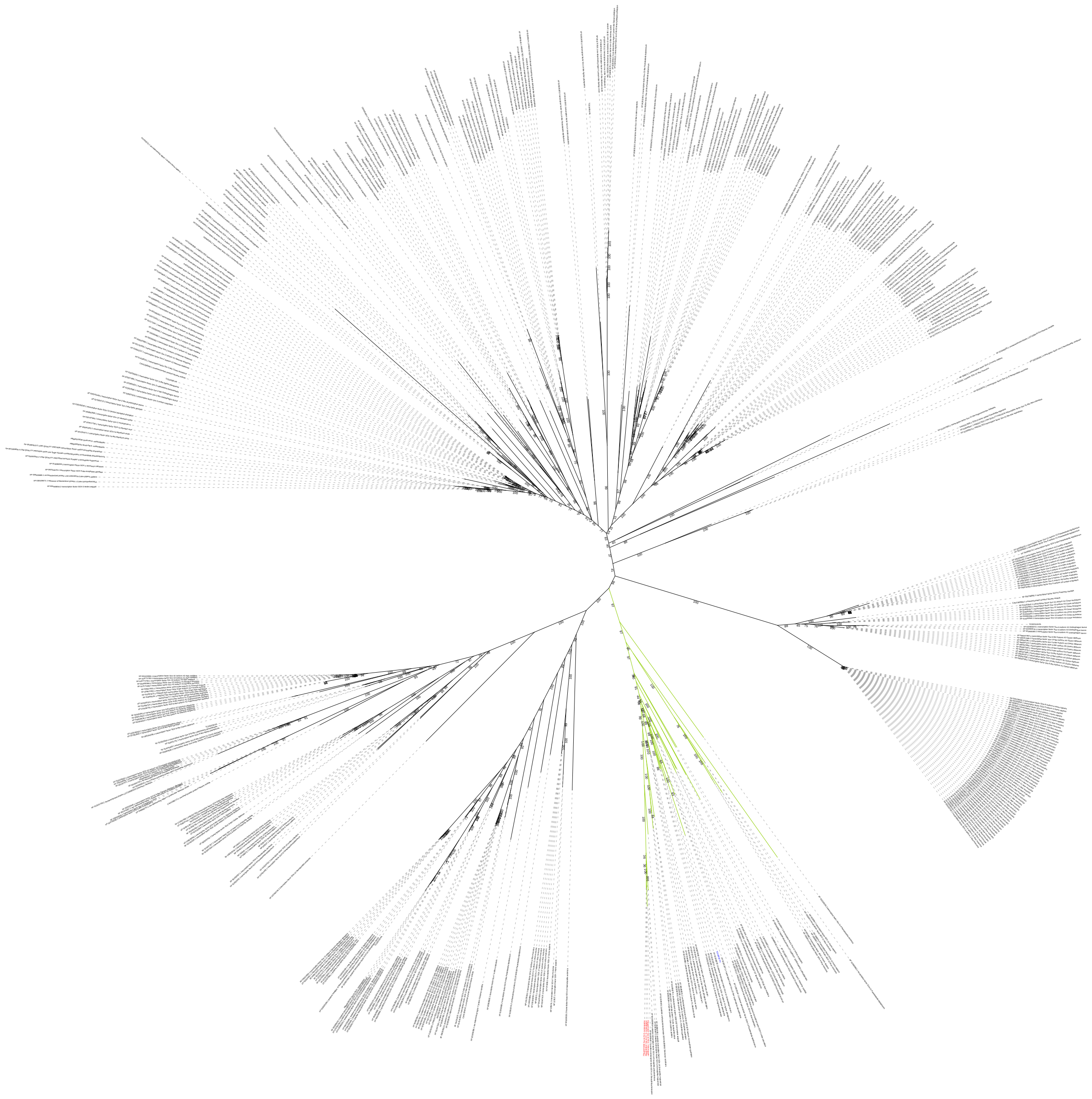

Supplement: Supplementary file 3 — Supplementary Material 3 [file 12863_2025_1397_MOESM3_ESM.zip › 3.Manually_checked_genes/4.Trees/Sox14.pdf]

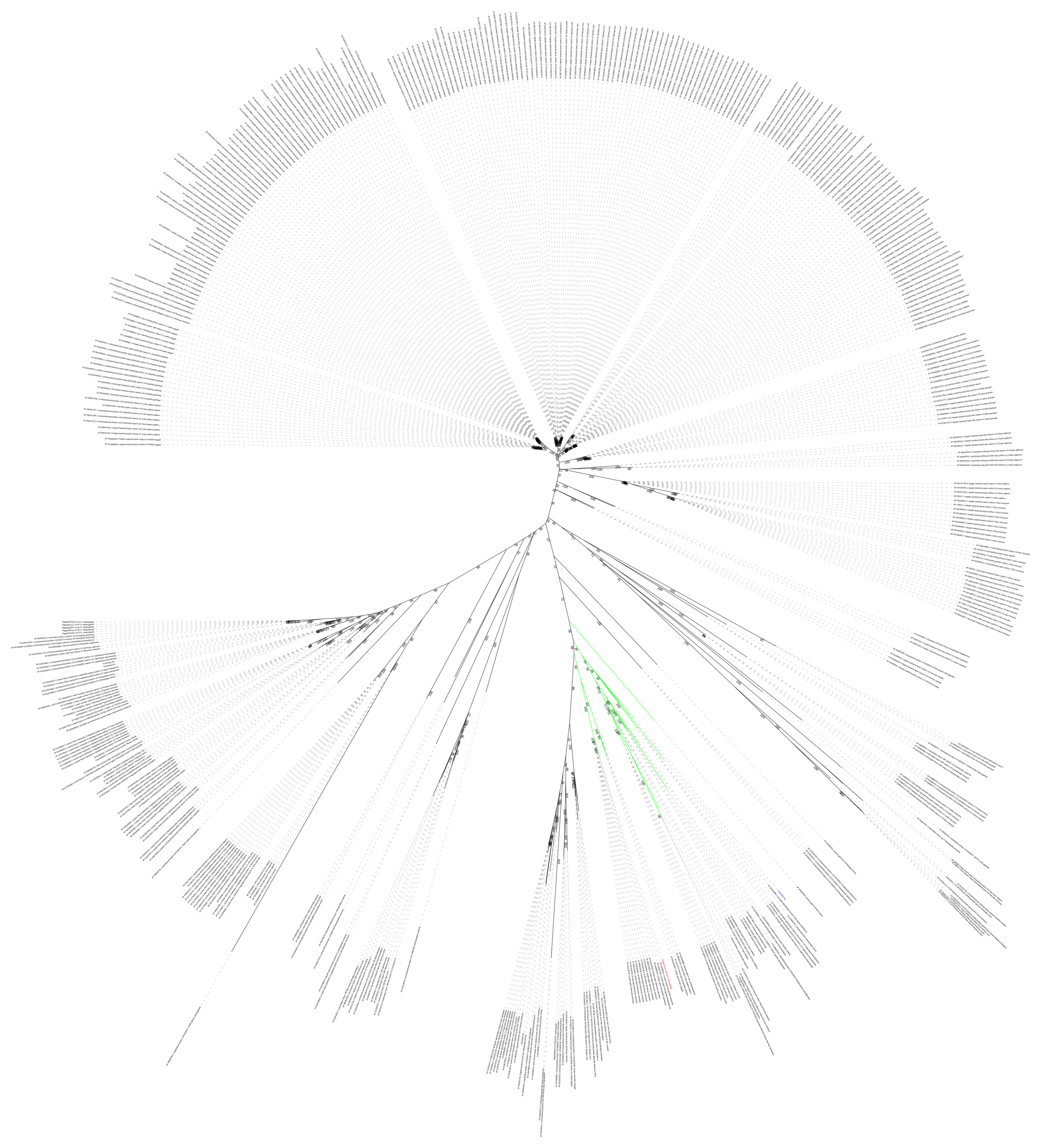

Supplement: Supplementary file 3 — Supplementary Material 3 [file 12863_2025_1397_MOESM3_ESM.zip › 3.Manually_checked_genes/4.Trees/gt.pdf]

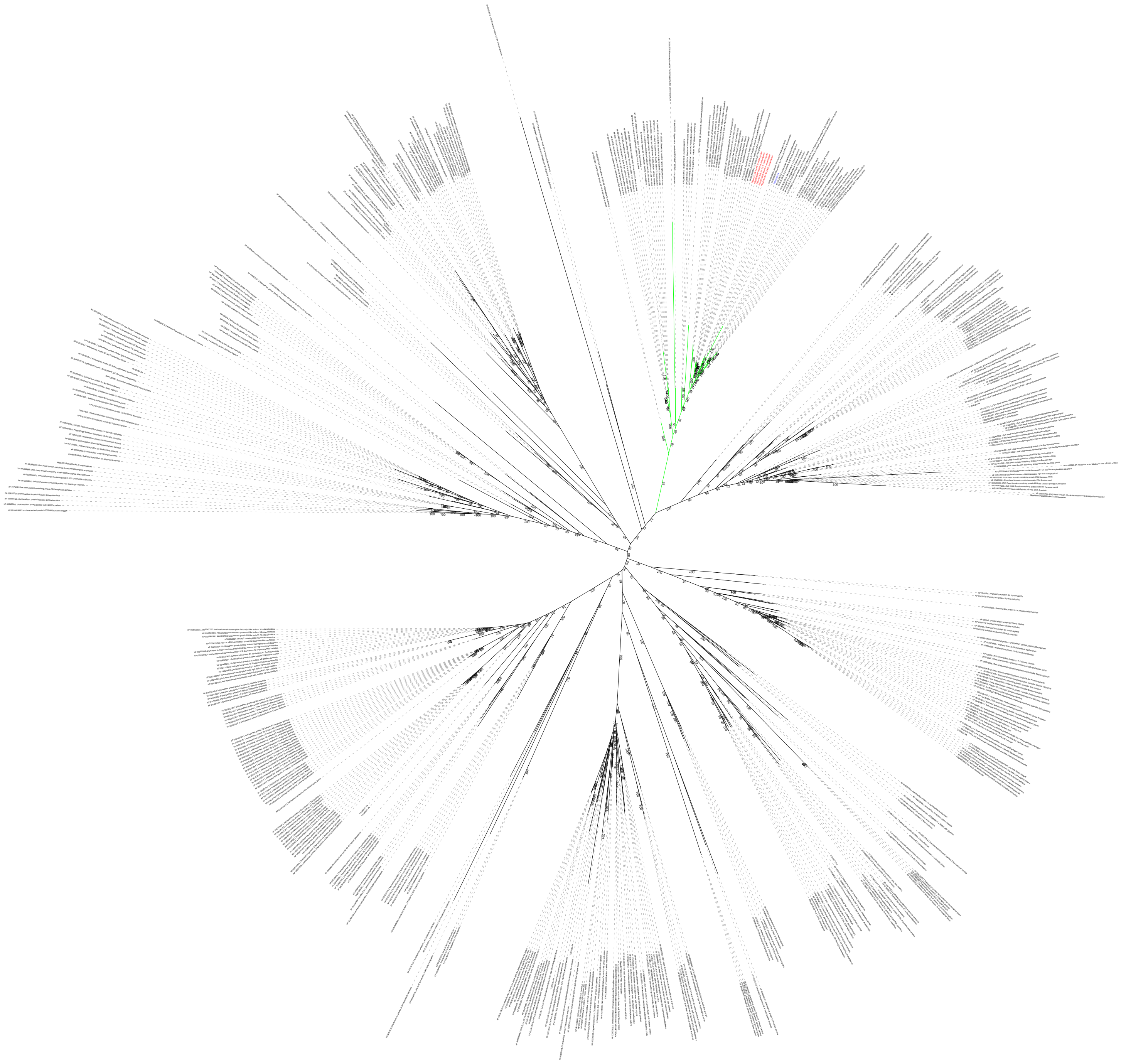

Supplement: Supplementary file 3 — Supplementary Material 3 [file 12863_2025_1397_MOESM3_ESM.zip › 3.Manually_checked_genes/4.Trees/fkh.pdf]

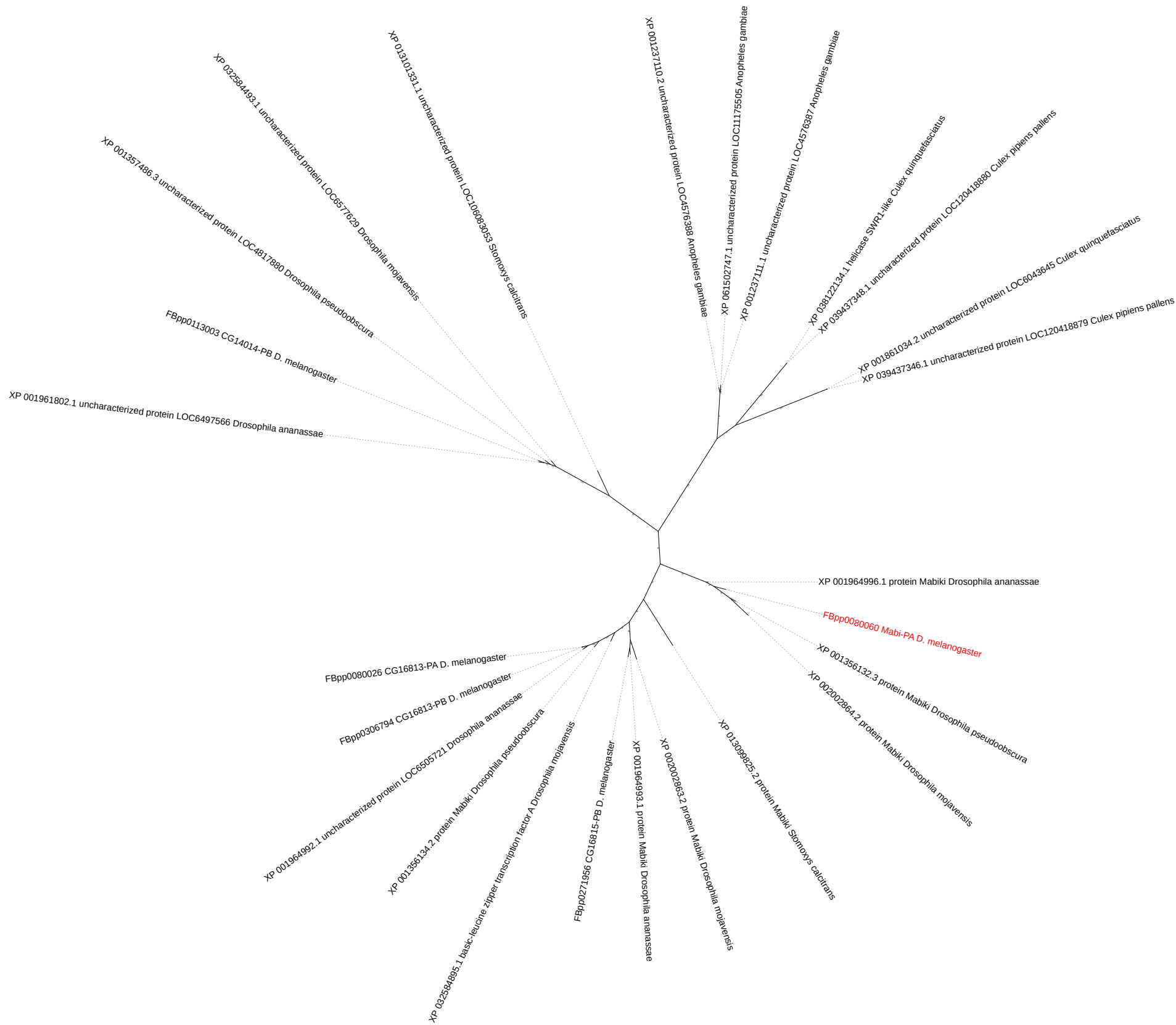

Supplement: Supplementary file 3 — Supplementary Material 3 [file 12863_2025_1397_MOESM3_ESM.zip › 3.Manually_checked_genes/4.Trees/Mabi.pdf]

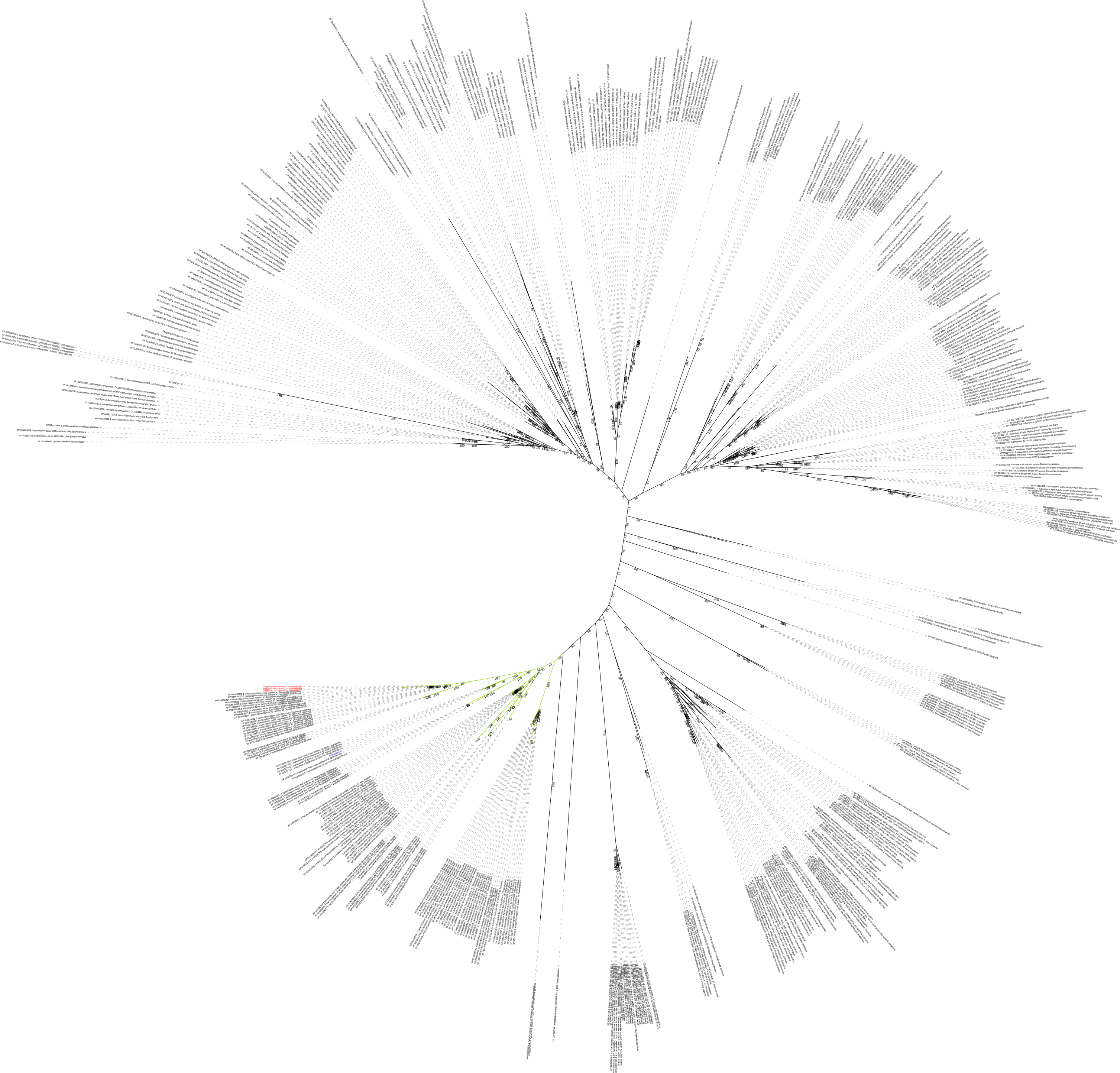

Supplement: Supplementary file 3 — Supplementary Material 3 [file 12863_2025_1397_MOESM3_ESM.zip › 3.Manually_checked_genes/4.Trees/cwo.pdf]

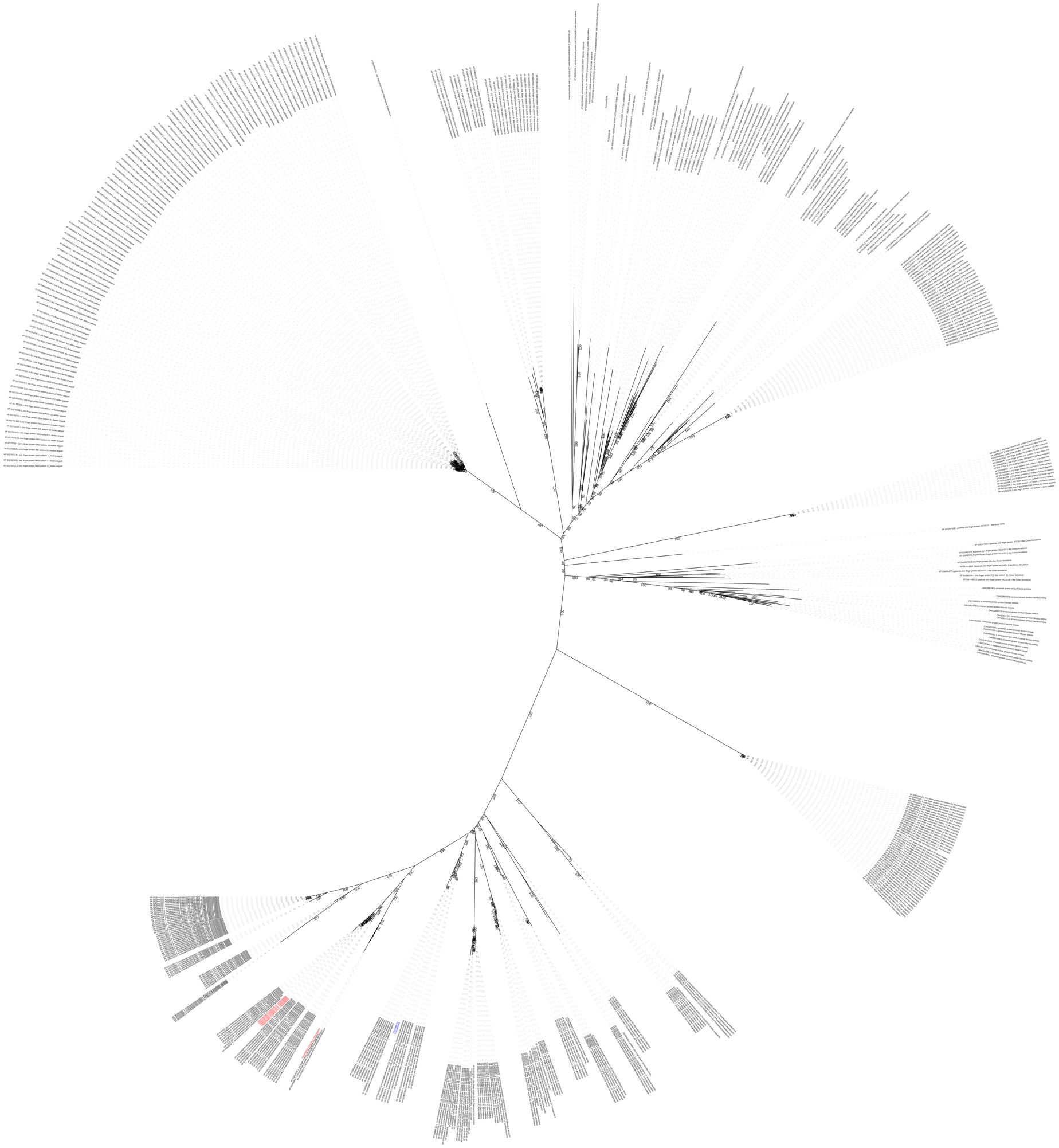

Supplement: Supplementary file 3 — Supplementary Material 3 [file 12863_2025_1397_MOESM3_ESM.zip › 3.Manually_checked_genes/4.Trees/CG9932.pdf]

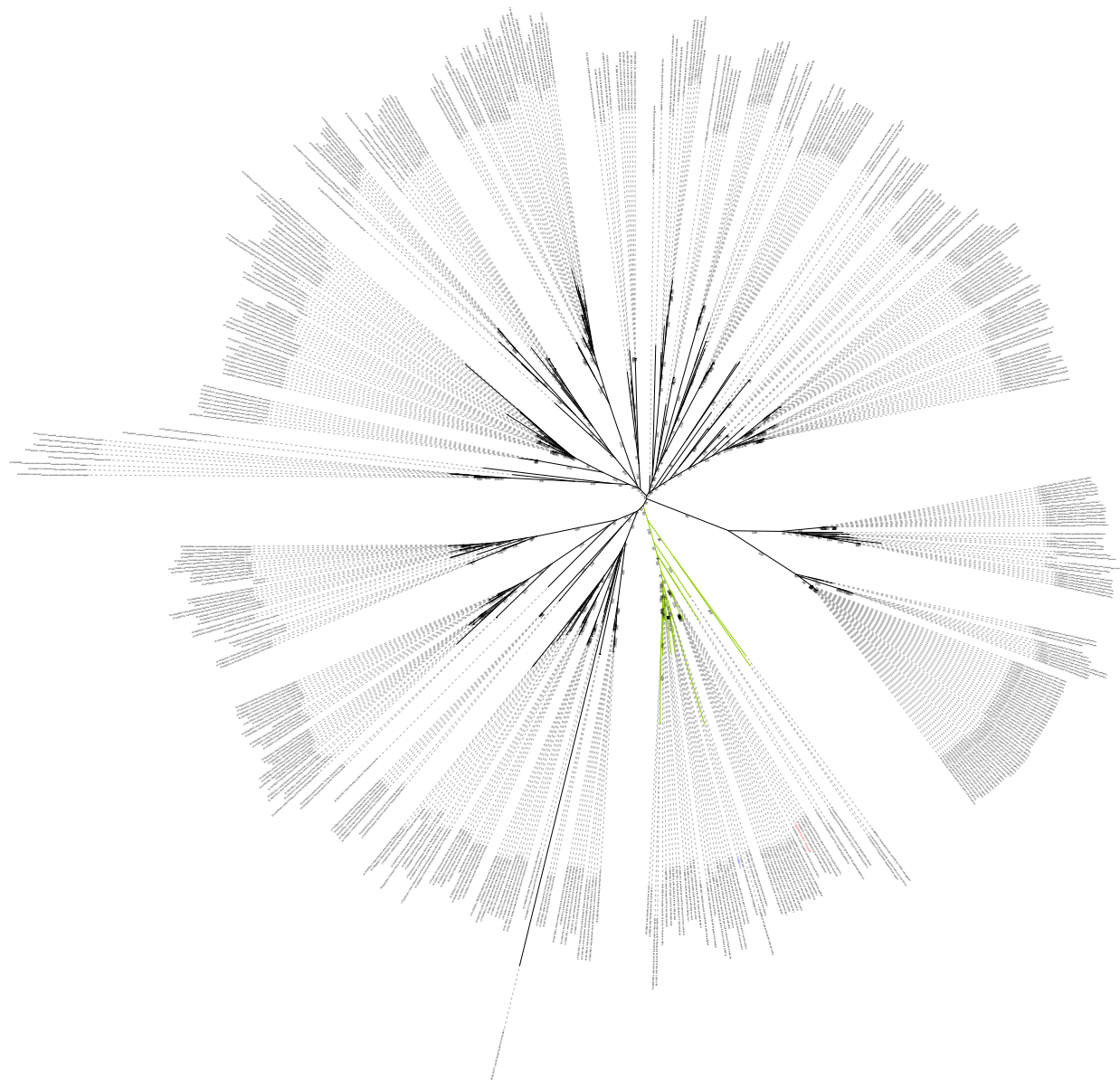

Supplement: Supplementary file 3 — Supplementary Material 3 [file 12863_2025_1397_MOESM3_ESM.zip › 3.Manually_checked_genes/4.Trees/Rx.pdf]

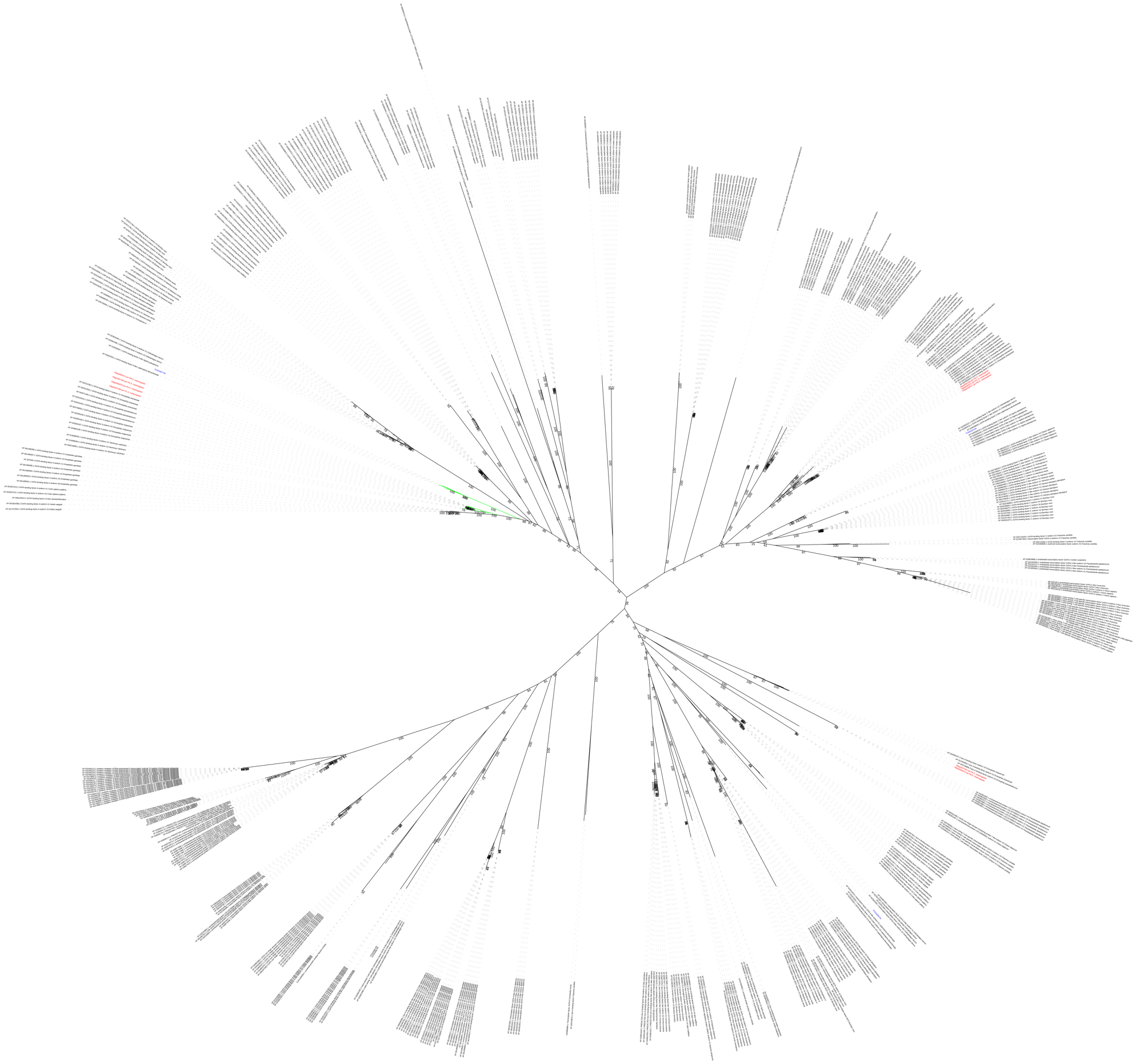

Supplement: Supplementary file 3 — Supplementary Material 3 [file 12863_2025_1397_MOESM3_ESM.zip › 3.Manually_checked_genes/4.Trees/pnr.pdf]

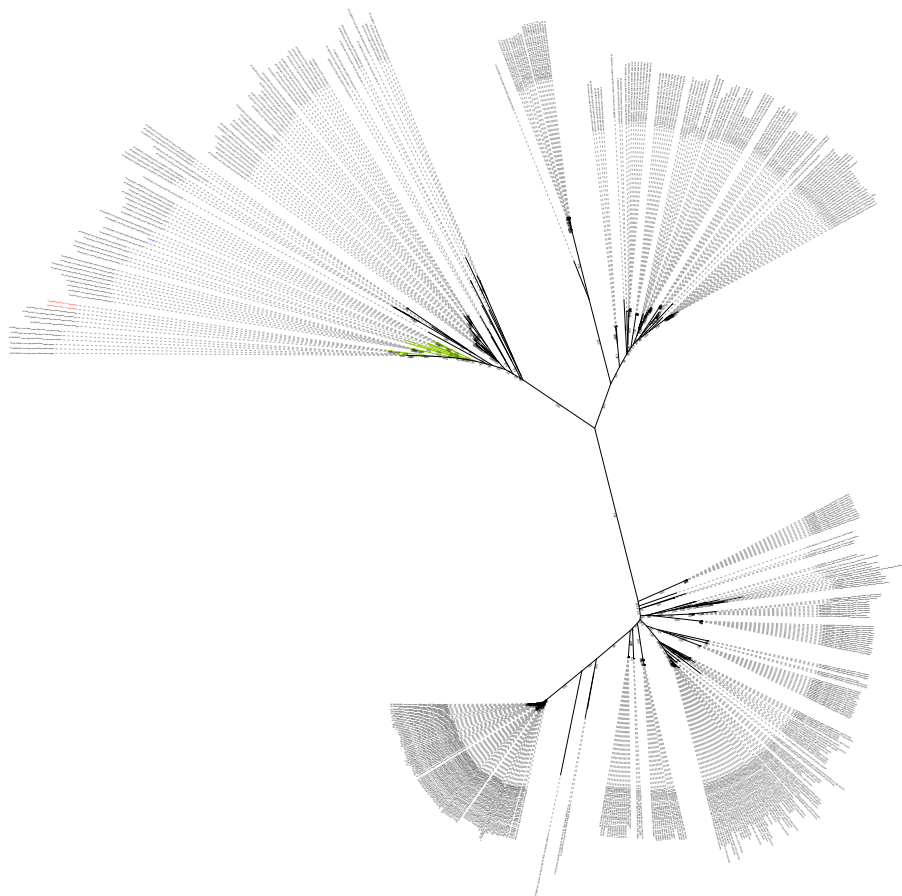

Supplement: Supplementary file 3 — Supplementary Material 3 [file 12863_2025_1397_MOESM3_ESM.zip › 3.Manually_checked_genes/4.Trees/elB.pdf]

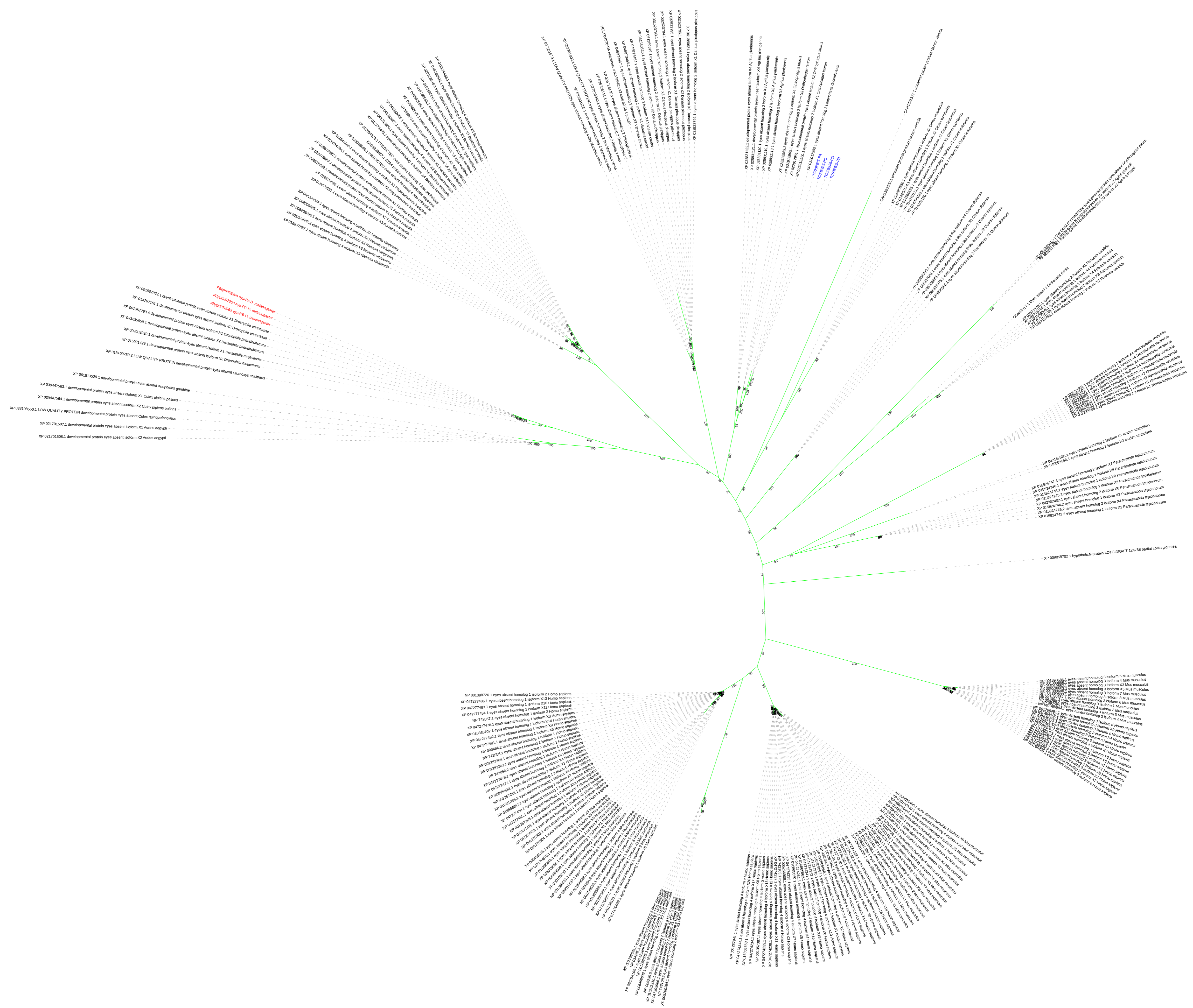

Supplement: Supplementary file 3 — Supplementary Material 3 [file 12863_2025_1397_MOESM3_ESM.zip › 3.Manually_checked_genes/4.Trees/eya.pdf]

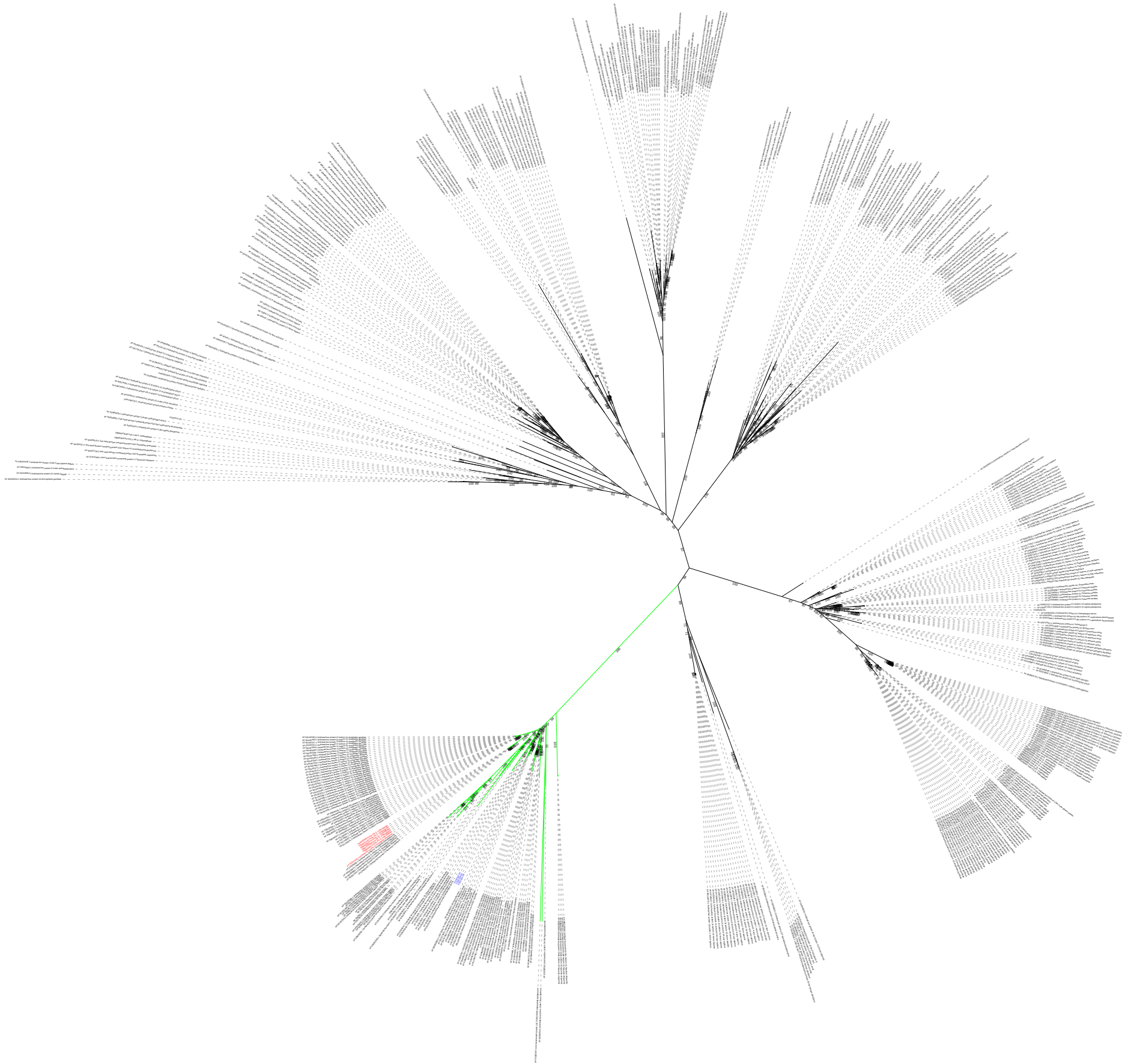

Supplement: Supplementary file 3 — Supplementary Material 3 [file 12863_2025_1397_MOESM3_ESM.zip › 3.Manually_checked_genes/4.Trees/foxo.pdf]

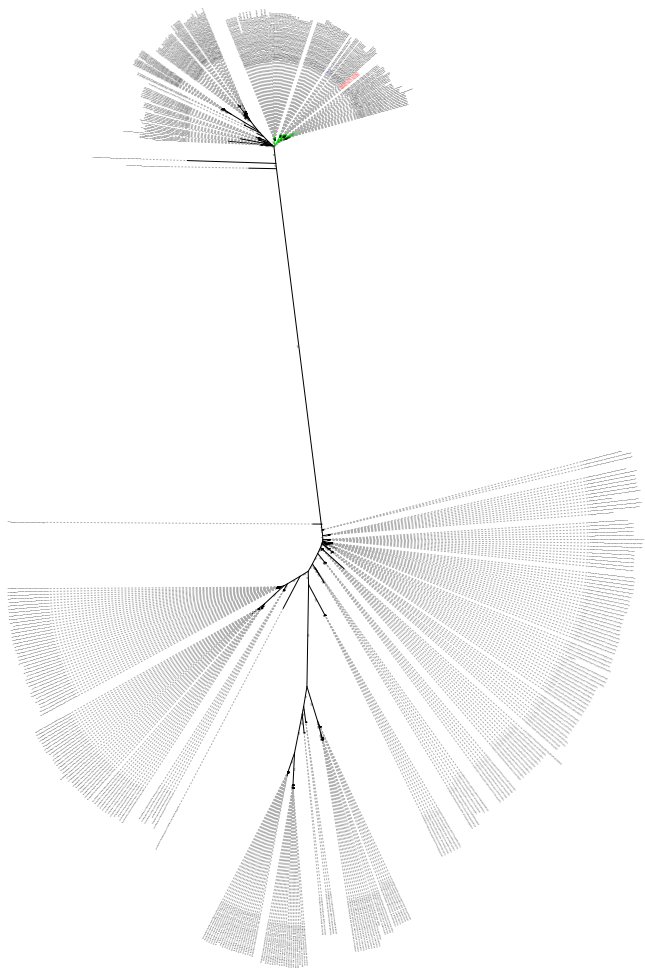

Supplement: Supplementary file 3 — Supplementary Material 3 [file 12863_2025_1397_MOESM3_ESM.zip › 3.Manually_checked_genes/4.Trees/exd.pdf]

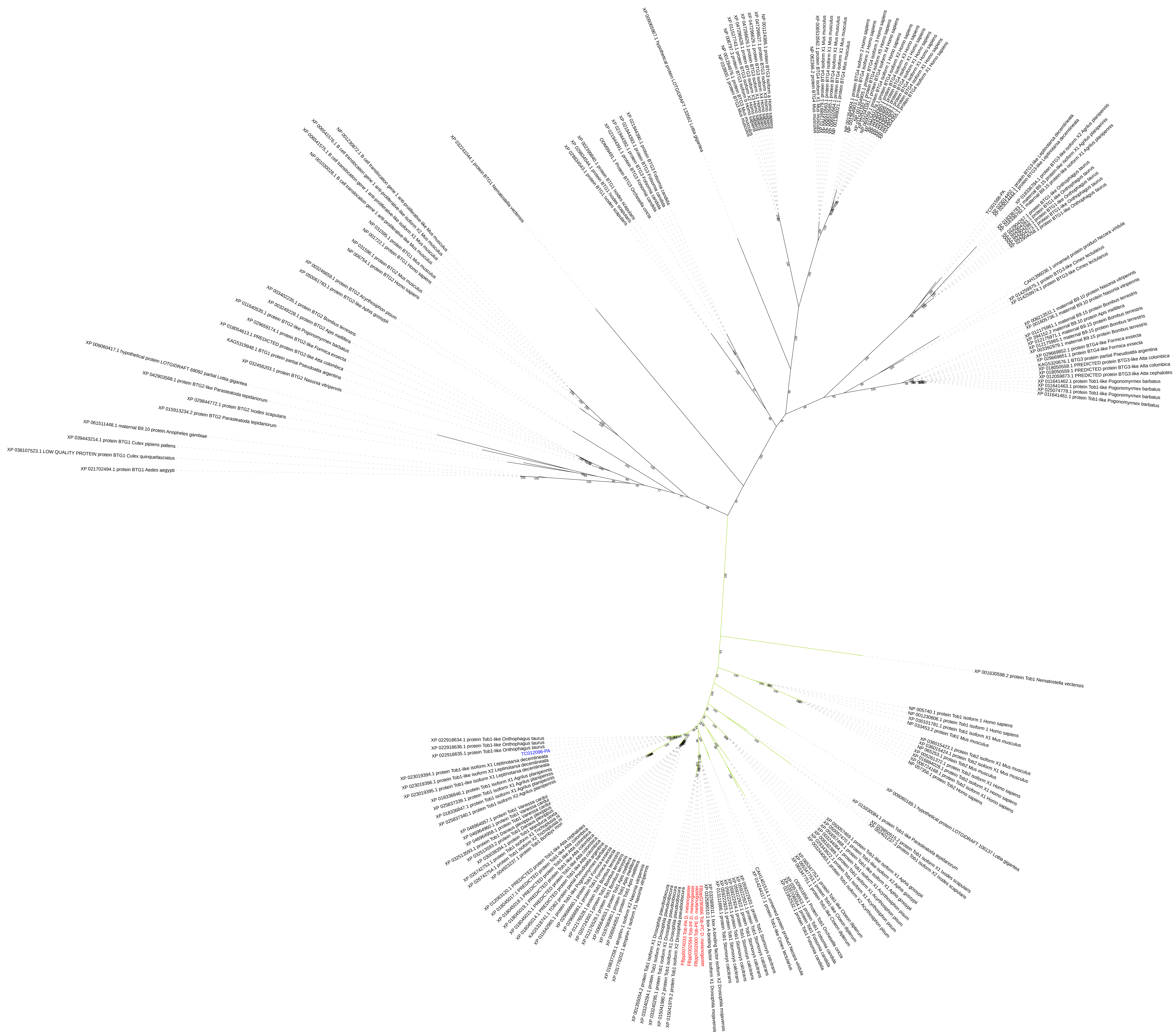

Supplement: Supplementary file 3 — Supplementary Material 3 [file 12863_2025_1397_MOESM3_ESM.zip › 3.Manually_checked_genes/4.Trees/Tob.pdf]

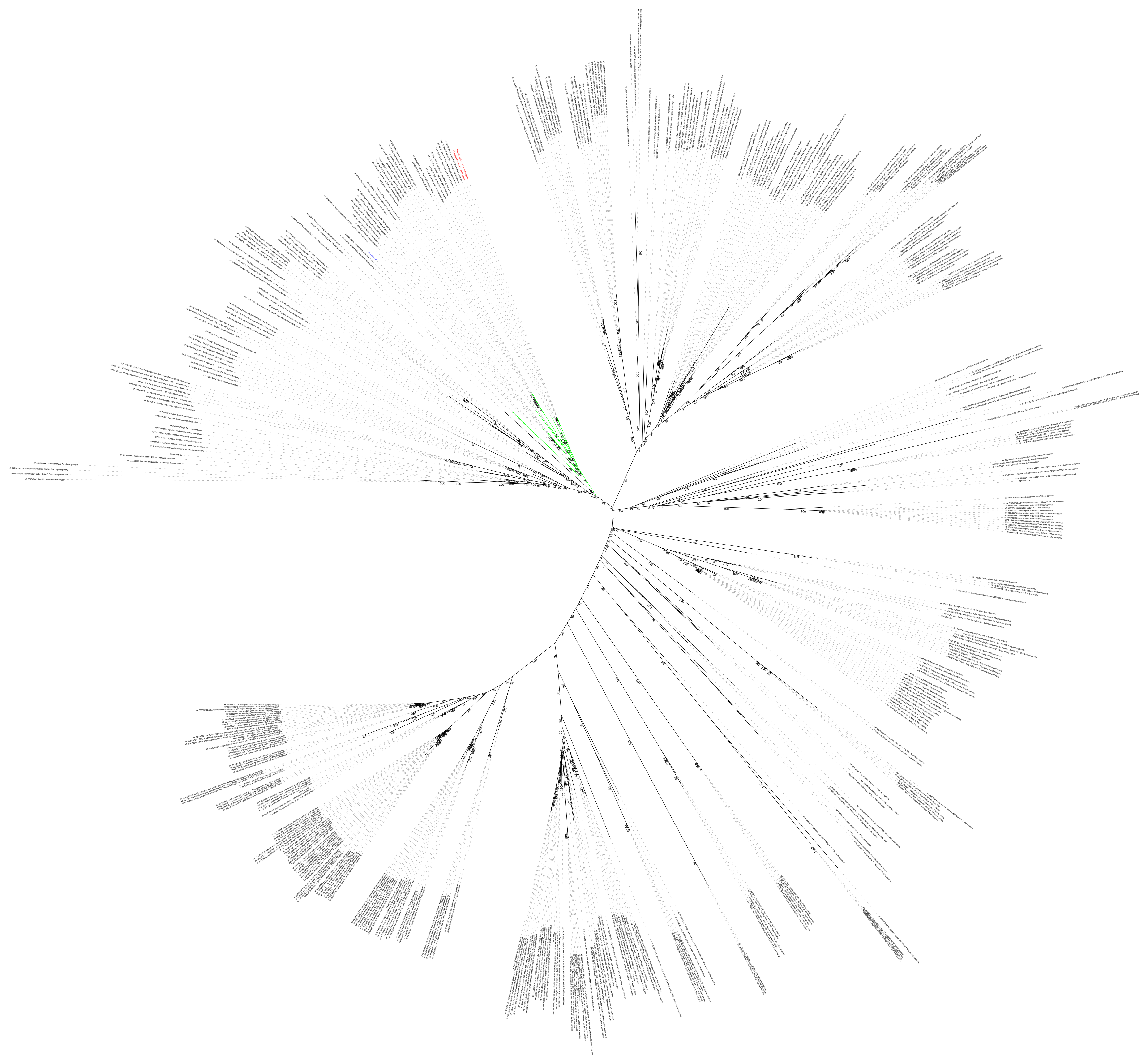

Supplement: Supplementary file 3 — Supplementary Material 3 [file 12863_2025_1397_MOESM3_ESM.zip › 3.Manually_checked_genes/4.Trees/hry.pdf]

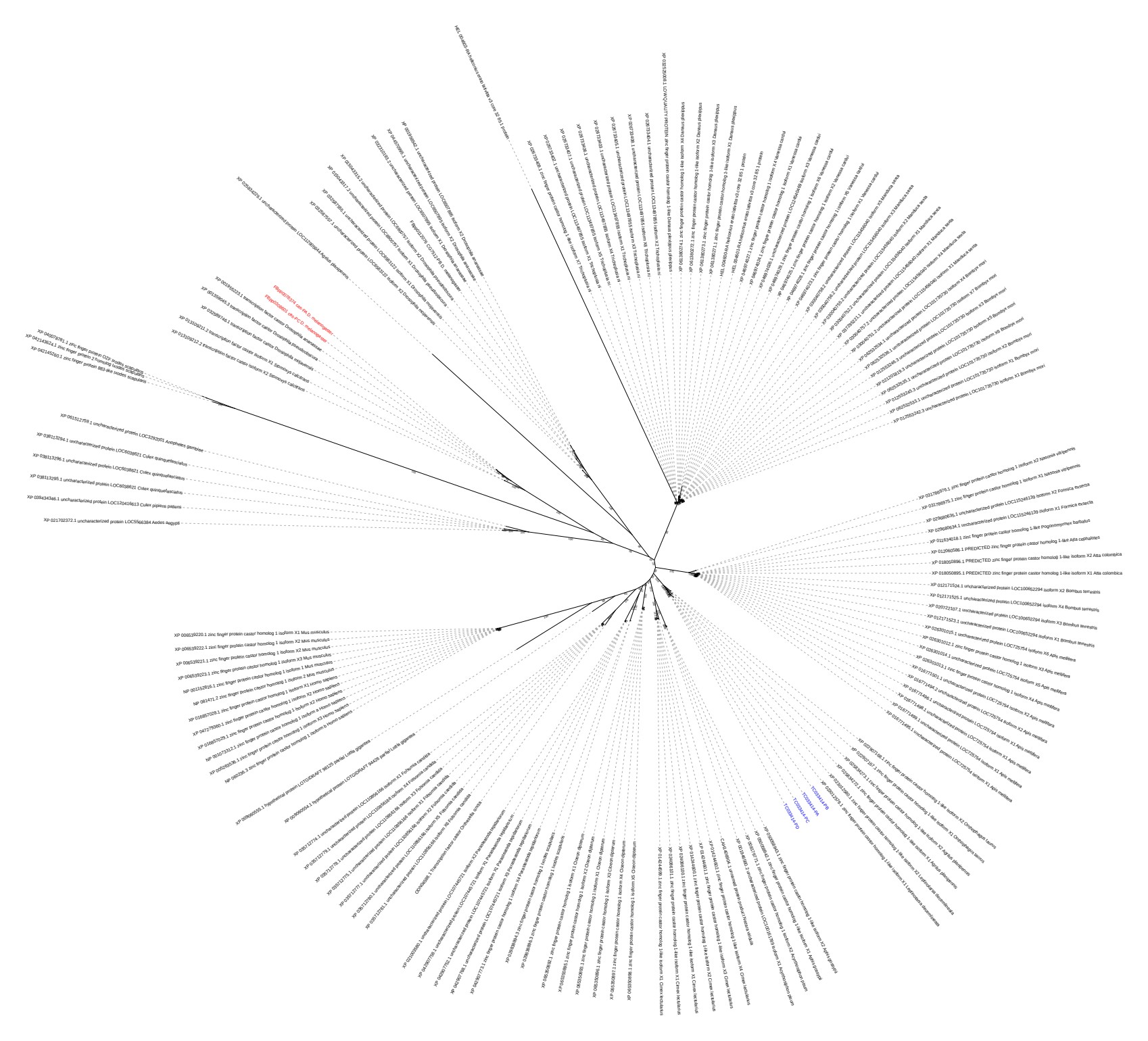

Supplement: Supplementary file 3 — Supplementary Material 3 [file 12863_2025_1397_MOESM3_ESM.zip › 3.Manually_checked_genes/4.Trees/cas.pdf]

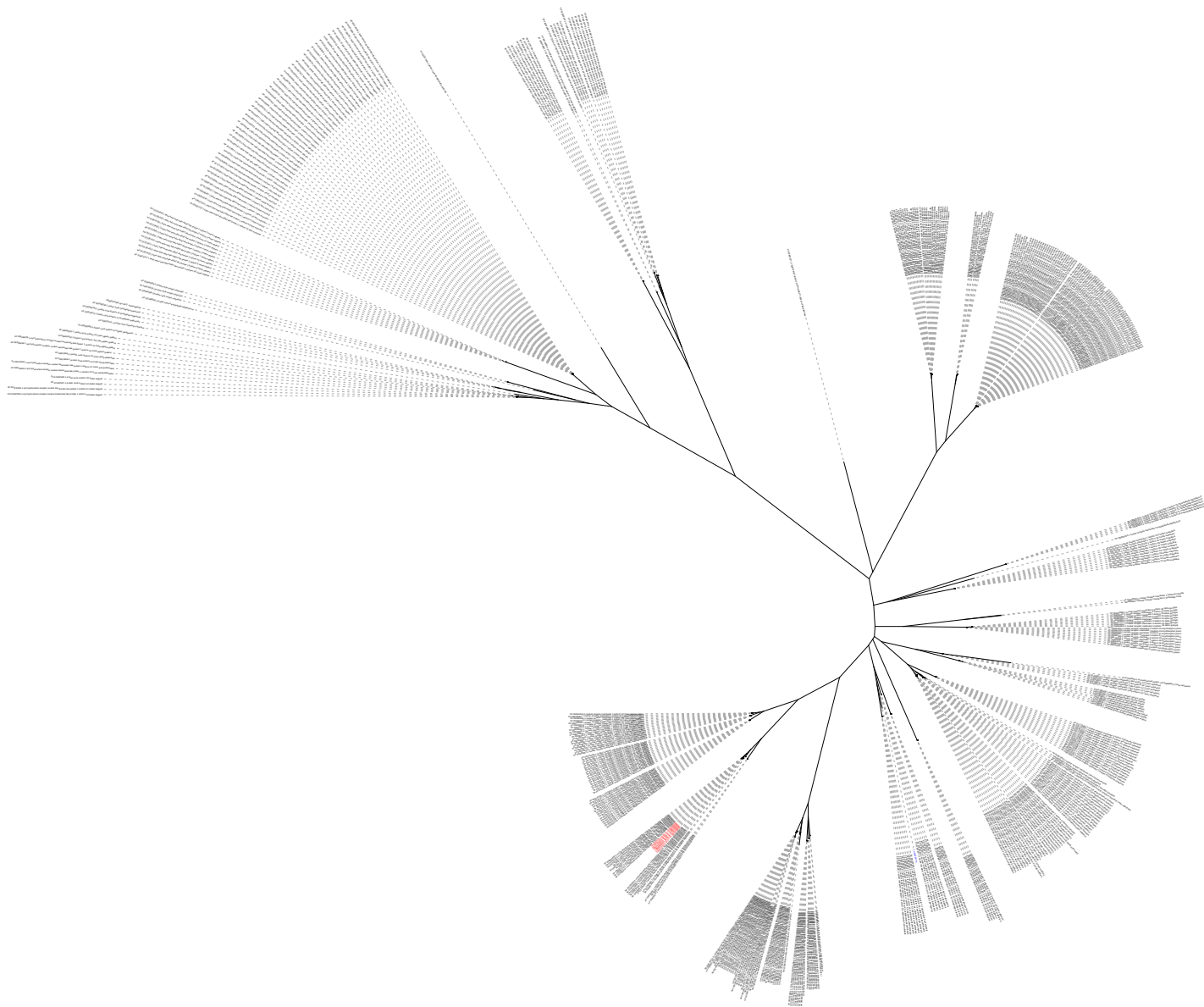

Supplement: Supplementary file 3 — Supplementary Material 3 [file 12863_2025_1397_MOESM3_ESM.zip › 3.Manually_checked_genes/4.Trees/tai.pdf]

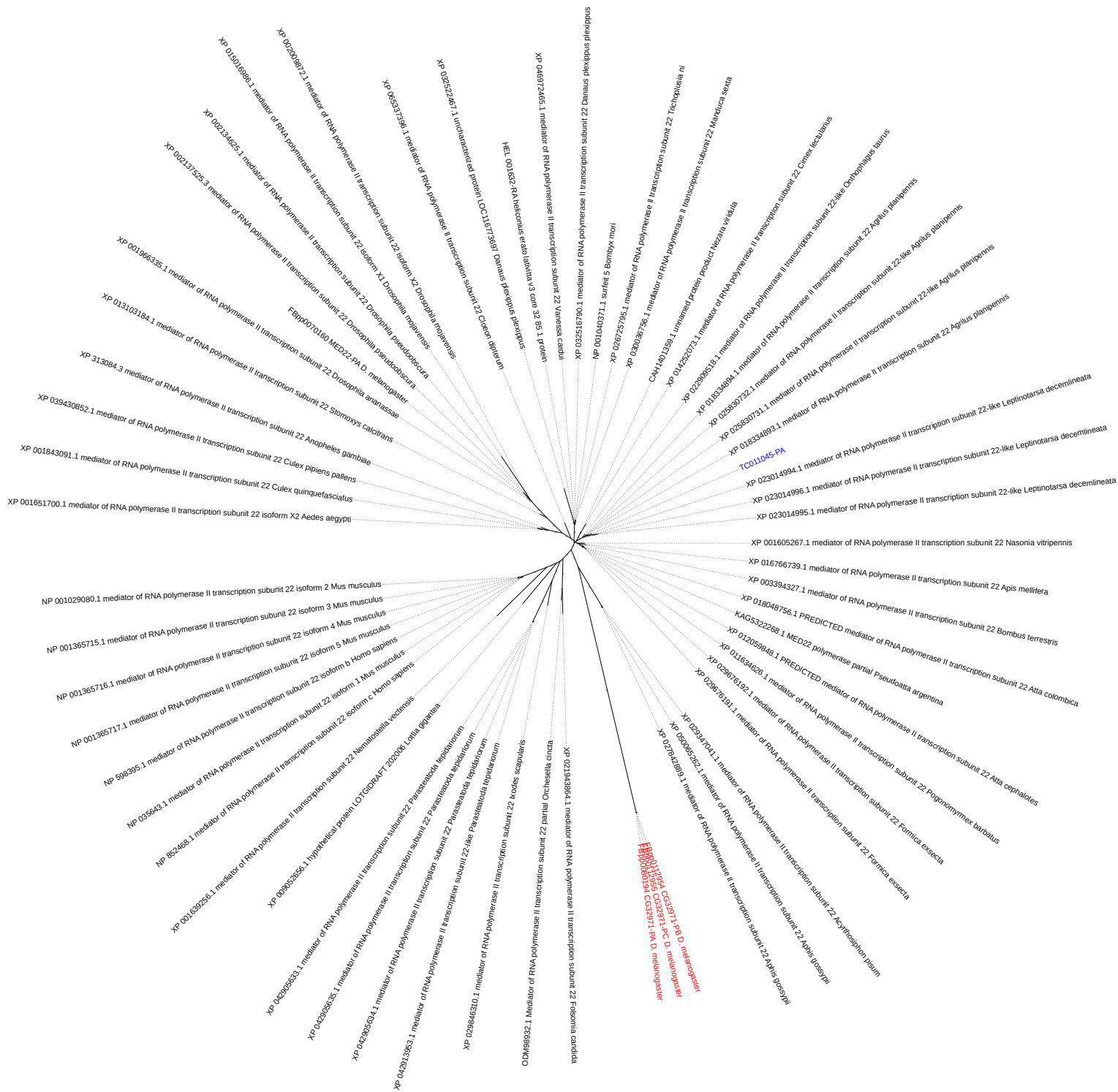

Supplement: Supplementary file 3 — Supplementary Material 3 [file 12863_2025_1397_MOESM3_ESM.zip › 3.Manually_checked_genes/4.Trees/CG32971.pdf]

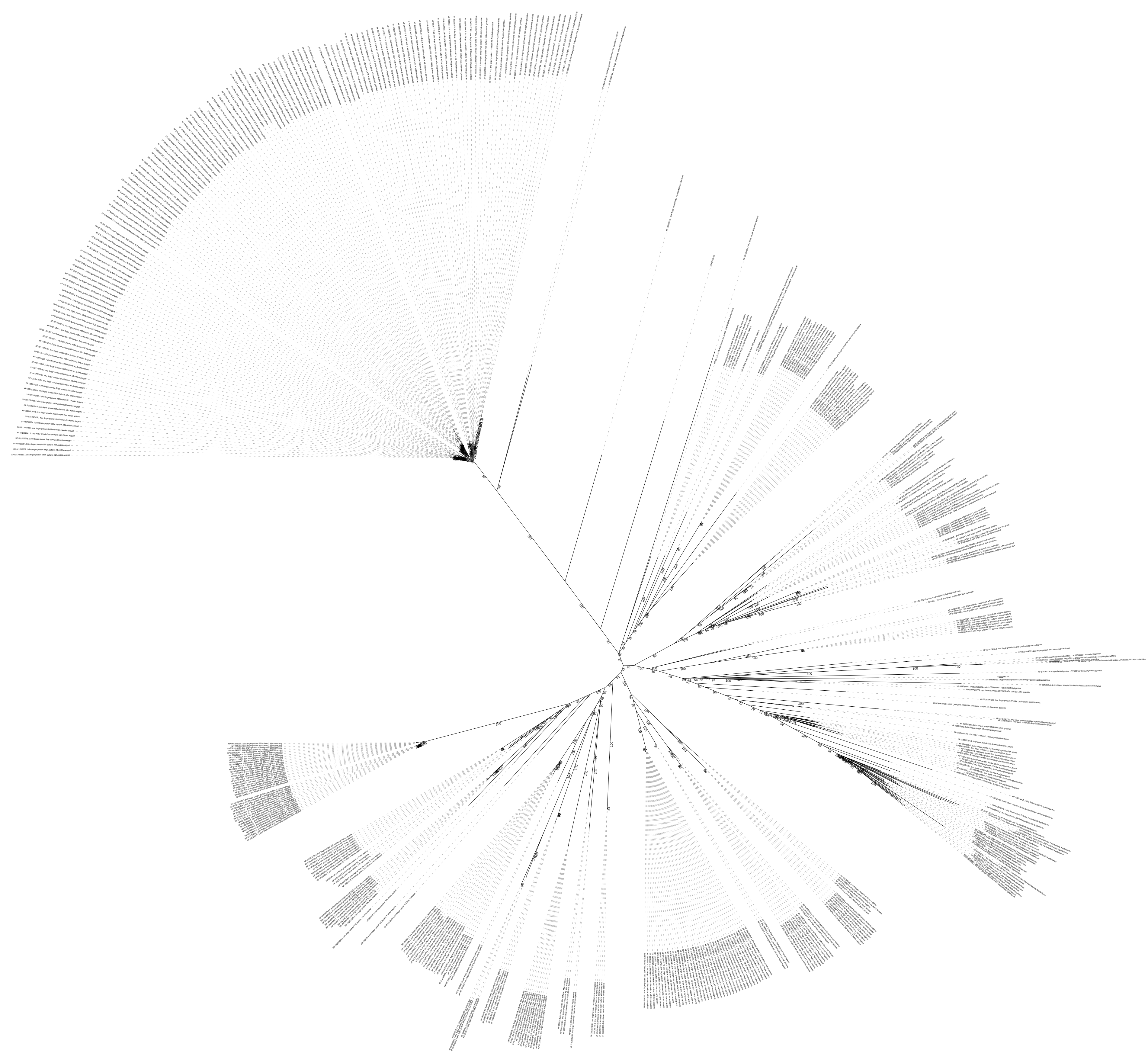

Supplement: Supplementary file 3 — Supplementary Material 3 [file 12863_2025_1397_MOESM3_ESM.zip › 3.Manually_checked_genes/4.Trees/CG30431.pdf]

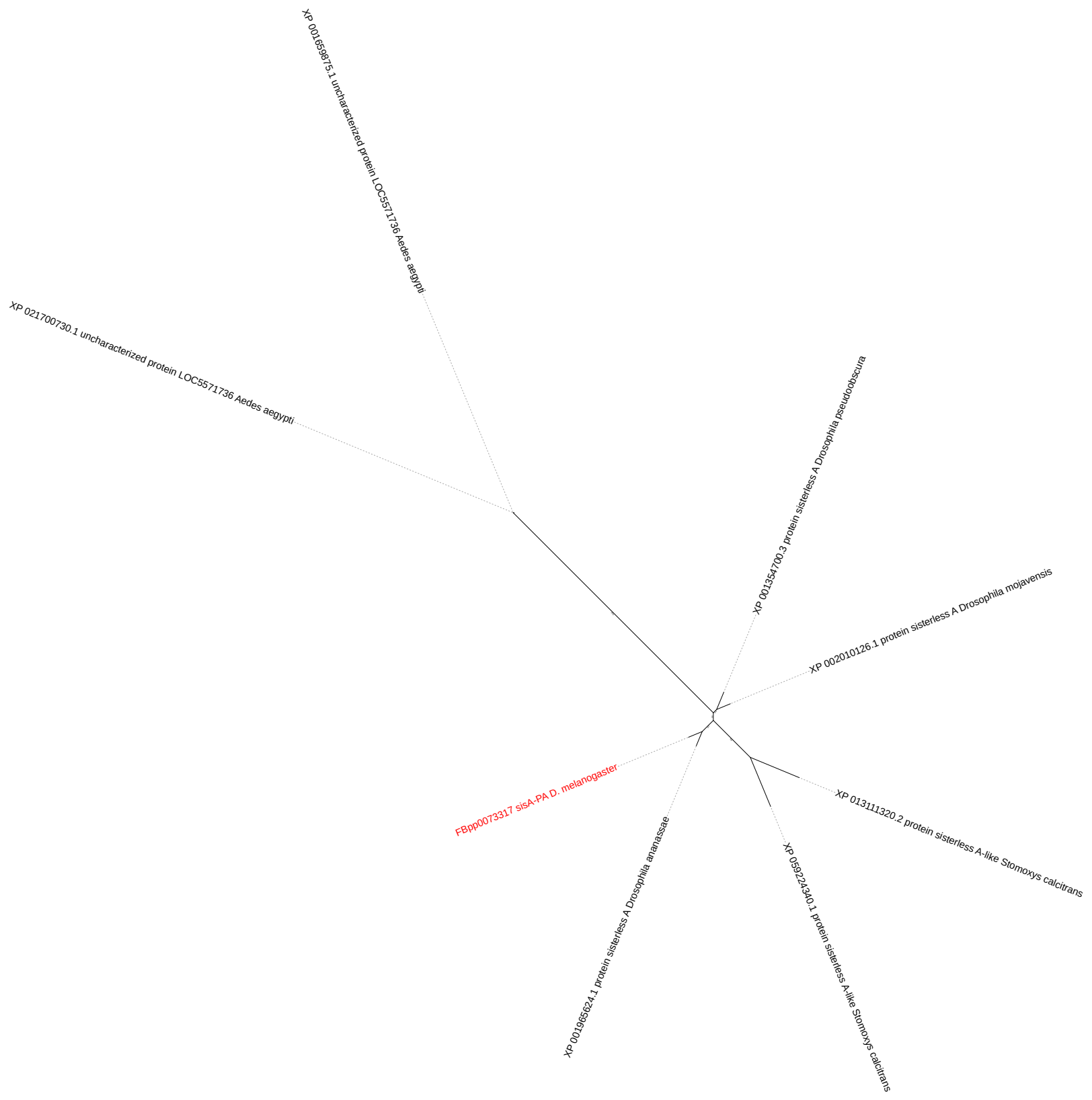

Supplement: Supplementary file 3 — Supplementary Material 3 [file 12863_2025_1397_MOESM3_ESM.zip › 3.Manually_checked_genes/4.Trees/sisA.pdf]

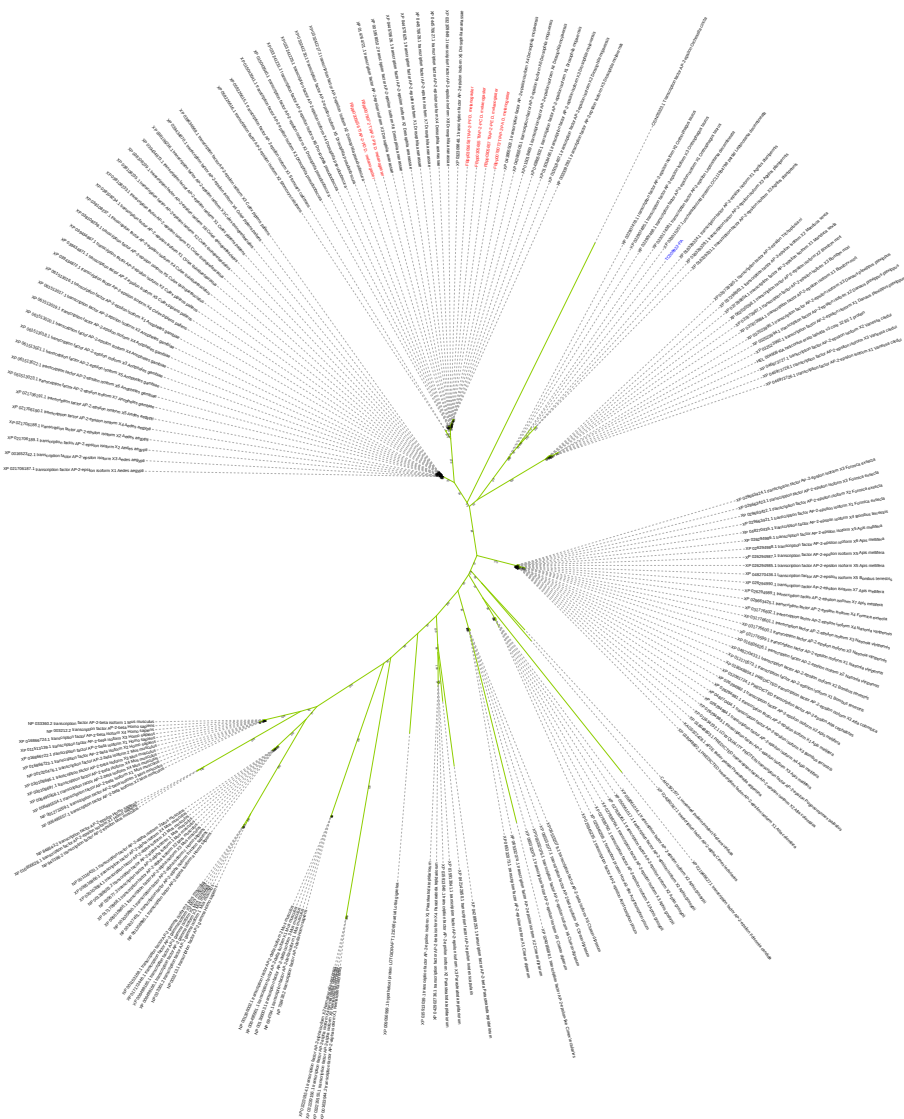

Supplement: Supplementary file 3 — Supplementary Material 3 [file 12863_2025_1397_MOESM3_ESM.zip › 3.Manually_checked_genes/4.Trees/TfAP-2.pdf]

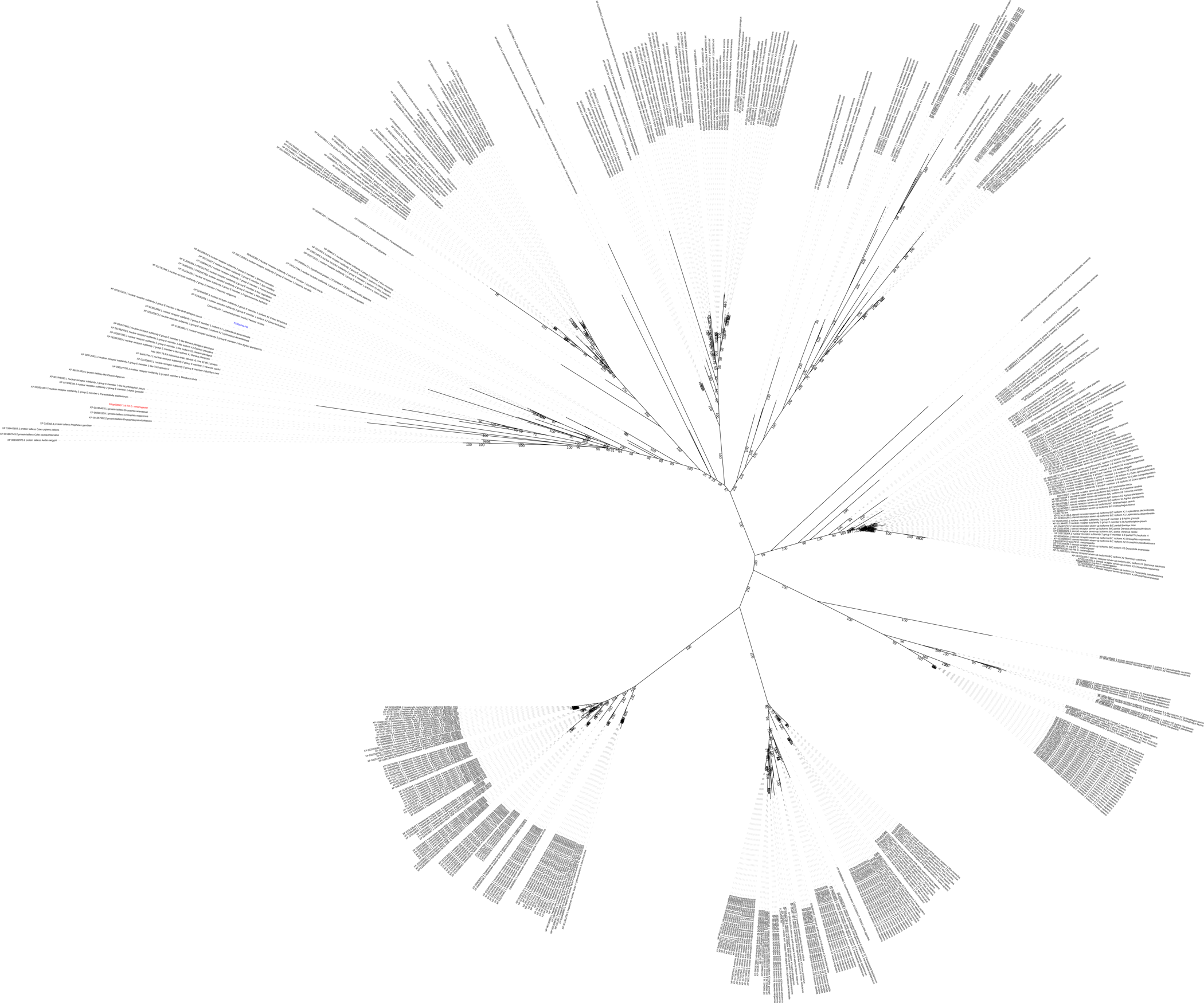

Supplement: Supplementary file 3 — Supplementary Material 3 [file 12863_2025_1397_MOESM3_ESM.zip › 3.Manually_checked_genes/4.Trees/tll.pdf]

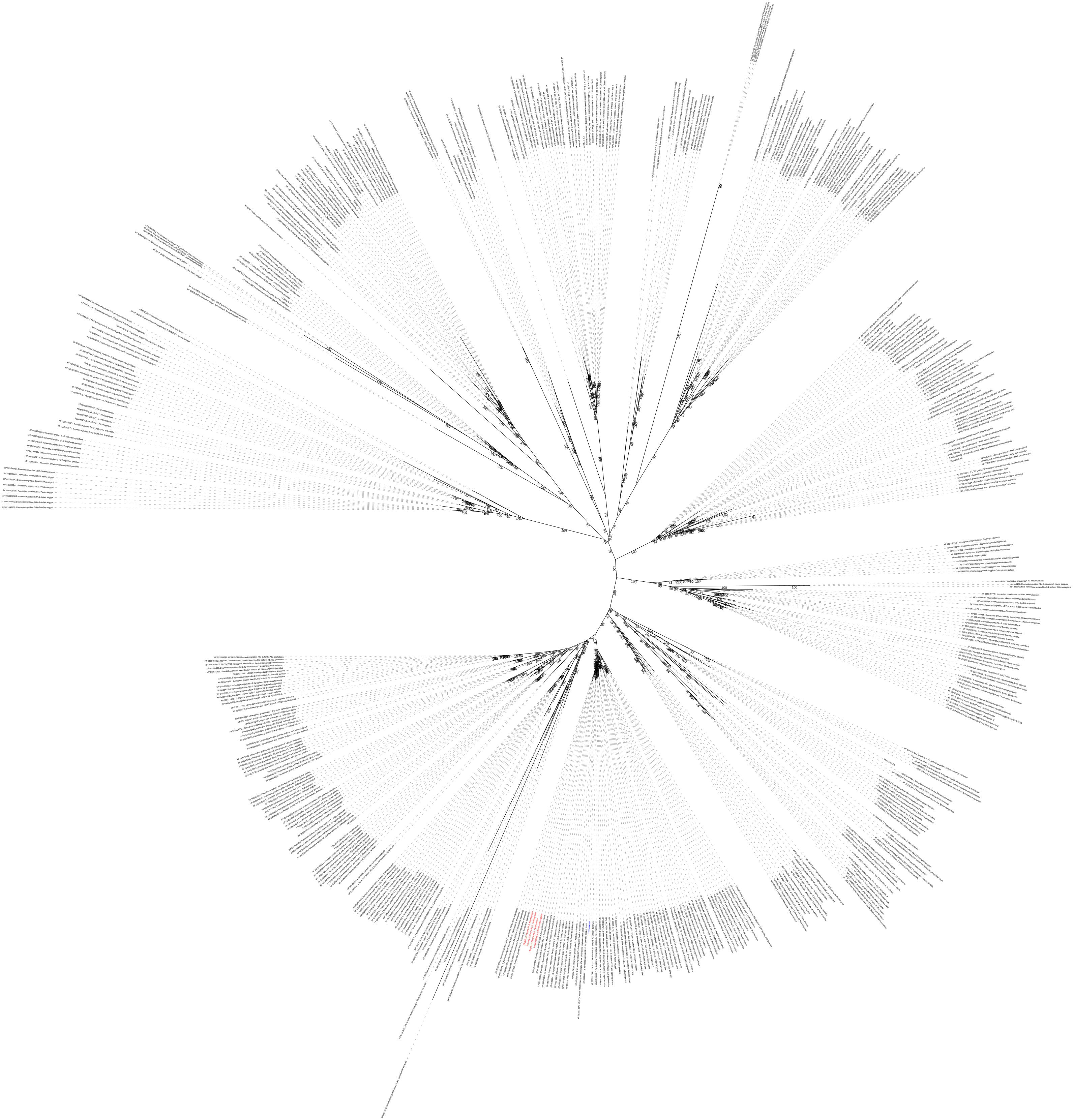

Supplement: Supplementary file 3 — Supplementary Material 3 [file 12863_2025_1397_MOESM3_ESM.zip › 3.Manually_checked_genes/4.Trees/scro.pdf]

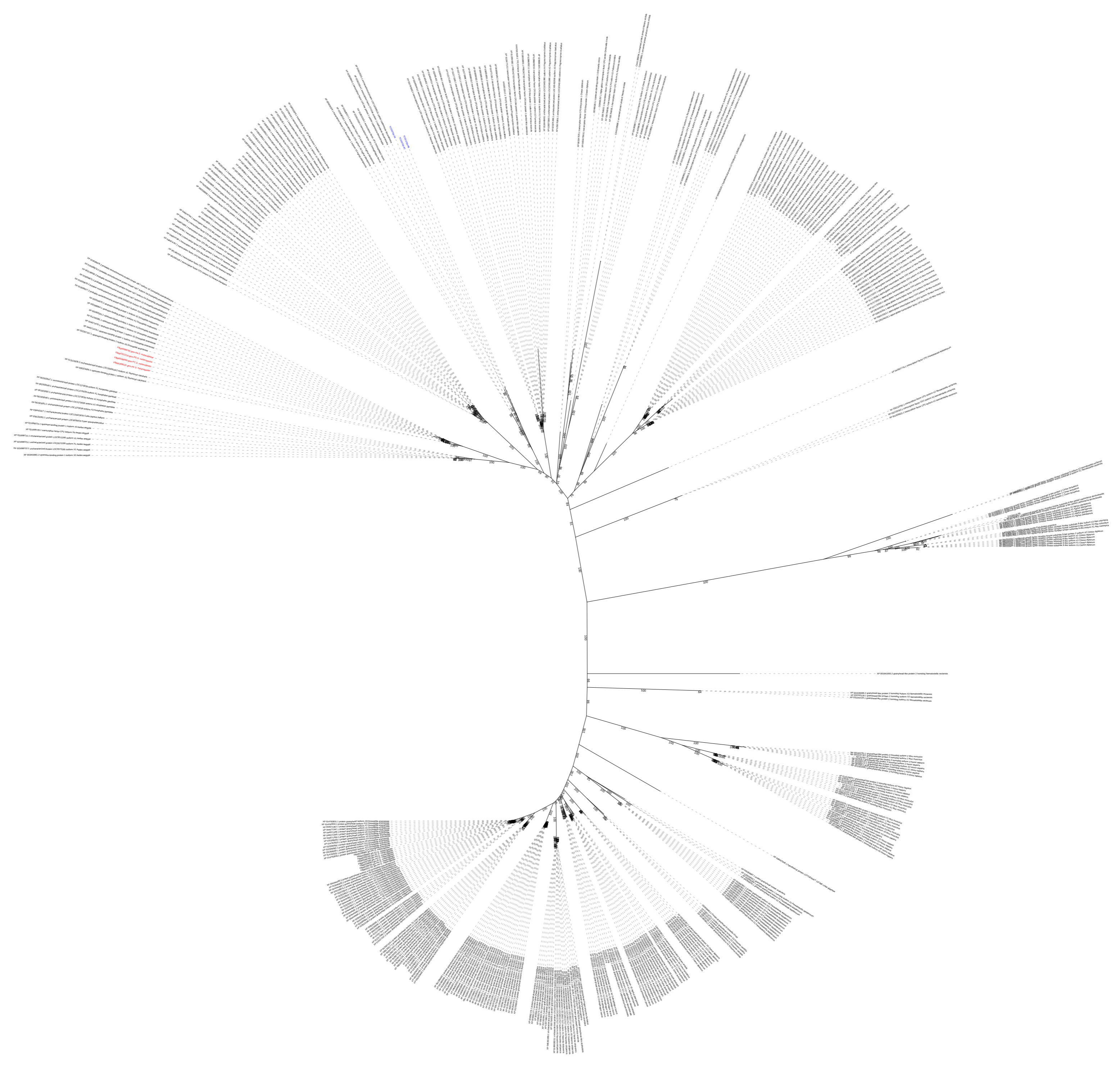

Supplement: Supplementary file 3 — Supplementary Material 3 [file 12863_2025_1397_MOESM3_ESM.zip › 3.Manually_checked_genes/4.Trees/gem.pdf]

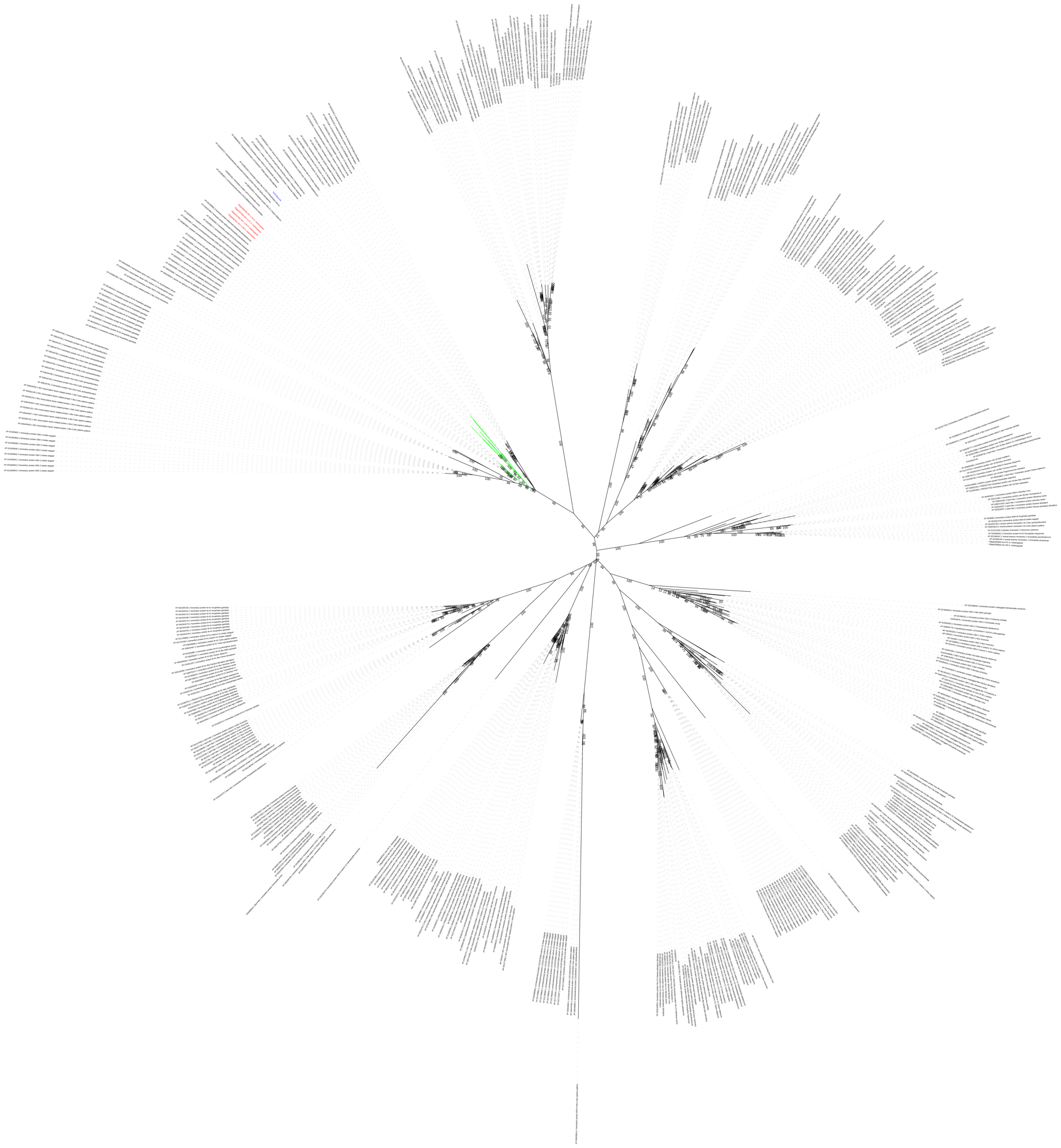

Supplement: Supplementary file 3 — Supplementary Material 3 [file 12863_2025_1397_MOESM3_ESM.zip › 3.Manually_checked_genes/4.Trees/NK7.1.pdf]

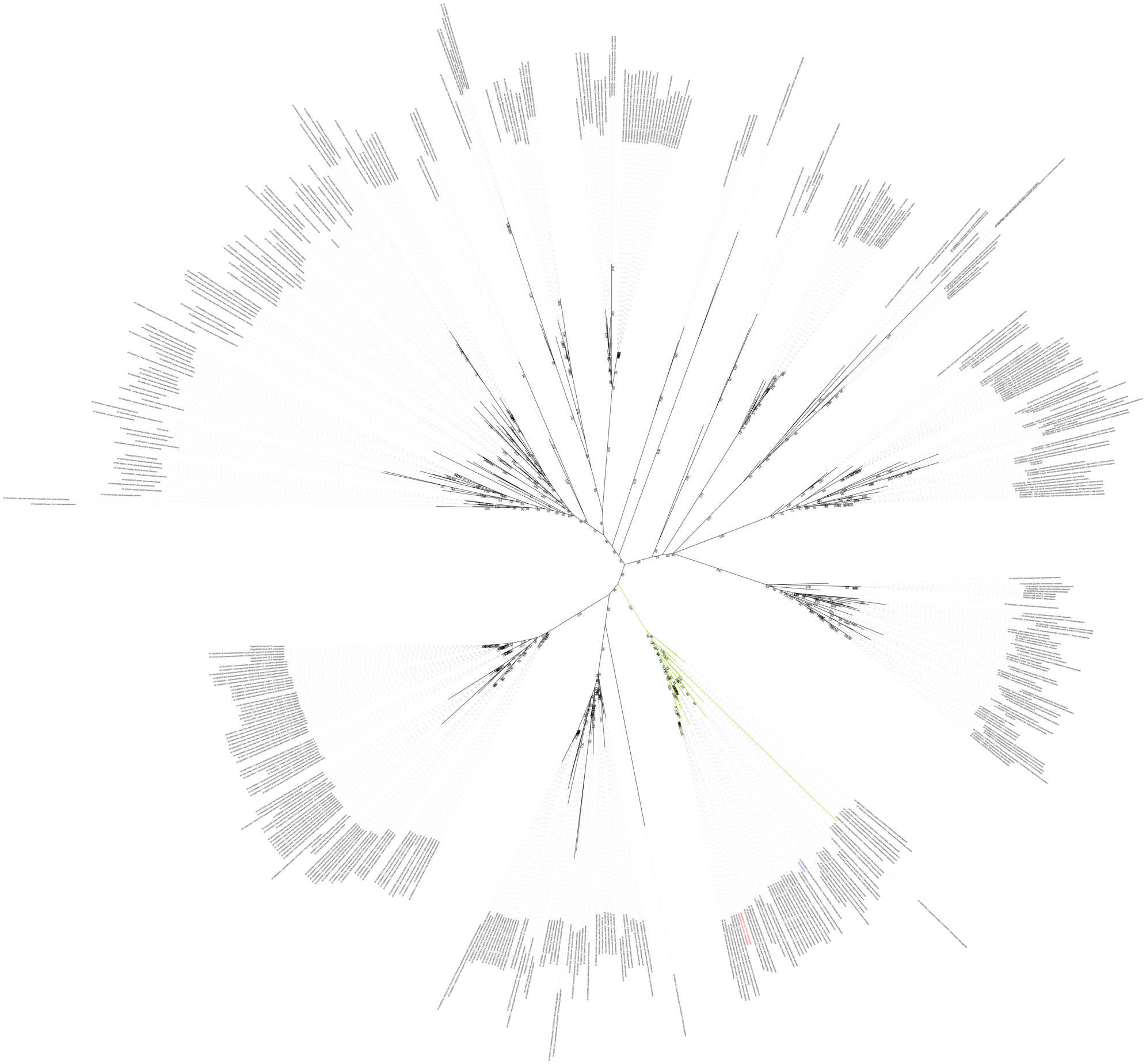

Supplement: Supplementary file 3 — Supplementary Material 3 [file 12863_2025_1397_MOESM3_ESM.zip › 3.Manually_checked_genes/4.Trees/Fer1.pdf]

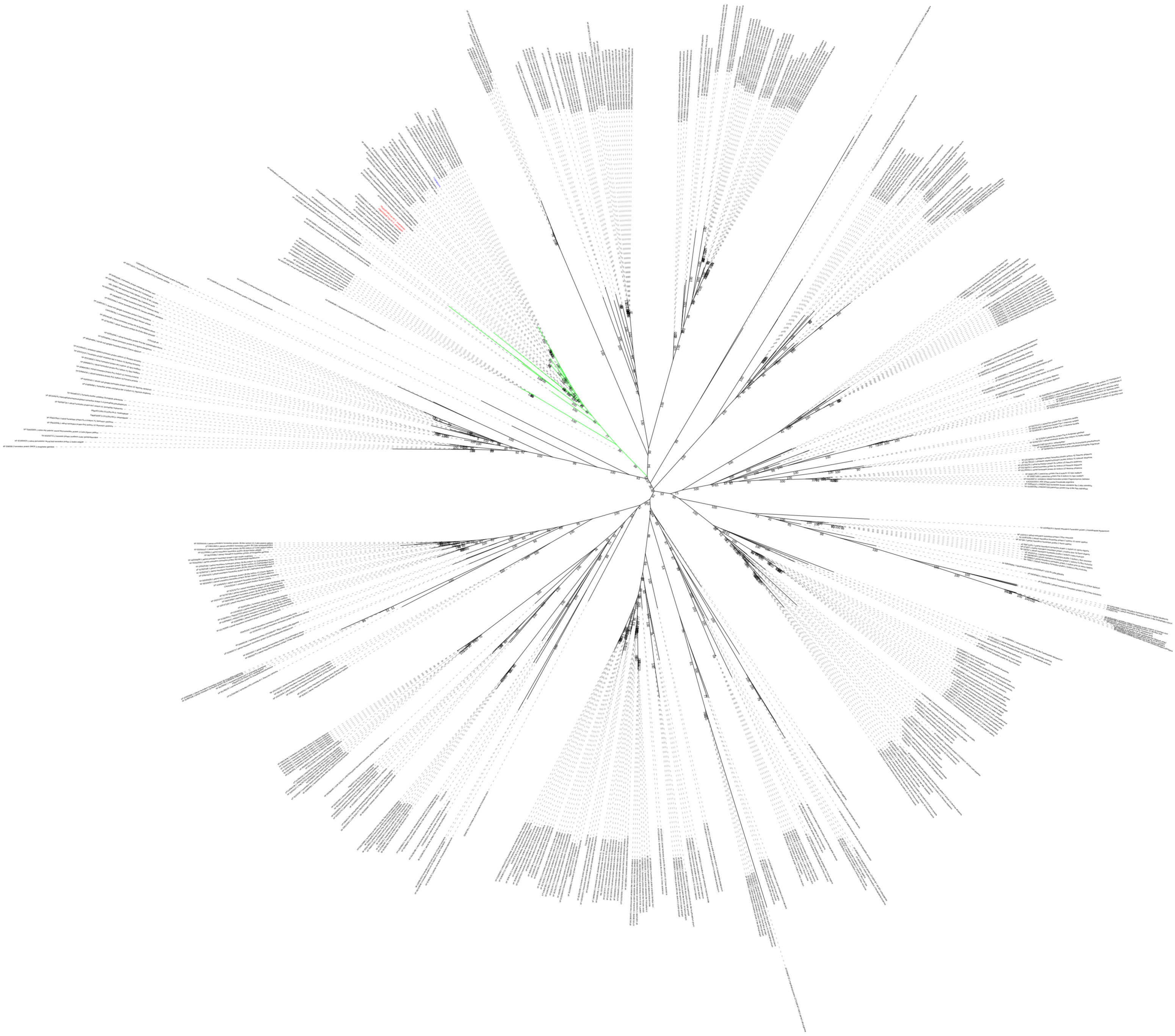

Supplement: Supplementary file 3 — Supplementary Material 3 [file 12863_2025_1397_MOESM3_ESM.zip › 3.Manually_checked_genes/4.Trees/Drgx.pdf]

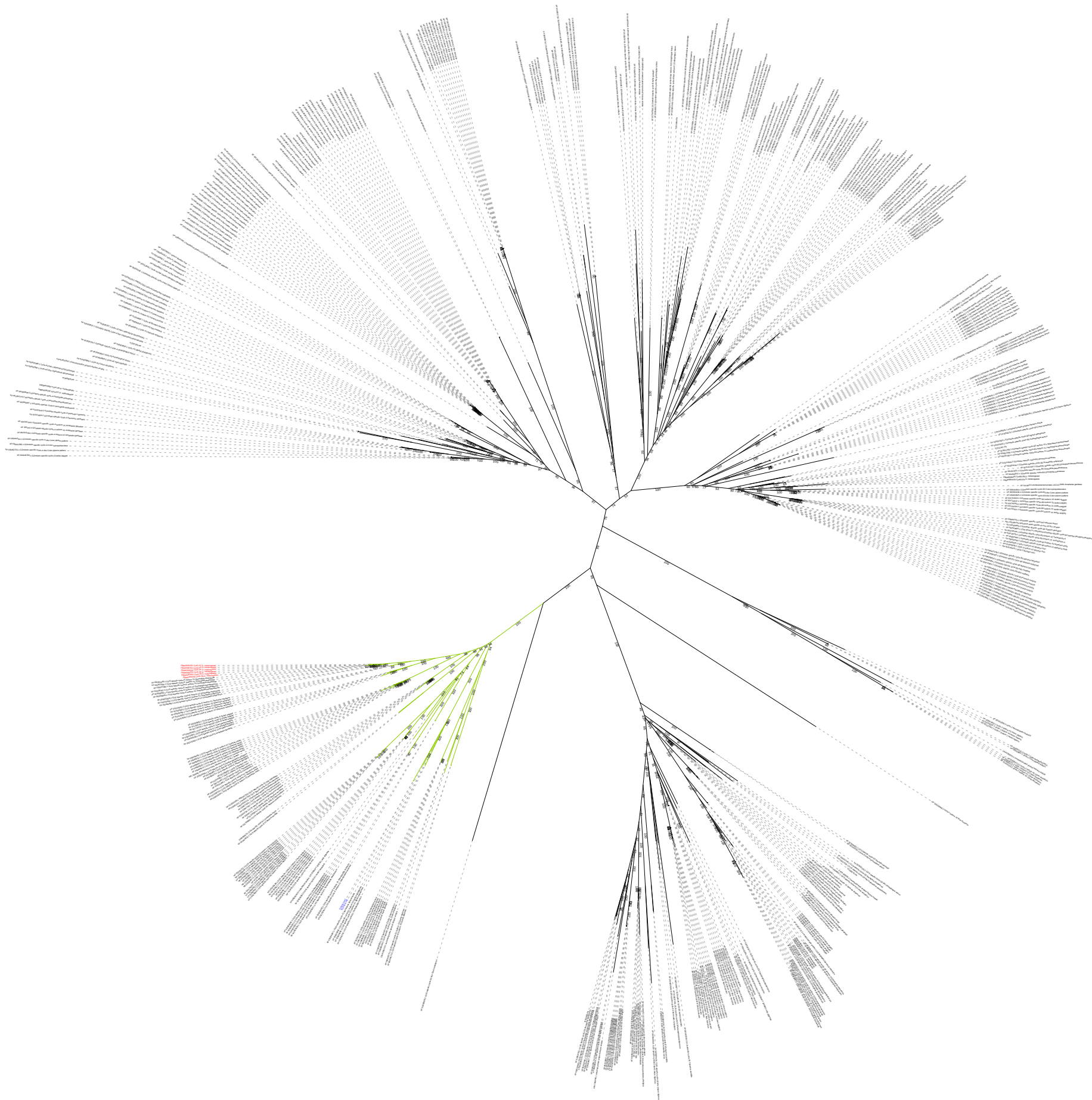

Supplement: Supplementary file 3 — Supplementary Material 3 [file 12863_2025_1397_MOESM3_ESM.zip › 3.Manually_checked_genes/4.Trees/CycE.pdf]

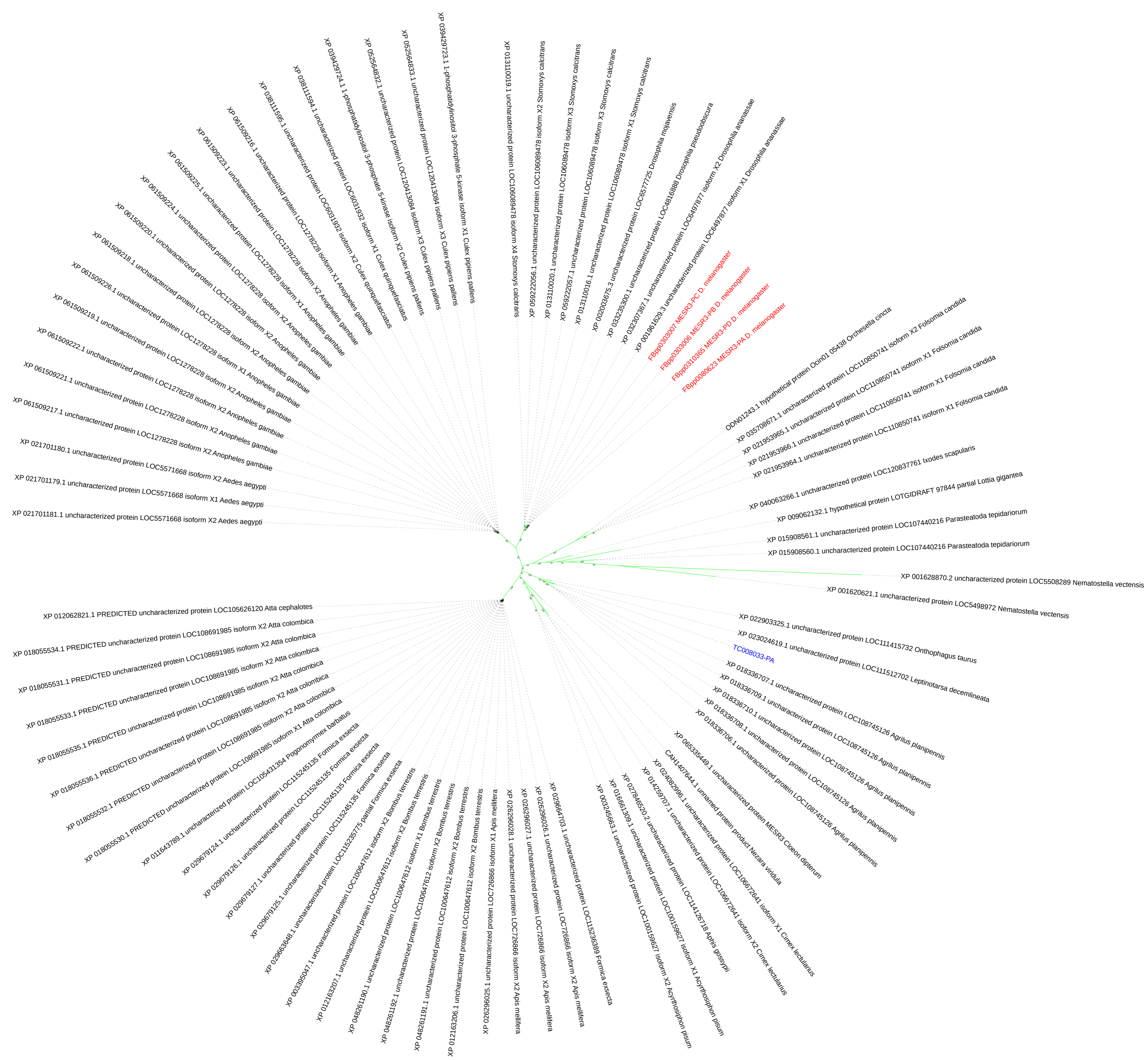

Supplement: Supplementary file 3 — Supplementary Material 3 [file 12863_2025_1397_MOESM3_ESM.zip › 3.Manually_checked_genes/4.Trees/MESR3.pdf]

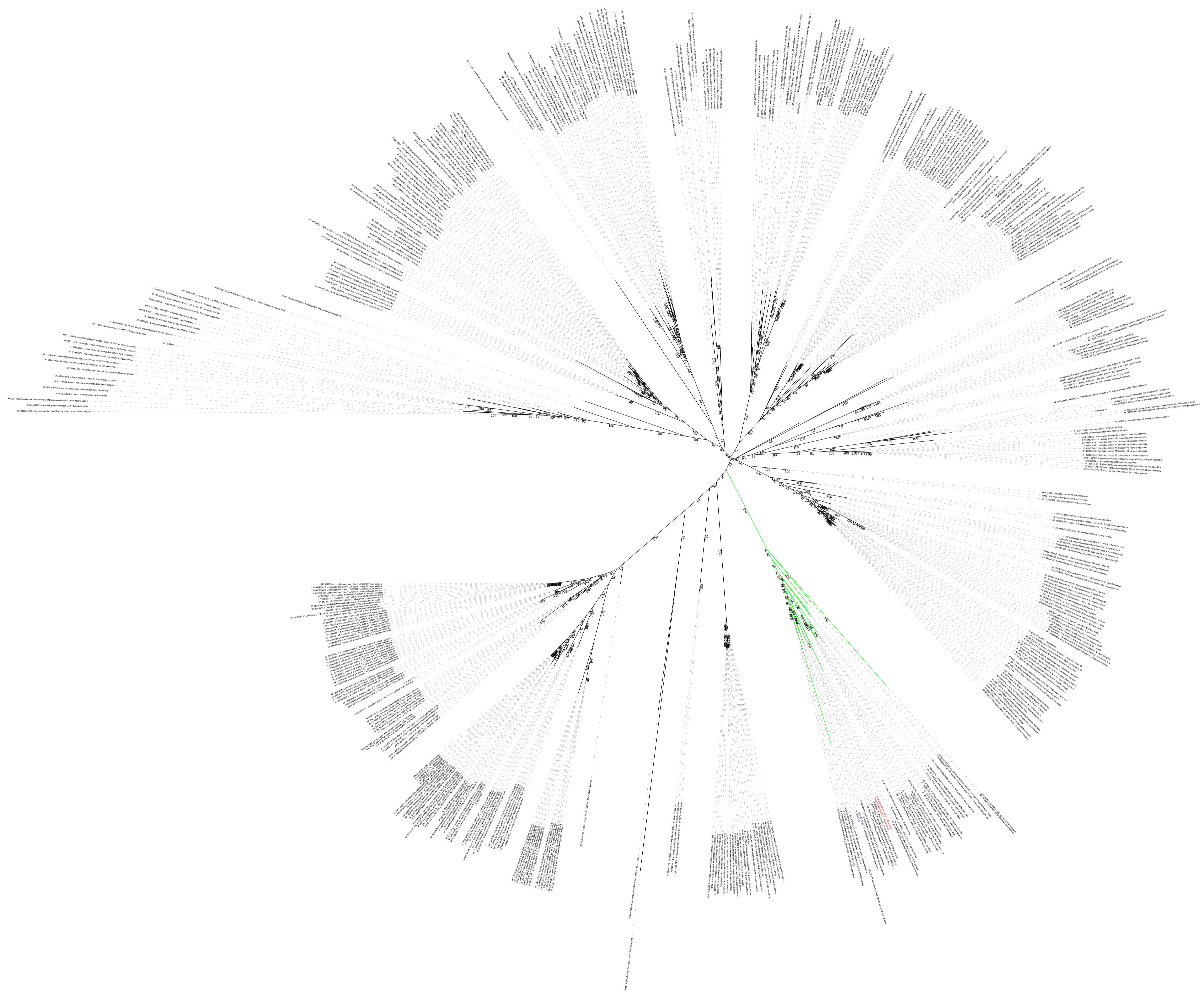

Supplement: Supplementary file 3 — Supplementary Material 3 [file 12863_2025_1397_MOESM3_ESM.zip › 3.Manually_checked_genes/4.Trees/Gsc.pdf]

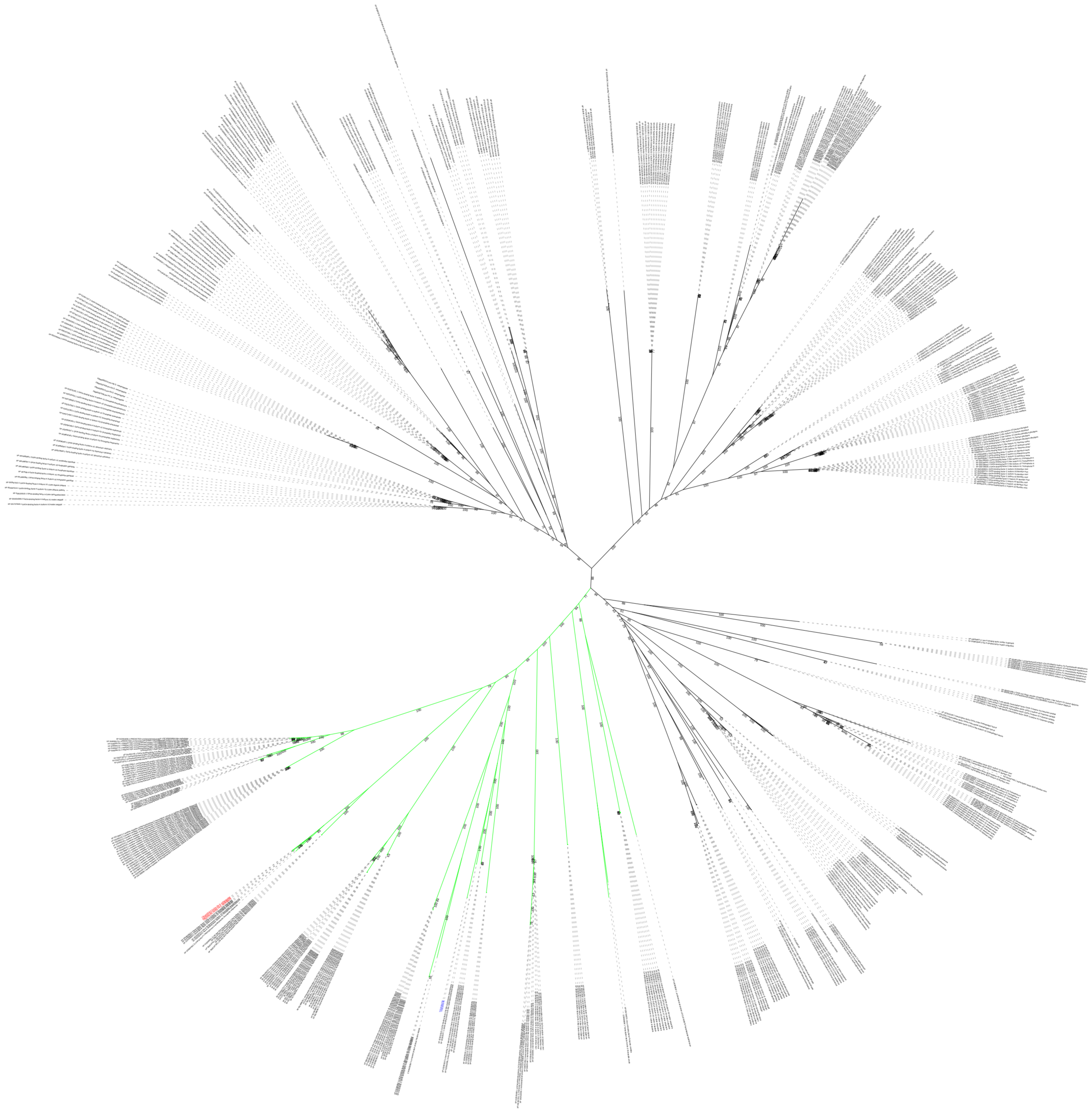

Supplement: Supplementary file 3 — Supplementary Material 3 [file 12863_2025_1397_MOESM3_ESM.zip › 3.Manually_checked_genes/4.Trees/GATAe.pdf]

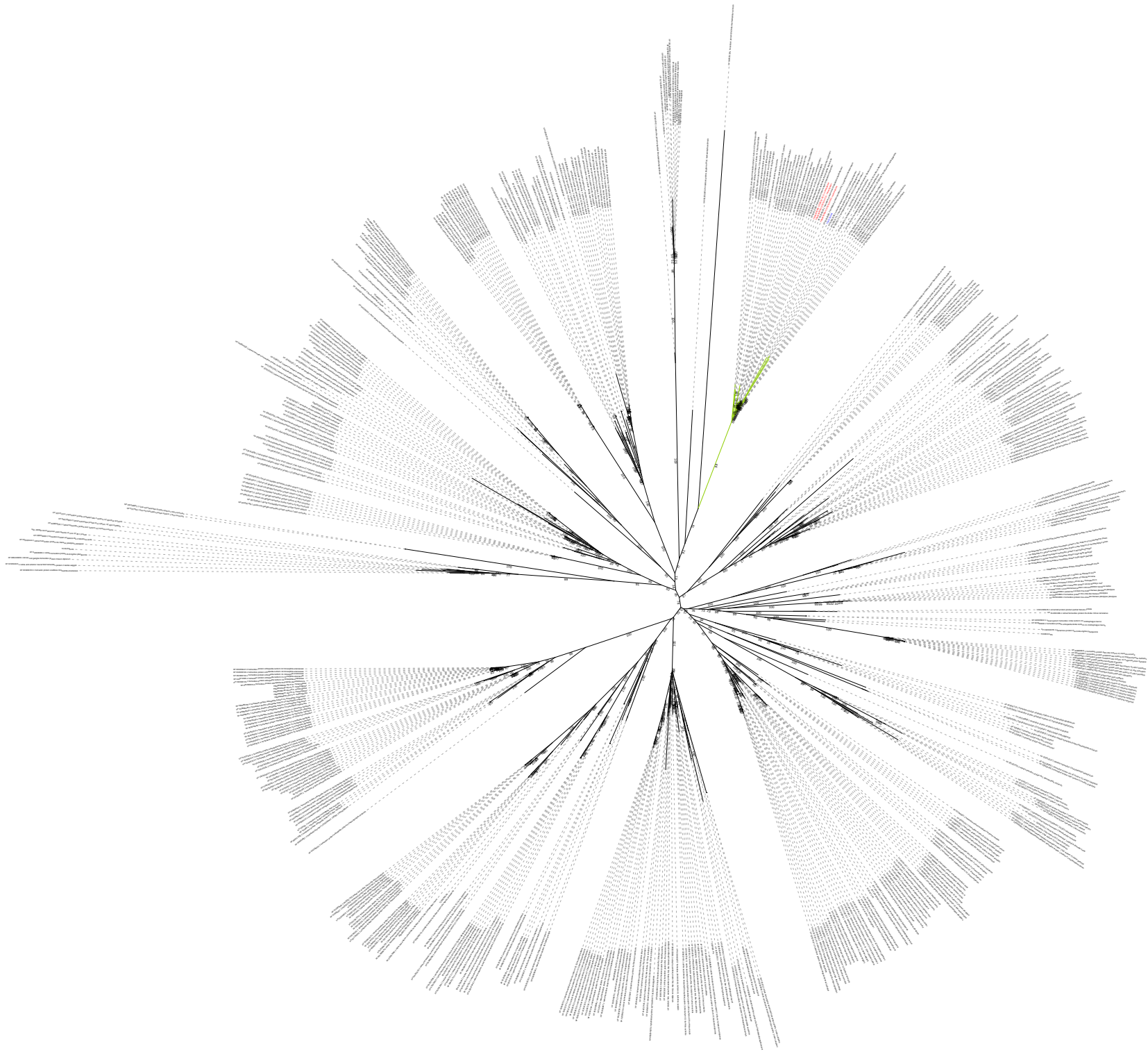

Supplement: Supplementary file 3 — Supplementary Material 3 [file 12863_2025_1397_MOESM3_ESM.zip › 3.Manually_checked_genes/4.Trees/CG32532.pdf]

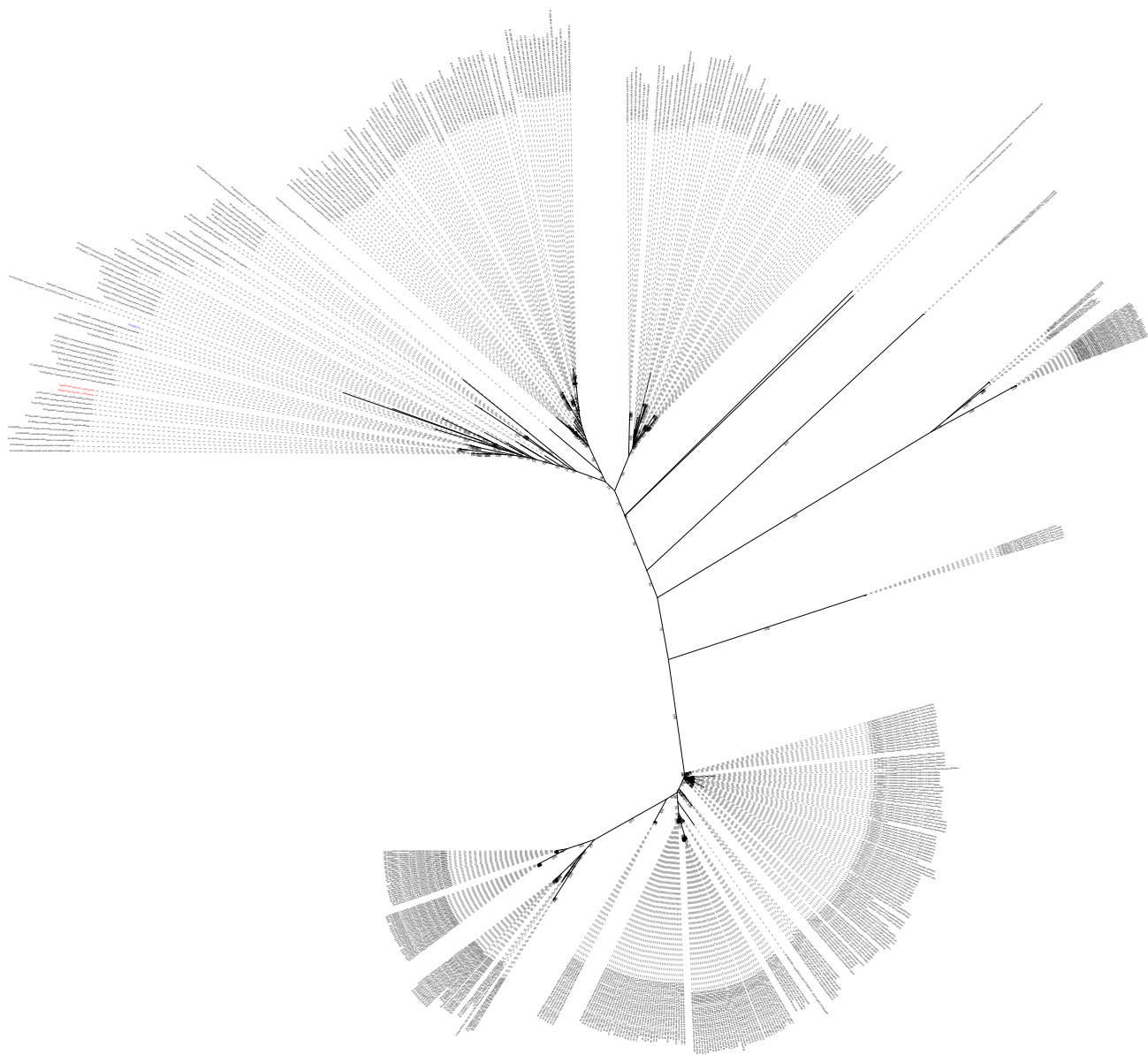

Supplement: Supplementary file 3 — Supplementary Material 3 [file 12863_2025_1397_MOESM3_ESM.zip › 3.Manually_checked_genes/4.Trees/Six4.pdf]

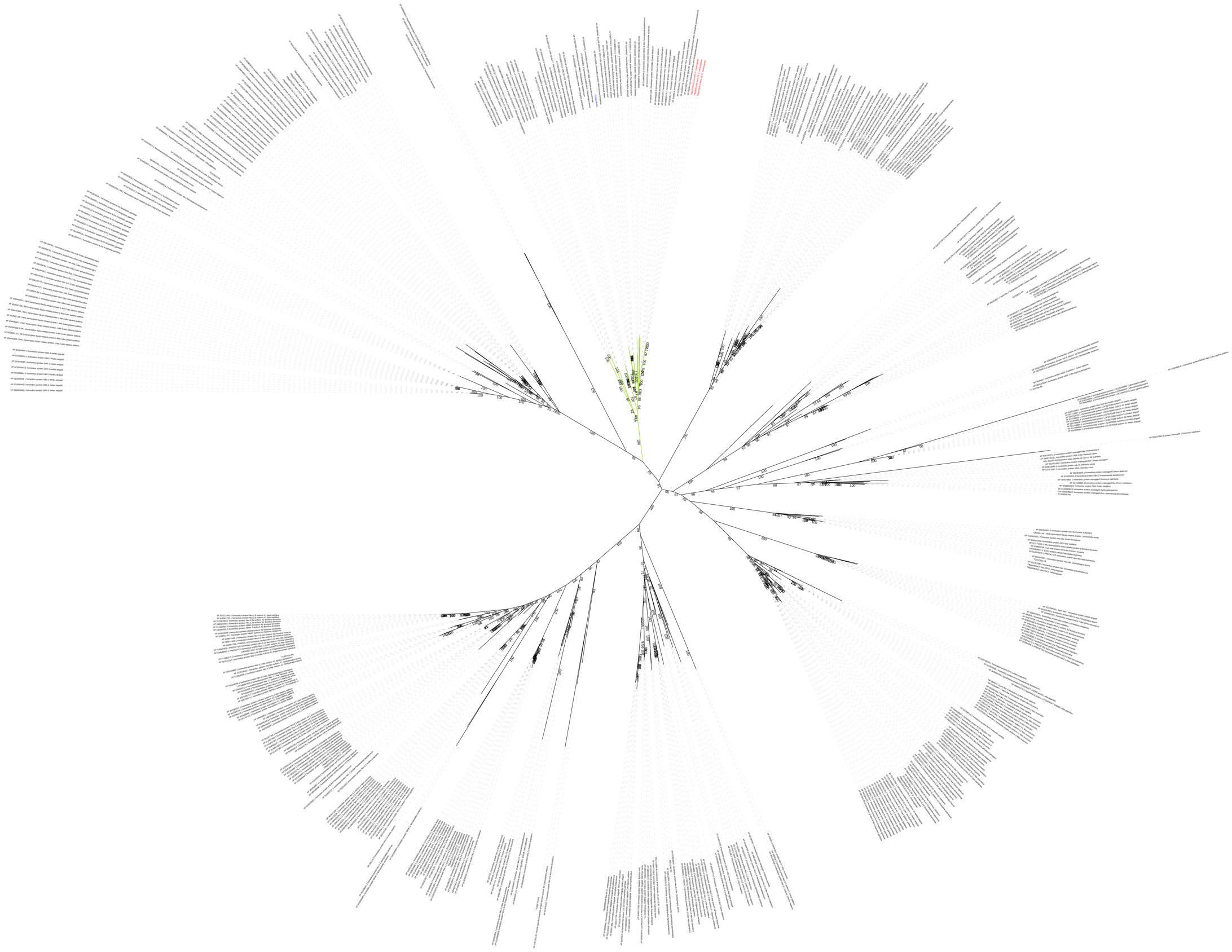

Supplement: Supplementary file 3 — Supplementary Material 3 [file 12863_2025_1397_MOESM3_ESM.zip › 3.Manually_checked_genes/4.Trees/Hmx.pdf]

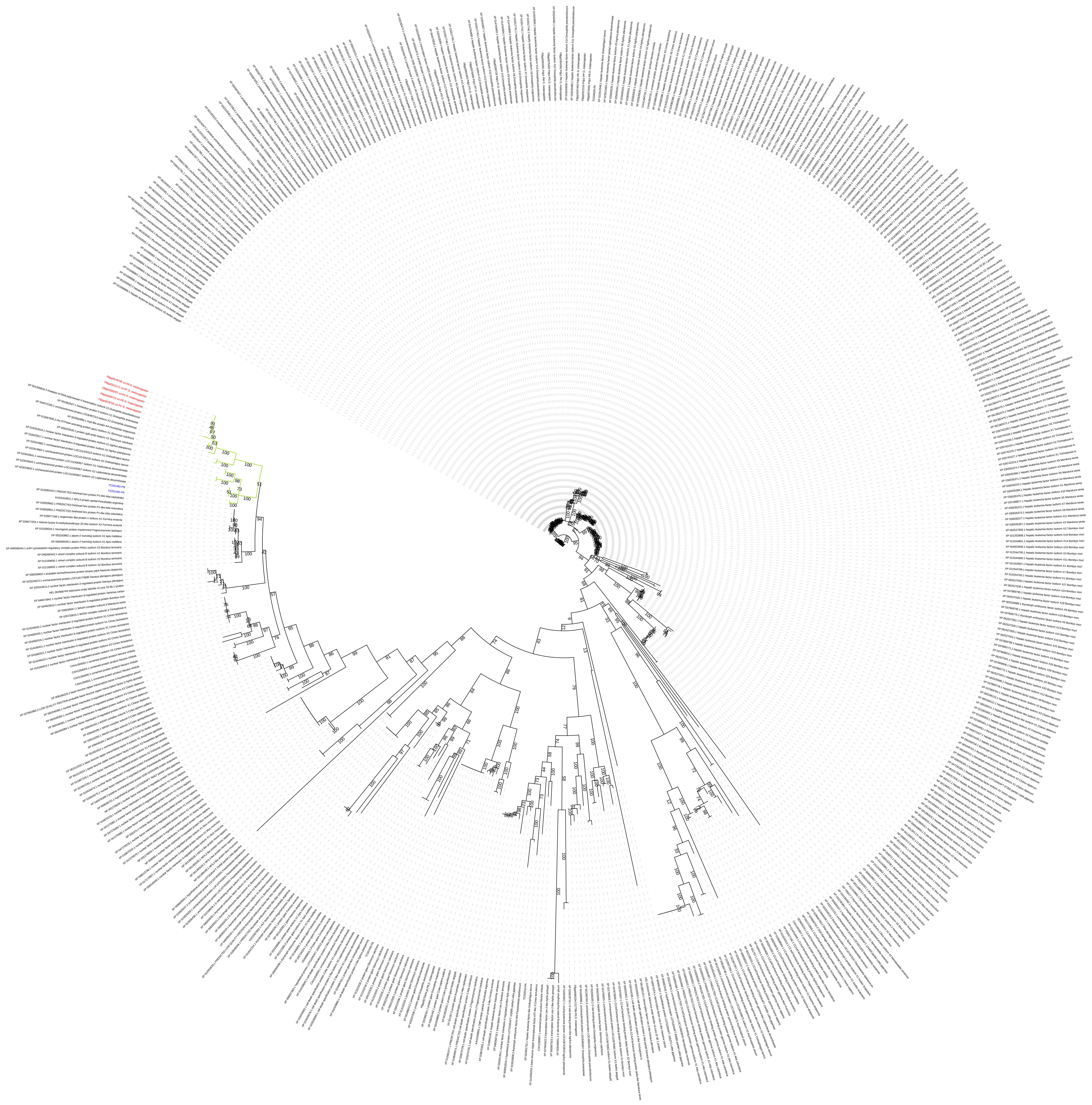

Supplement: Supplementary file 3 — Supplementary Material 3 [file 12863_2025_1397_MOESM3_ESM.zip › 3.Manually_checked_genes/4.Trees/vri.pdf]

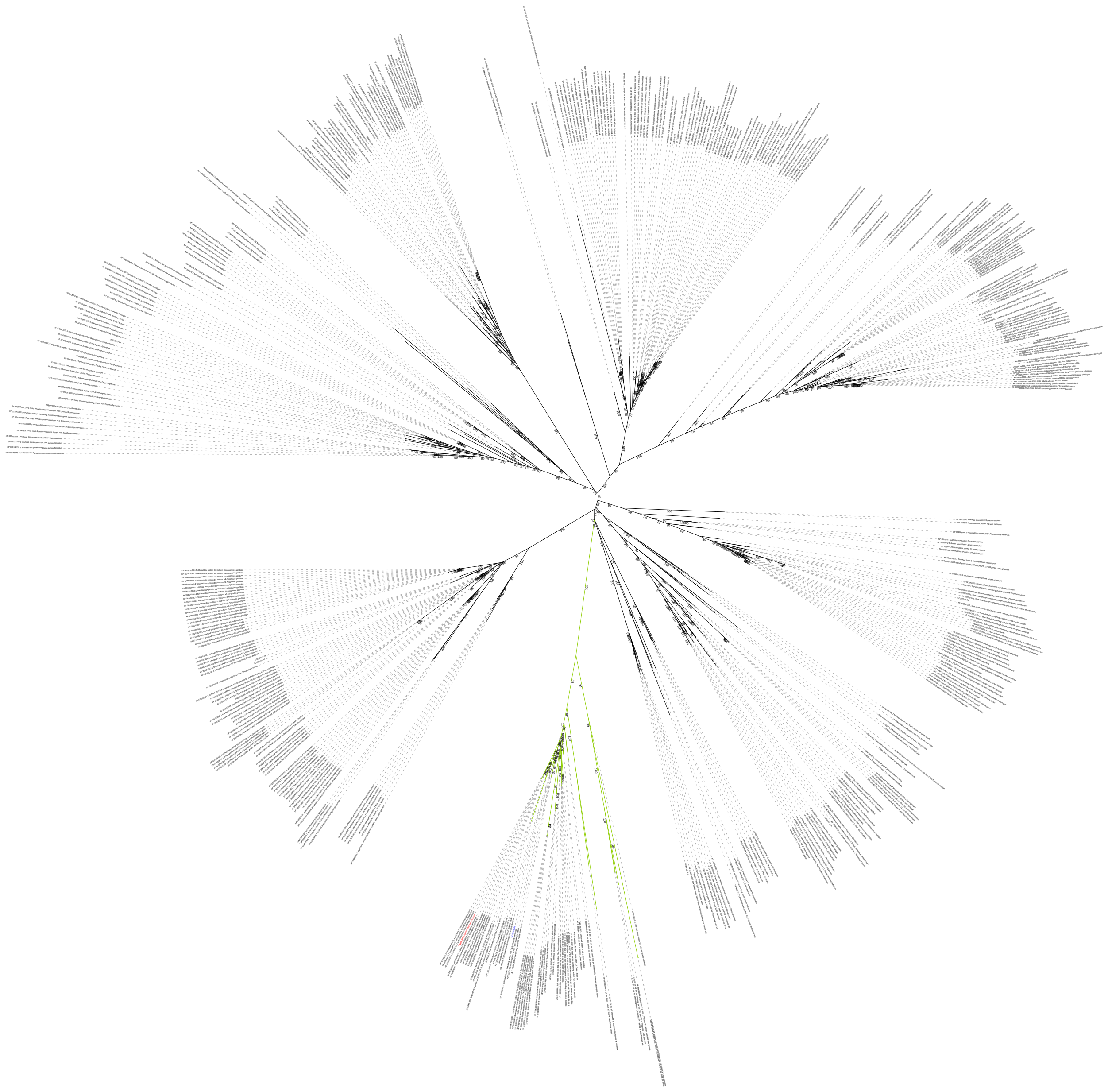

Supplement: Supplementary file 3 — Supplementary Material 3 [file 12863_2025_1397_MOESM3_ESM.zip › 3.Manually_checked_genes/4.Trees/fd102C.pdf]

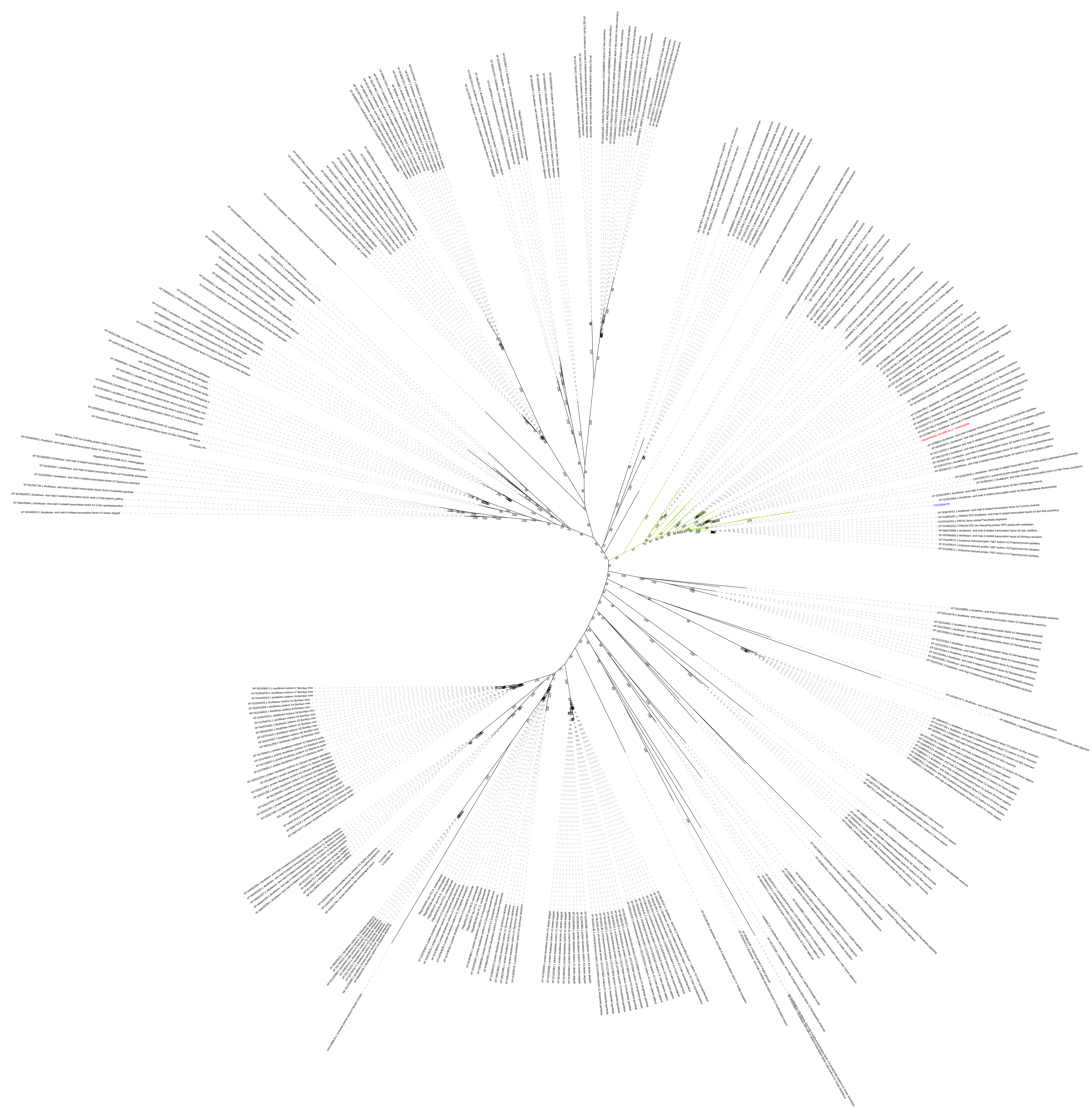

Supplement: Supplementary file 3 — Supplementary Material 3 [file 12863_2025_1397_MOESM3_ESM.zip › 3.Manually_checked_genes/4.Trees/dmrt99B.pdf]

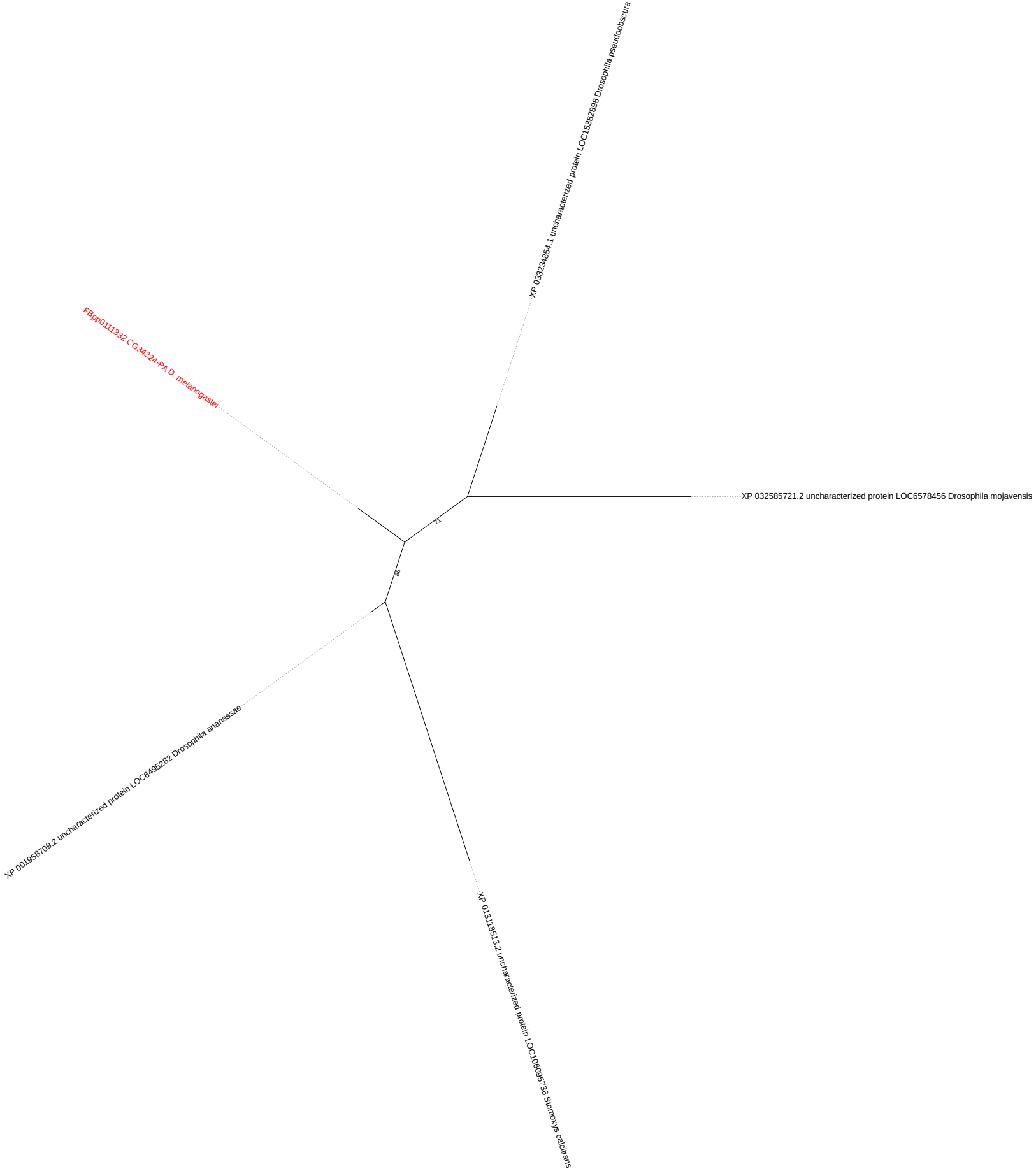

Supplement: Supplementary file 3 — Supplementary Material 3 [file 12863_2025_1397_MOESM3_ESM.zip › 3.Manually_checked_genes/4.Trees/CG34224.pdf]

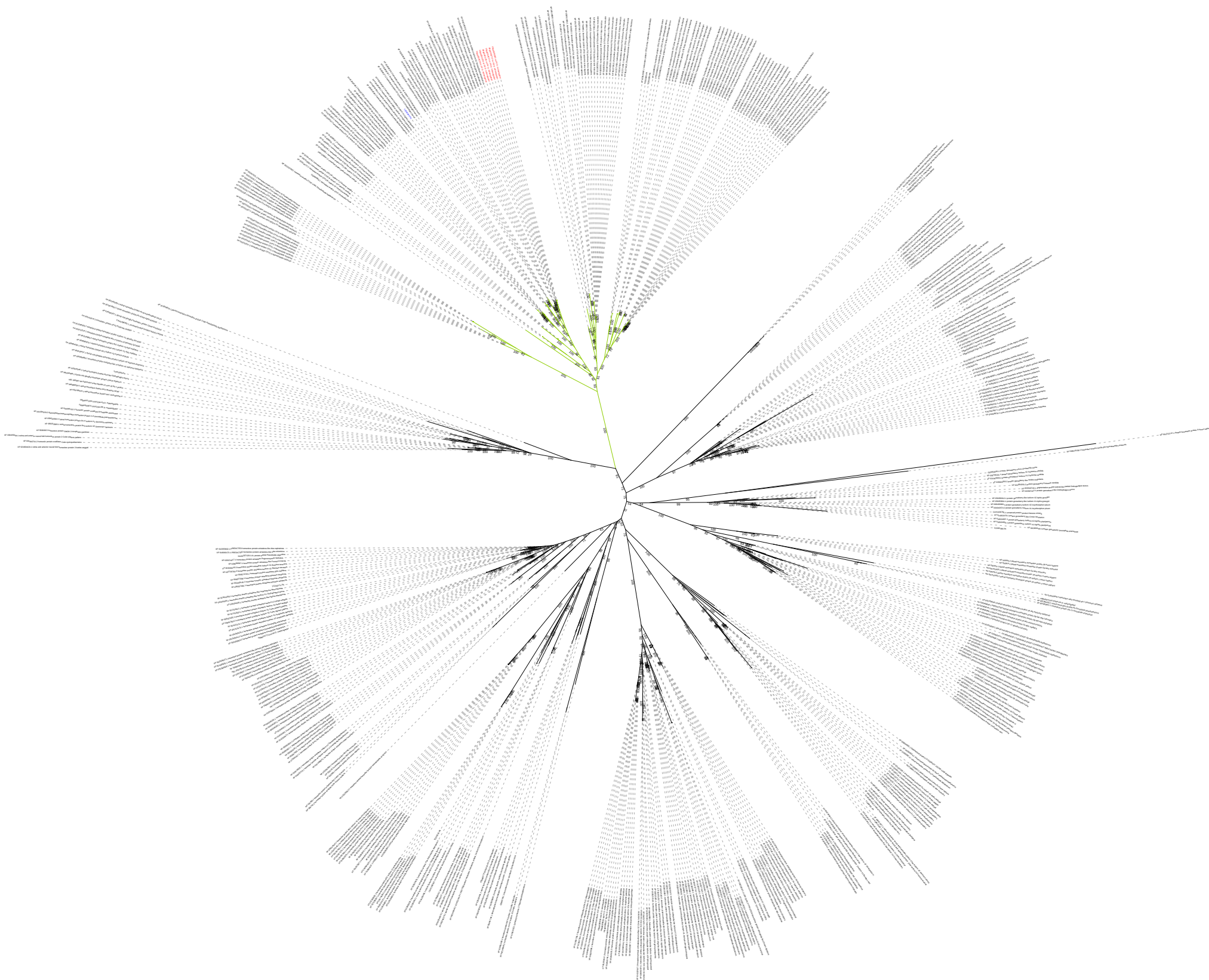

Supplement: Supplementary file 3 — Supplementary Material 3 [file 12863_2025_1397_MOESM3_ESM.zip › 3.Manually_checked_genes/4.Trees/oc.pdf]
