# Supplementary material for: Orthologous genes of the red flour beetle Tribolium castaneum and the vinegar fly Drosophila melanogaster
Source: BMC Genom Data. 2025 Dec 12;27:17. doi: 10.1186/s12863-025-01397-0 (PMC12903232; doi:10.1186/s12863-025-01397-0)
Supplement: Supplementary file 3 — Supplementary Material 3 [file disco-r.pdf › 3.Manually_checked_genes/4.Trees/disco-r.pdf]

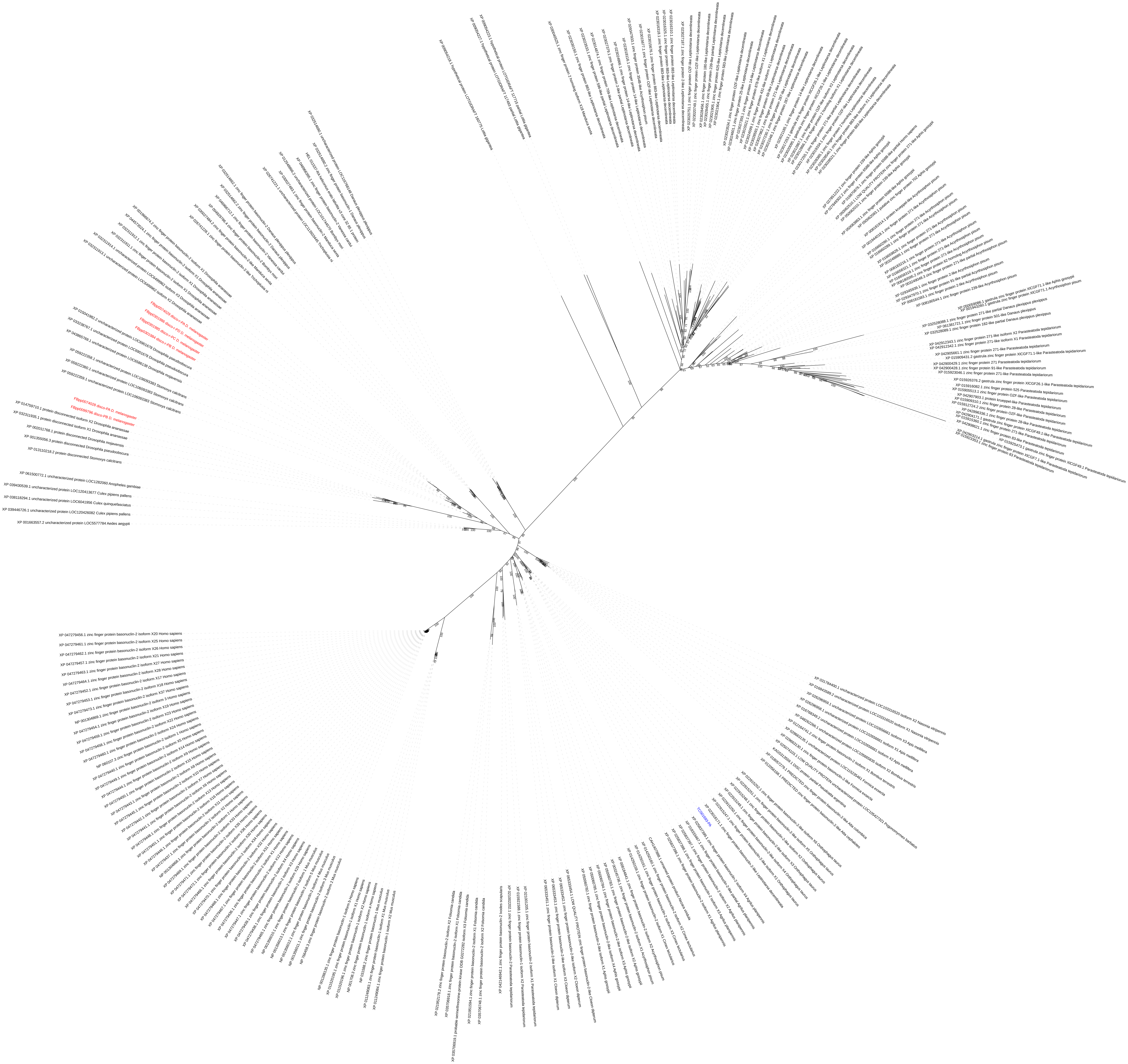
XP 032311935.1 protein disconnected isoform X1 Drosophila ananassae
XP 002011768.1 protein disconnected Drosophila mojavensis
XP 001355056.3 protein disconnected Drosophila pseudoobscura
XP 013110218.2 protein disconnected Stomoxys calcitrans
XP 061500772.1 uncharacterized protein LOC1282060 Anopheles gambiae
XP 039430539.1 uncharacterized protein LOC120413677 Culex pipiens pallens
XP 038118294.1 uncharacterized protein LOC6041956 Culex quinquefasciatus
XP 039446726.1 uncharacterized protein LOC120426082 Culex pipiens pallens
XP 001663557.2 uncharacterized protein LOC5577784 Aedes aegypti
