# Supplementary material for: Orthologous genes of the red flour beetle Tribolium castaneum and the vinegar fly Drosophila melanogaster
Source: BMC Genom Data. 2025 Dec 12;27:17. doi: 10.1186/s12863-025-01397-0 (PMC12903232; doi:10.1186/s12863-025-01397-0)

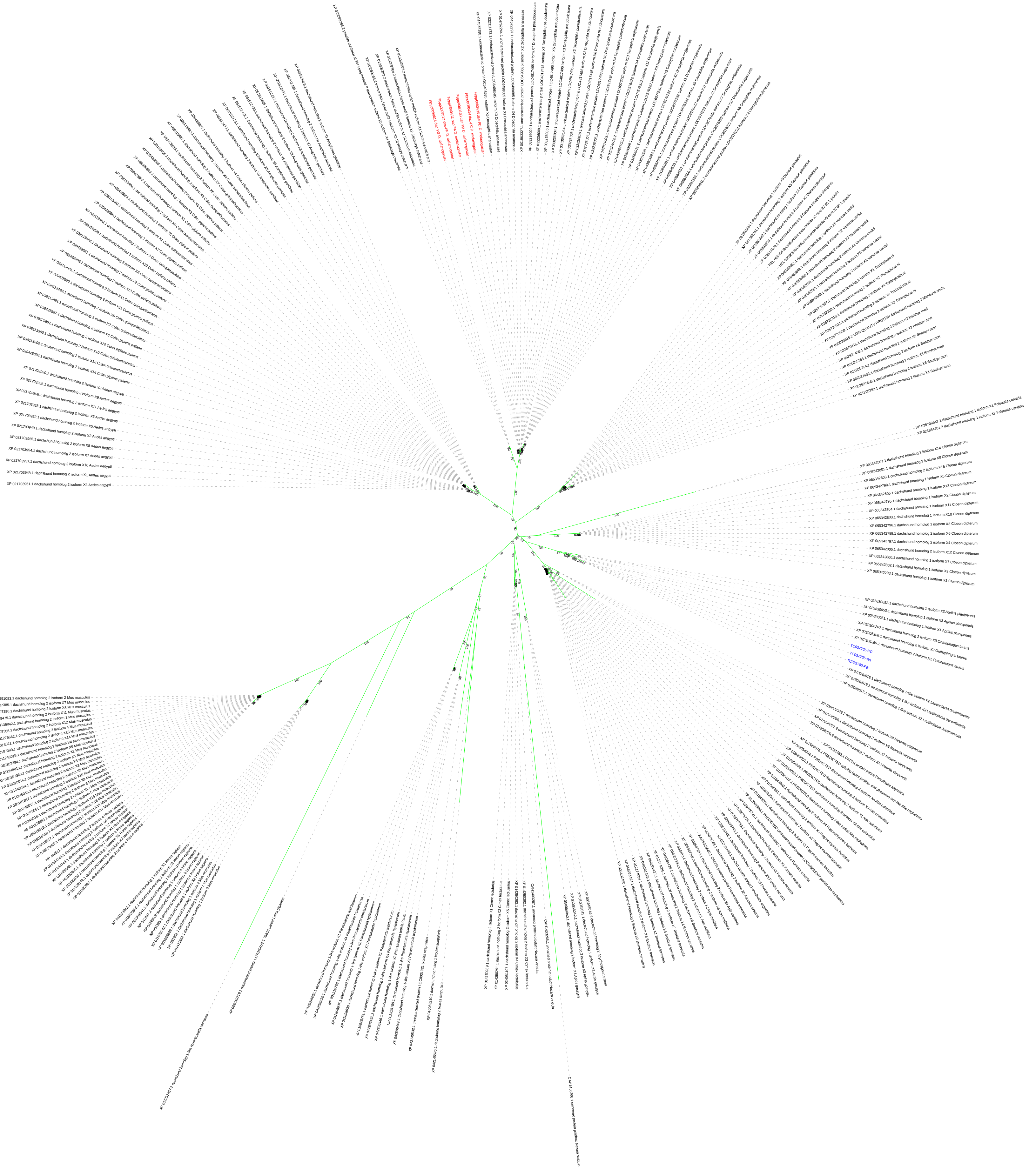

Supplement: Supplementary file 3 — Supplementary Material 3 [file dac.pdf › 3.Manually_checked_genes/4.Trees/dac.pdf]
